# Processing of *Reynoutria multiflora*: transformation of catechin and gallic acid derivatives and their identification

**DOI:** 10.3389/fphar.2024.1356876

**Published:** 2024-02-26

**Authors:** Junqi Bai, Qiyu Zou, He Su, Baosheng Liao, Ping Wang, Juan Huang, Danchun Zhang, Lu Gong, Wen Xu, Jing Zhang, Zhihai Huang, Xiaohui Qiu

**Affiliations:** ^1^ Guangdong Provincial Hospital of Traditional Chinese Medicine, The Second Clinical Medical College of Guangzhou University of Chinese Medicine, Guangzhou, China; ^2^ Shanghai Dehua Traditional Chinese Medicine Co., Ltd., Shanghai, China

**Keywords:** *Reynoutria multiflora* (Thunb.) Moldenke, gallic acid, catechin, processing, tandem mass spectrometry

## Abstract

**Introduction:** The root of *Reynoutria multiflora* (Thunb.) Moldenke (RM) has been used widely in formulations of herbal medicines in China for centuries. Raw *R*. *multiflora* (RRM) should be processed before use to reduce toxicity and increase efficacy. However, detailed regulation of the processing endpoint is lacking, and the duration of processing can vary considerably. We conducted in-depth research on stilbene glycosides in RM at different processing times. Previously, we discovered that 219 stilbene glycosides changed markedly in quantity and content. Therefore, we proposed that processing causes changes in various chemical groups.

**Methods:** To better explain the mechanism of RM processing for toxicity reduction and efficacy enhancement, we used a method of tandem mass spectrometry described previously to research gallic acid based and catechin based metabolites.

**Results:** A total of 259 metabolites based on gallic acid and 112 metabolites based on catechins were identified. Among these, the peak areas of 157 gallic acid and 81 catechins gradually decreased, those of another 71 gallic acid and 30 catechins first increased and then decreased, those of 14 gallic acid and 1 catechin gradually increased. However, 17 of the gallic acids showed no significant changes. We speculate that many gallic acid metabolites hydrolyze to produce gallic acid; moreover, the dimers/trimers of catechins, after being cleaved into catechins, epicatechin, gallic acid catechins, and epicatechin monomers, are cleaved into gallic acid and protocatechualdehyde under high temperature and high humidity, subsequently participating in the Maillard reaction and browning reactions.

**Discussion:** We showed that processing led to changes in chemical groups, clarification of the groups of secondary metabolites could provide a basis for research on the pharmacological and toxic mechanisms of RM, as well as the screening of related markers.

## 1 Introduction

The root of *Reynoutria multiflora* Thunb. (syn: *Polygonum multiflorum* Thunb.) is known as “He-shou-wu” in China. It has been used widely for centuries in traditional Chinese medicine (TCM) formulations (Li, et al., 2017). Several studies have shown that raw *R. multiflora* and its processed products have different pharmacological effects. RRM has the effects of detoxification, carbuncle elimination, and bowel relaxation. Processed *R. multiflora* (PRM) shows the effects of tonifying the liver and kidneys, and hair-blackening, and can be used in tonic medicines (Cheung, et al., 2014; Lin, et al., 2015; Chinese Pharmacopoeias commission, 2020). The difference between RRM and PRM is processing: steaming or stewing. However, detailed regulation of the processing endpoint is lacking, and the duration of processing can vary considerably. Previously (Bai et al., 2021; Bai et al., 2022), we showed that if RM was processed for 24 h then there were attributable differences between PRM and RRM. It has been shown that stilbene glycosides undergo marked changes during processing; 219 stilbene glycosides have been shown to undergo dramatic changes in number and content (not just a few chromatographic peaks on spectra). For instance, the content of 2,3,5,4′-tetrahydroxystilbene-2-O-β-D-glucoside has decreased, or the content of several other metabolites has increased or decreased, during processing (Liang, et al., 2010; Liu, et al., 2011; Chen, et al., 2016; Wang, et al., 2016).

Some researchers have reported that, under high temperatures, catechins are prone to degradation and isomerization (Yoshihiro, et al., 1993; Zulema, et al., 2004; Wang, et al., 2006). Catechins can be cracked into gallic acid. Isomerization, oxidative polymerization, and hydrolysis are important reactions that cause the chemical transformation of catechins. Isomerization of one molecule of a catechin can generate one molecule of the corresponding isomer, whereas hydrolysis of one molecule of a catechin can generate one molecule of gallic acid (Li, et al., 2018). Previously, we showed that the content of catechins and epicatechins decreased sharply (or even disappeared) during processing. What catechins are transformed into during the processing of RM and the manner of this transformation are not clear, but we believe that the answers to these questions are important for the processing of RM.

It has been reported that condensed tannins can undergo acid-catalyzed cleavage in the presence (or excess amount) of a nucleophile (Nonaka, et al., 1982; Torres et al., 2002). That is, condensed polymers are depolymerized into oligomers and monomers under thermal and acidic conditions. Oligomers and monomers (except gallic acid) are also unstable in thermal and acidic conditions. Hence, they are subject to structural transformation after additional processing, which causes catechin to be transformed into its isomeric compound: epicatechin (Ross, et al., 2011). Besides isomerization, polymers, and monomers are depolymerized further. Hence, the monomer protocatechuic aldehyde could finally be obtained after processing, and gallic acid (another derived monomer) could accumulate after each processing cycle. Studies have shown that the contents of other bioactive compounds (e.g., catechins, gallic acid, procyanidin B2) change after processing, which may lead to a change in their therapeutic effects (Yao, et al., 2006; Chen, et al., 2012; Han, et al., 2013; Li, et al., 2018).

PRM at 24 h was clearly different from RRM, we conducted more in-depth analyses of the important chemical metabolites of RM (stilbene glycosides) and studied changes in the peak area with the duration of processing (Bai et al., 2021). A total of 219 stilbene glycosides underwent marked changes in number and content (not only a few chromatographic peaks), so we continued to conduct in-depth research on metabolites related to gallic acid and catechins. First, we identified the changes in the number of these two types of metabolites. Second, we evaluated the change process and mechanism of the gradual deepening of color during the processing of RM.

## 2 Materials and methods

### 2.1 Materials and sample processing

RRM materials were collected from Deqing (Guangzhou, China), Miyi (Sichuan, China), Kaili (Guizhou, China), the geo-authentic producing area of RM. There were three batches (30 kg each) from each producing area. The corresponding batch numbers, producer, and image information were documented (Supplementary Table S1). Samples were authenticated by Professor Zhihai Huang, and voucher specimens were deposited in the Materials Medica Preparation Laboratory of the Second Affiliated Hospital of the Guangzhou University of Chinese Medicine (Guangzhou, China).

Galic acid, catechin and epicatechin were purchased from China institute for food and drug control (No. 110831-201906, 110877-202005, 110878-201703, beijing, China). Acetonitrile and methanol (HPLC grade), were supplied by E. Merck (Darmstadt, Germany), formic acid (HPLC grade) was purchased from Fisher Scientific (Massachusetts, United States), ultra-pure water was prepared by a Mili-Q water purification system (Millipore, MA, United States).

### 2.2 Preparation of samples

#### 2.2.1 Processing

According to the processing technology we have researched earlier (Bai et al., 2021), Each batch of RRM was first moistened with black bean juice, and then steamed for 32 h. At the time of steaming for 4 h, 8 h, 12 h, 18 h, and 24 h, 2 kg of RRM were taken out and dried to obtain PRM of different times. Samples were processed by Shanghai Dehua Traditional Chinese Medicine Co., Ltd.

#### 2.2.2 Extraction

All the samples were prepared using following method: 1 g sample powder was ultrasonicated for 30 min with 25 mL of 70% ethanol, followed by filtration and then evaporated the filtrate. 5 mL of ultrapure water were added to dissolve the residue and then extracted twice with 15 mL of ethyl acetate. The resulting mixture was combined with an ethyl acetate solution and evaporated over a water bath; after that, 1 mL of methanol was added to dissolve the residue and centrifugation (15,000 rpm, 4°C) for 10 min by a 1.5 mL centrifuge tube. Finally, the supernatant of the treatment samples was injected into the UPLC-Q-Exactive plus orbitrap MS/MS system.

### 2.3 UPLC-tandem Q-exactive plus orbitrap mass spectrometry analysis

All samples were analysed using an Ultimate 3000 UPLC system (Dionex, United States) that was controlled with Thermo Xcalibur software (Thermo Fisher Scientific, United States). The samples were separated using a Kinetex UPLC C18 column (100*2.1 mm, 1.7 µm) (Phenomenex, United States). The mobile phase consisted of solvent A (0.1% formic acid) and solvent B (acetonitrile). A gradient elution was applied using the following optimized gradient program: 8%–8% B at 0–3 min, 8%–28% B at 3–25 min, 28%–40% B at 25–26 min, 40%–50% B at 26–28 min, 50%–70% B at 28–30 min, 70%–90% B at 30–32 min, and 90%–90% B at 32–35 min. The flow rate was kept at 0.4 mL/min, the sample injection volume was 1 μL, and the column temperature was maintained at 25°C. Mass spectrometry (MS) was undertaken on a Q-Exactive Plus™ Quadrupole-Orbitrap mass spectrometer (Thermo Fisher Scientific, Waltham, MA, United States) in negative ion mode. The scan mass range was set at *m/z* 100–1,200. The parameter settings were: a full scan and fragment spectral resolution of 70,000 full width at half maximum (FWHM) and 17,500 FWHM, respectively; capillary temperature was 350°C; temperature of the auxiliary gas heater was 350°C; spray voltage was −3.2 KV; sheath-gas flow rate was 40 Arb; auxiliary-gas flow rate was 15 Arb; S-lens RF level was set at 50. The acquisition mode of stepped normalized collision energy was 30, 50, and 70 eV. The accumulated resultant fragment ions were injected into the mass spectrometer for single-scan detection.

## 3 Results

### 3.1 Base peak chromatograms

**FIGURE 1:**
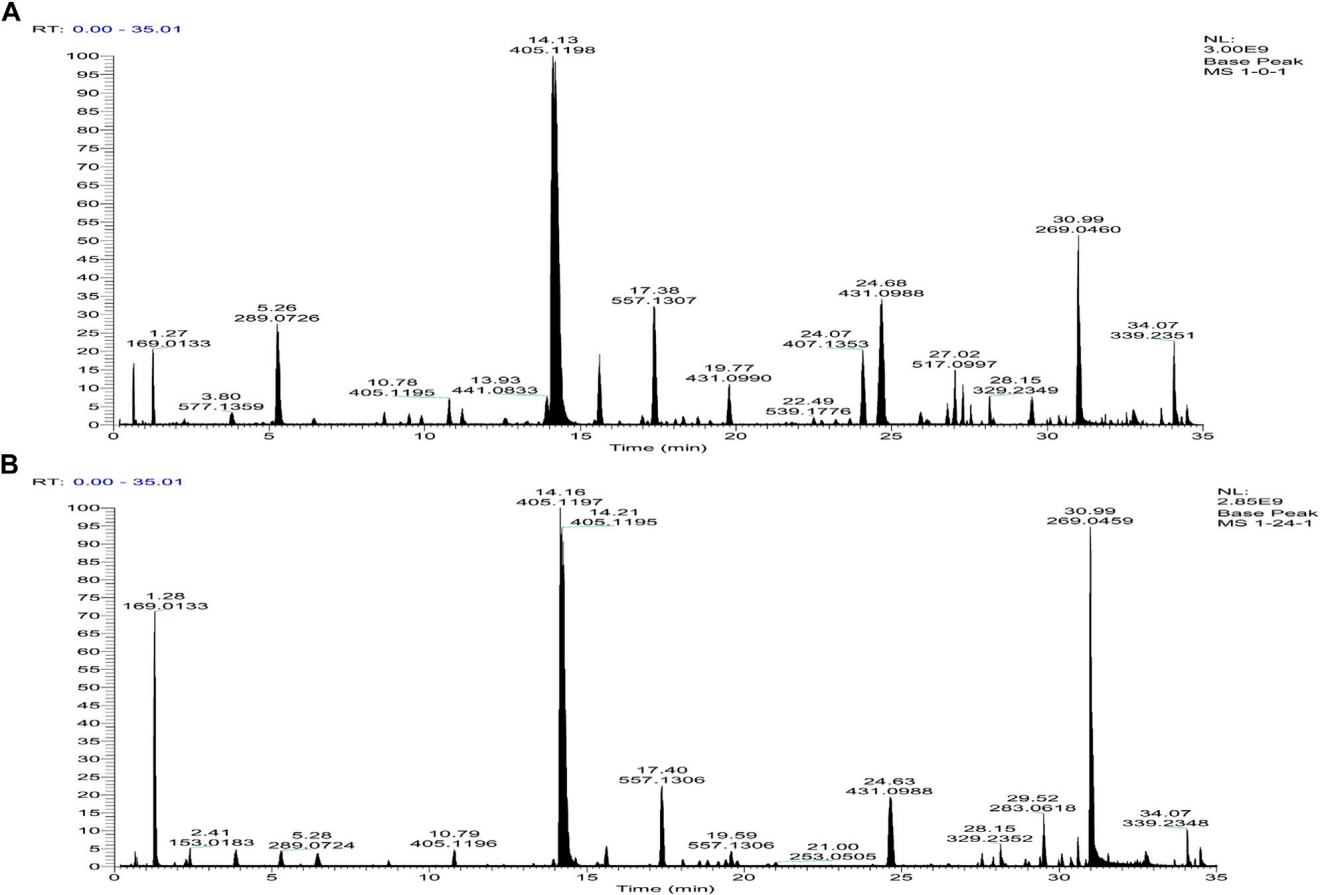
Base peak intensity chromatograms of samples of raw *R. multiflora* Thunb. [RRM, **(A)**] and processed *Reynoutria multiflora* Thunb. [PRM for 24 h, **(B)**] derived from MS^2^ using the UHPLC-Q-Exactive Plus Orbitrap mass spectrometer.

The chemical profiles of RRM and PRM were analyzed by tandem mass spectrometry (MS^2^) using an UHPLC-Q-Exactive Plus Orbitrap mass spectrometer. The representative base peak chromatograms of RRM and PRM (24 h) are shown in Figure 1.

### 3.2 Fragmentation pathway of gallic acid and derivatives

**FIGURE 2:**
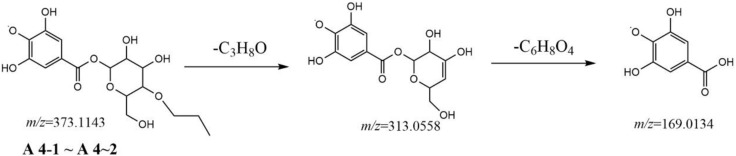
Postulated fragmentation pathways of metabolites A4-1 to A4-2.

**FIGURE 3:**
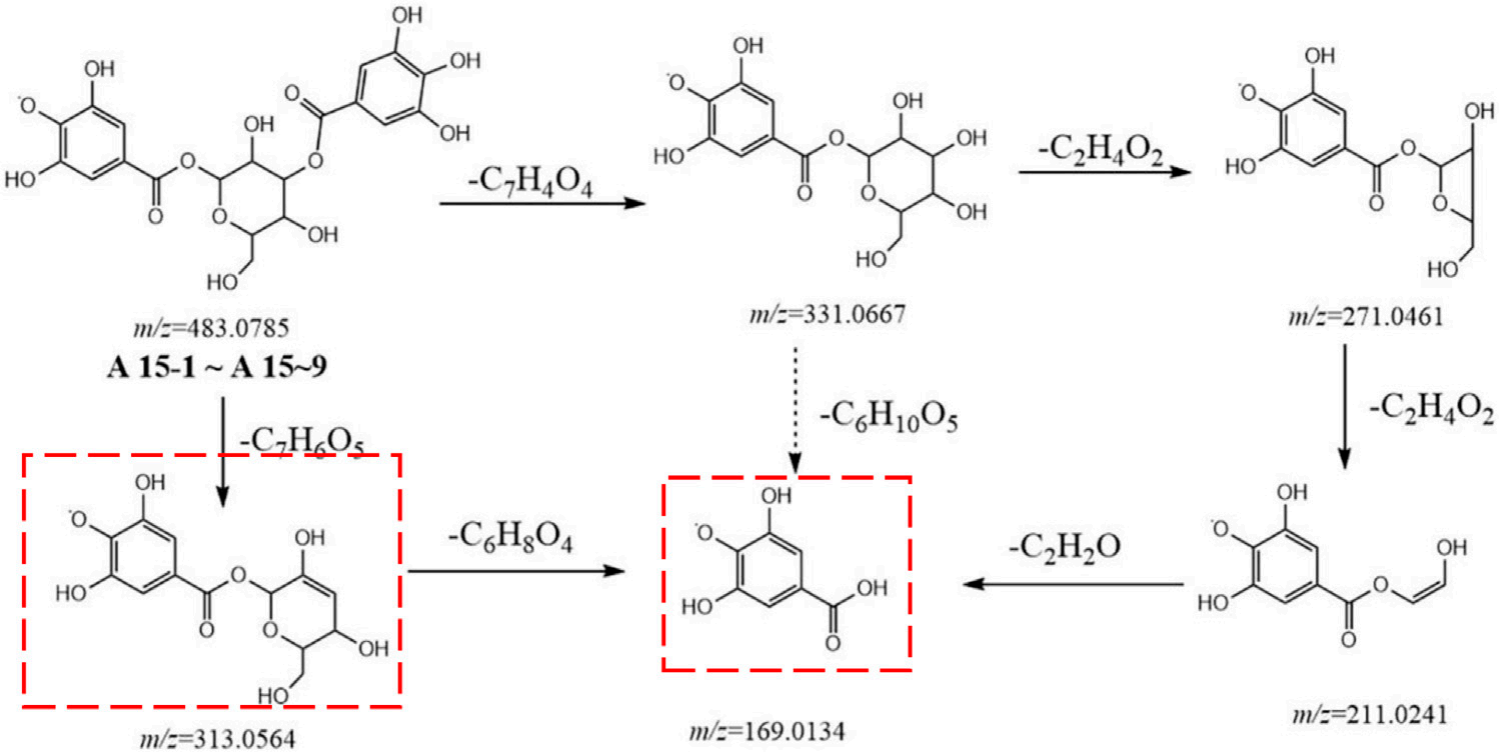
Postulated fragmentation pathways of metabolites A15-1 to A15-9. The ion fragments in the red box are characteristic ion fragments of gallic-acid metabolites.

**FIGURE 4:**
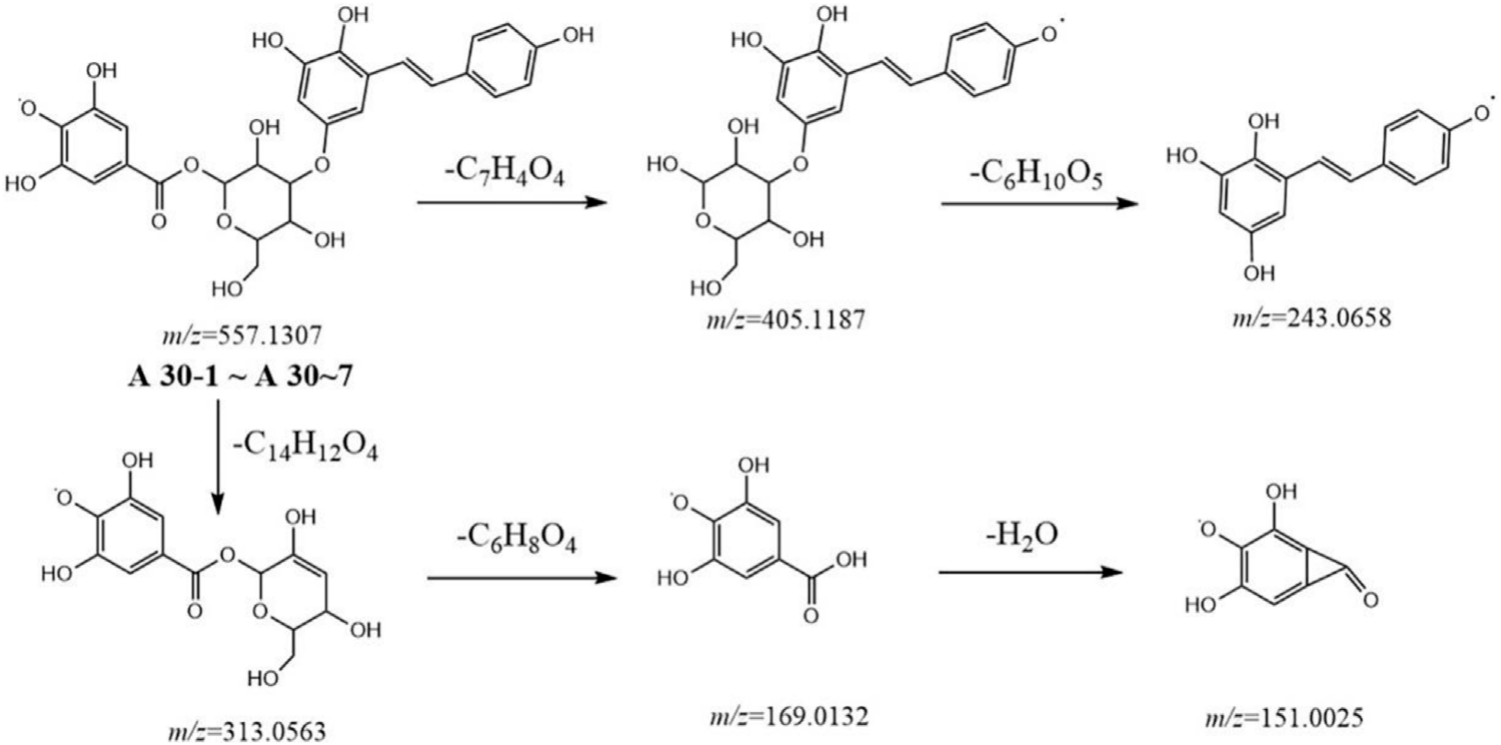
Postulated fragmentation pathways of metabolites A30-1 to A30-7.

**FIGURE 5:**
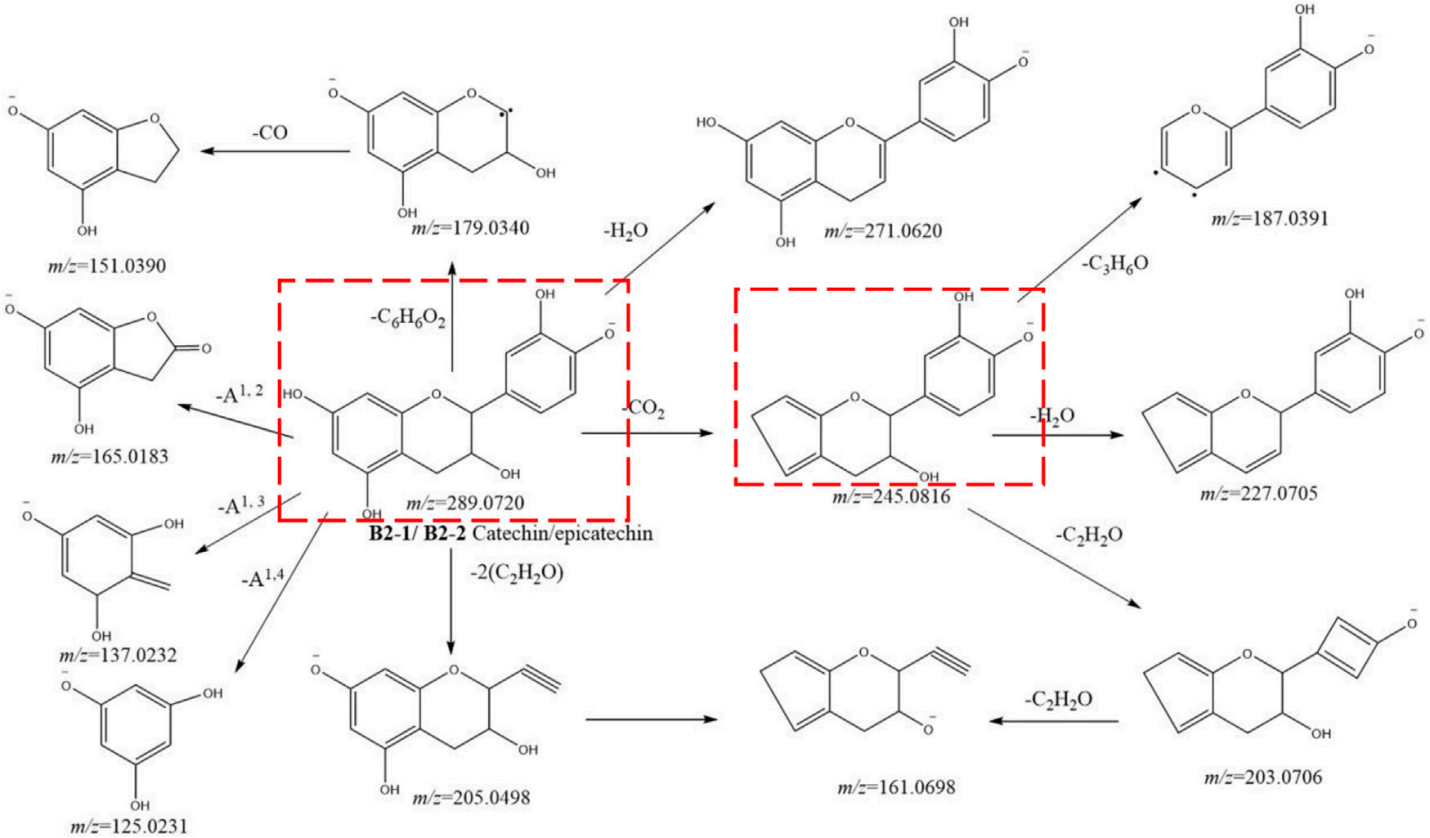
Postulated fragmentation pathways of metabolites B2-1 to B2-2. The ion fragments in the red box are characteristic ion fragments of monomer catechin metabolites.

To identify the derivatives of gallic acid in the processing of RM, a standard of gallic acid was first analyzed by MS^2^ using the UPLCQ-Exactive Plus Orbitrap mass spectrometer under the condition mentioned above. Gallic acid (**A1-1**, retention time (t_R_) = 1.28 min) had a [M-H]^−^ ion at *m/z* 169.0133 (C_7_H_5_O_5_) with only a dominant ion at *m/z* 125.0231 (C_6_H_5_O_3_) in MS^2^ spectrum, but a difference of 44 Da between the mass of the precursor ion and product ion, which involved a neutral loss of CO_2_. These two ions could be used as diagnostic ions to identify gallic acid. Metabolite **A1-2** (t_R_ = 2.03 min) also had an [M-H]^−^ ion at *m/z* 169.0133 (C_7_H_5_O_5_), and showed a fragment ion at *m/z* 125.0231 in MS^2^ spectrum, indicated that it was an isomer of gallic acid. In the present study, most of the metabolites were formed by dehydration of gallic acid glycosides and other small molecules, gallic acid and different substituents are linked to different hydroxyl groups of glucose. Under electron bombardment, substituents and glucose are cleaved, and the oxygen linked to glucose and substituents breaks off from glucose, forming glucose residues (161.04) and glycosides formed by dehydration of gallic acid (152.01). Therefore, the ion formed after the substituent is 313.0564 instead of 331.0667. As shown in Figures 2–5.

Metabolites **A2-1, A2-2**, and **A2-3** showed the same [M-H]^−^ ion at *m/z* 183.0292 (C_8_H_7_O_5_) and MS^2^ spectra gave ions at *m/z* 183.0292 (C_8_H_7_O_5_), 168.0053(C_7_H_4_O_5_), and 124.0147 (C_6_H_4_O_3_) from a continuous loss of CH_3_ and CO_2_. Thus, metabolites **A2-1, A2-2**, and **A2-3** were identified as methyl gallate.

Metabolites **A3-1**, **A3-2**, **A3.3**, and **A3-4** showed the same [M-H]^−^ ion at *m/z* 331.0673 (C_13_H_15_O_10_) and MS^2^ spectra gave identical ions at *m/z* 331.0667 (C_13_H_15_O_10_), 271.0461 (C_11_H_11_O_8_), 211.0241 (C_9_H_7_O_6_), and 169.0132 (C_7_H_5_O_5_), respectively. Comparison with the literature revealed metabolites **A3-1** to **A3-4** to be gallic acid-O-glycoside.

Metabolites **A4-1** and **A4-2** showed the same [M-H]^−^ ion at *m/z* 373.1143 (C_16_H_21_O_10_) and MS^2^ spectra gave identical ions at *m/z* 313.0558 (C_13_H_13_O_9_) and 169.0134 (C_7_H_5_O_5_), respectively. Comparison with the literature revealed metabolites **A4-1** and **A4-2** to be gallic acid-O-glycoside-O-propanoyl. A fragmentation pathway was postulated (Figure 2). Similarly, metabolites **A5-1**, **A5-2**, and **A5-3** were characterized tentatively to be gallic acid-O-glycoside-O-hydroxyphenyl because of the [M-H]^−^ ion at *m/z* 423.0924 (C_19_H_19_O_12_) and because MS^2^ spectra gave ions at *m/z* 313.0558 (C_13_H_13_O_9_), 169.0134 (C_7_H_5_O_5_), and 125.0231 (C_6_H_5_O_3_), respectively.

Metabolites **A9-1** to **A9-8** showed the same [M-H]^−^ ion at *m/z* 453.1048 (C_20_H_21_O_12_) and MS^2^ spectra gave identical ions at *m/z* 313.0563 (C_13_H_13_O_9_) and 169.0134 (C_7_H_5_O_5_). The molecular formula of the substituent should be C_7_H_8_O_3_, the most likely was methoxycatechol. Therefore, metabolites **A9-1** to **A9-8** were determined to be gallic acid-O-glycoside-O-methoxycatechol acyl. Similarly, metabolites **A10-1** to **A10-5** were characterized tentatively to be gallic acid-O-glycoside-O-dimethoxycatechol acyl because of the [M-H]^−^ ion at *m/z* 467.1196 (C_21_H_23_O_12_) and because MS^2^ spectra gave ions at *m/z* 313.0563 (C_13_H_13_O_9_), 169.0134 (C_7_H_5_O_5_), and 153.0545 (C_8_H_9_O_3_). The molecular formula of the substituent could be C_8_H_10_O_3_, which was 14 Da (CH_2_) higher than that of metabolites **A9**, so it was determined to be dimethoxyphenol.

Metabolites **A11-1** to **A11-4** showed the same [M-H]^−^ ion at *m/z* 477.1038 (C_22_H_21_O_12_) and MS^2^ spectra gave identical ions at *m/z* 313.0563 (C_13_H_13_O_9_), 169.0134 (C_7_H_5_O_5_), 163.0390 (C_9_H_7_O_3_), and 125.0231 (C_6_H_5_O_3_). The molecular formula of substituent should be C_9_H_8_O_3_, It may be hydroxycinnamic acid. Therefore, metabolites **A11-1** to **A11-4** were determined as gallic acid-O-glycoside-O-hydroxycinnamoyl. Similarly, metabolites **A12-1** to **A12-9** tentatively characterized to be gallic acid-O-glycoside-O-hydroxyphenyl propionyl. Since the [M-H]^−^ ion at *m/z* 479.1198 (C_22_H_23_O_12_) and MS^2^ spectra gave ions at *m/z* 313.0563 (C_13_H_13_O_9_), 169.0134 (C_7_H_5_O_5_), 165.0547 (C_9_H_9_O_3_), and 153.0545 (C_7_H_5_O_3_). The molecular formula of the substituent may be C_9_H_10_O_3_, which was 2 Da (H_2_) higher than that of metabolites **A11**, the substituent may be hydroxyphenylpropionic acid.

Metabolites **A13-1** to **A13-6** gave an [M-H]^−^ ion at *m/z* 481.0990 (C_21_H_21_O_13_) and product ions at *m/z* 313.0563 (C_13_H_13_O_9_) and 169.0134 (C_7_H_5_O_5_). The molecular formula of the substituent may be C_8_H_8_O_4_, the substituent may be hydroxymethoxybenzoic acid. Therefore, metabolites **A13-1** to **A13-6** were determined as gallic acid-O-glycoside-O-hydroxymethoxybenzoyl.

Metabolites **A14-1** and **A14-2** were tentatively characterized as gallic acid-O-glycoside-O- hydroxymethoxyphenylethanol acyl because of the [M-H]^−^ ion at *m/z* 481.1353 (C_22_H_25_O_12_) and because MS^2^ spectra gave ions at *m/z* 313.0563 (C_13_H_13_O_9_) and 169.0134 (C_7_H_5_O_5_). The molecular formula of the substituent may be C_9_H_12_O_3_, the substituent may be hydroxymethoxyphenylethanol.

Metabolites **A15-1** to **A15-9** displayed a high resolution [M-H]^−^ ion at *m/z* 483.0785 and gave an element composition of C_20_H_19_O_14_, product ions at *m/z* 331.0667 (C_13_H_15_O_10_), 313.0564 (C_13_H_13_O_9_), 271.0461 (C_11_H_11_O_8_), 211.0241 (C_9_H_7_O_6_), and 169.0134 (C_7_H_5_O_5_). The fragment ion *m/z* 331.0667 was obtained from 152 Da (C_7_H_4_O_4_) loss of parent ion, suggesting that the substituent was galloyl. Other fragment ions were similar to metabolites **A4**. Therefore, metabolites **A15-1** to **A15-9** were identified to be gallic acid-O-glycoside-O-galloyl (Figure 3).

Metabolites **A16-1** to **A16-6** displayed a high resolution [M-H]^−^ ion at *m/z* 493.1352 and gave an element composition of C_23_H_25_O_12_, product ions at *m/z* 313.0563 (C_13_H_13_O_9_), 179.0704 (C_10_H_11_O_3_), 169.0134 (C_7_H_5_O_5_), and 151.0022 (C_7_H_3_O_4_). The molecular formula of the substituent was C_8_H_10_O_3_, which was 14 Da (CH_2_) higher than that of metabolites **A12**, and it was determined to be methoxyphenylpropionic acid. Therefore, metabolites **A16-1** to **A16-6** were determined to be gallic acid-O-glycoside-O-methoxyphenylpropionyl. Similarly, metabolites **A17-1** and **A17-2** were tentatively characterized as gallic acid-O-glycoside-O-hydroxy-methoxy-phenylpropanol acyl, since the [M-H]^−^ ion at *m/z* 495.1512 (C_23_H_27_O_12_) and MS^2^ spectra gave ions at *m/z* 313.0563 (C_13_H_13_O_9_) and 169.0134 (C_7_H_5_O_5_).

Metabolite **A18** gave an [M-H]^−^ ion at *m/z* 497.0941 (C_21_H_21_O_14_) and product ions at *m/z* 345.0823 (C_14_H_17_O_10_), 313.0558 (C_13_H_13_O_9_), 183.0291 (C_8_H_7_O_5_), and 169.0134 (C_7_H_5_O_5_). Fragment ions *m/z* 313.0558 and 169.0134 followed to the above rules, fragment ion *m/z* 345.0823 was obtained by loss of a galloyl, and fragment ion *m/z* 183.0291 was the substituent. Therefore, metabolite **A18** was tentatively identified to be gallic acid-O-glycoside-O-methoxyphenoyl. Similarly, metabolites **A19-1** and **A19-2** were tentatively characterized to be gallic acid-O-glycoside-O-trimethoxyphenol acyl because of the [M-H]^−^ ion at *m/z* 497.1305 (C_22_H_25_O_12_) and MS^2^ spectra gave ions at *m/z* 313.0563 (C_13_H_13_O_9_), 169.0134 (C_7_H_5_O_5_), and 151.0024 (C_7_H_3_O_4_).

Metabolites **A20-1** to **A20-6** showed a high resolution [M-H]^−^ ion at *m/z* 503.1201 and gave an element composition of C_24_H_23_O_12_, product ions at *m/z* 313.0563 (C_13_H_13_O_9_), 189.0547 (C_11_H_9_O_3_), 169.0134 (C_7_H_5_O_5_), and 151.0024 (C_7_H_3_O_4_). Therefore, metabolites **A20-1** to **A20-6** were determined to be gallic acid-O-glycoside-O-dimethyl-hydroxycoumarin acyl. Similarly, metabolites **A21-1** to **A21-4** were tentatively characterized to be gallic acid-O-glycoside-O-carboxy-cinnamoyl because of the [M-H]^−^ ion at *m/z* 505.0994 (C_23_H_21_O_13_) and MS^2^ spectra gave ions at *m/z* 313.0563 (C_13_H_13_O_9_), 191.0341 (C_10_H_7_O_4_), and 169.0134 (C_7_H_5_O_5_). Metabolites **A22-1** and **A22-2** were characterized to be gallic acid-O-glycoside-O-hydroxy-methoxy-cinnamoyl, since the [M-H]^−^ion at *m/z* 507.1145 (C_23_H_23_O_13_) and MS^2^ spectra gave ions at *m/z* 313.0563 (C_13_H_13_O_9_), 193.0499 (C_10_H_9_O_4_), and 169.0134 (C_7_H_5_O_5_).Then, metabolites **A23-1** and **A23-2** were tentatively identified to be gallic acid-O-glycoside-O-dimethoxy-phenylacetate acyl, because of the [M-H]^−^ ion at *m/z* 509.1302 (C_23_H_25_O_13_) and because MS^2^ spectra gave ions at *m/z* 313.0563 (C_13_H_13_O_9_), 195.0659 (C_10_H_11_O_4_), and 169.0134 (C_7_H_5_O_5_).

Metabolite **A24** displayed a high resolution [M-H]^−^ ion at *m/z* 511.1095 and gave an element composition of C_22_H_23_O_14_, product ions at *m/z* 467.1206 (C_19_H_13_O_6_), 313.0563 (C_13_H_13_O_9_), and 169.0134 (C_7_H_5_O_5_). Therefore, metabolite **A24** was tentatively identified to be gallic acid-O-glycoside-O-hydroxy-dimethoxybenzoyl.

Metabolites **A25-1** to **A25-6** were eluted at 16.40 min, 16.99 min, 17.77 min, 18.40 min, 18.78 min, and 19.69 min. They both showed an accurate [M-H]^−^ ion at *m/z* 541.1359 (C_26_H_27_O_12_). In their MS^2^ spectra, the [M-H]^−^ ion showed fragment ions at *m/z* 313.0563 (C_13_H_13_O_9_), 243.0661 (C_14_H_11_O_4_), 227.0707 (C_14_H_11_O_3_), and 169.0134 (C_7_H_5_O_5_). Metabolites **A25-1** to **A25-6** were identified to be gallic acid-O-glycoside-O-trihydroxystilbene. Similarly, metabolites **A26-1** and **A26-2** were characterized to be gallic acid-O-glycoside-O-dihydrotrihydroxystilbene. Since the [M-H]^−^ ion at *m/z* 543.1513 (C_27_H_27_O_12_) and MS^2^ spectra gave ions at *m/z* 313.0563 (C_13_H_13_O_9_), 229.0865 (C_14_H_13_O_3_), and 169.0134 (C_7_H_5_O_5_).

Metabolites **A27-1** to **A27-8** displayed a high resolution [M-H]^−^ ion at *m/z* 545.1306 and gave an element composition of C_26_H_25_O_13_. MS^2^ spectra gave ions at *m/z* 313.0563 (C_13_H_13_O_9_), 231.0658 (C_13_H_11_O_4_), and 169.0134 (C_7_H_5_O_5_). Comparison with the literature revealed metabolites **A27-1** to **A27-8** were tentatively identified to be gallic acid-O-glycoside-O-hydroxymusizin.

Metabolites **A29-1** to **A29-5** showed a high resolution [M-H]^−^ ion at *m/z* 555.1508 and gave an element composition of C_28_H_27_O_12_. MS^2^ spectra gave ions at *m/z* 313.0563 (C_13_H_13_O_9_), 241.0865 (C_15_H_13_O_3_), and 169.0134 (C_7_H_5_O_5_). Comparison with the literature revealed metabolites **A29-1** to **A29-5** were tentatively identified as gallic acid-O-glycoside-O-tetrahydroxyphenanthryl. Similarly, metabolites **A30-1** to **A30-7** were characterized to be gallic acid-O-glycoside-O-tetrahydroxystilbene-acyl (Figure 4), because of the [M-H]^−^ ion at *m/z* 557.1307 (C_27_H_25_O_13_) and because MS^2^ spectra gave ions at *m/z* 405.1187 (C_20_H_21_O_9_), 313.0563 (C_13_H_13_O_9_), 243.0658 (C_14_H_11_O_4_), and 169.0134 (C_7_H_5_O_5_).

Metabolites **A 31-1** to **A 31-4** gave precursor ion [M-H]^−^ at *m/z* 559.1460 (C_27_H_27_O_13_) and eluted at 16.02 min, 25.60 min, 26.70 min, 27.66 min. In MS^2^ spectra, the [M-H]^−^ ions showed at *m/z* 559.1460 (C_27_H_27_O_13_), 407.1342 (C_20_H_23_O_9_), 313.0563 (C_13_H_13_O_9_), 245.0817 (C_14_H_13_O_4_), 169.0134 (C_7_H_5_O_5_), and 139.0386 (C_7_H_7_O_3_). By comparison with the literature, metabolites **A 31-1** to **A 31-4** were tentatively characterized to be gallic acid-O-glycoside-O-dihydrotetrahydroxystilbeneacyl.

Metabolite **A32** displayed a high resolution [M-H]^−^ ion at *m/z* 563.1170 and gave an element composition of C_29_H_23_O_12_, product ions at *m/z* 313.0563 (C_13_H_13_O_9_), 249.0533 (C_15_H_10_O_3_), and 169.0134 (C_7_H_5_O_5_). The substituent was suggested to be hydroxy-methyl-anthraquinone by comparison with literature. Metabolite **A32** was tentatively identified to be gallic acid-O-glycoside-O-hydroxy-methyl-anthraquinoyl. Similarly, metabolite **A33** was characterized to be gallic acid-O-glycoside-O-emodin anthrone acyl, since the [M-H]^−^ ion at *m/z* 569.1309 (C_28_H_25_O_13_) and MS^2^ spectra gave ions at *m/z* 313.0563 (C_13_H_13_O_9_), 255.0663(C_15_H_11_O_4_), and 169.0134 (C_7_H_5_O_5_). By comparison with literature, the substituent was suggested to be emodin anthrone. Metabolites **A34-1**, **A34-2**, and **A34-3** were characterized to be gallic acid-O-glycoside-O-salicylate diester, because of the [M-H]^−^ ion at *m/z* 571.1094 (C_27_H_23_O_14_) and MS^2^ spectra gave ions at *m/z* 419.0987 (C_20_H_19_O_10_), 313.0563 (C_13_H_13_O_9_), 257.0453 (C_15_H_13_O_4_), and 169.0134 (C_7_H_5_O_5_). The substituent was suggested to be a salicylate diester.

Metabolites **A35-1** to **A35-5** showed a high resolution [M-H]^−^ ion at *m/z* 573.1259 and gave an element composition of C_27_H_25_O_14_. MS^2^ spectra gave ions at *m/z* 313.0563 (C_13_H_13_O_9_), 259.0610 (C_14_H_11_O_5_), and 169.0134 (C_7_H_5_O_5_). Comparison with the literature revealed metabolites **A35-1** to **A35-5** were tentatively identified as gallic acid-O-glycoside-O-tetrahydroxyphenanthryl.

Metabolites **A36-1** to **A36-9** displayed a high resolution [M-H]^−^ ion at *m/z* 583.1102 and gave an element composition of C_28_H_23_O_14_, product ions at *m/z* 583.1102 (C_28_H_23_O_14_), 431.0983 (C_21_H_19_O_10_), 313.0564 (C_13_H_13_O_9_), 269.0458 (C_15_H_9_O_5_), and 169.0134 (C_7_H_5_O_5_). Among these, fragment ion 431.0983 was obtained from the loss of 152 Da (C_7_H_4_O_4_) of the parent ion, suggesting that the substituent was gallic acid. The ion of *m/z* 269.0458 suggested that it was emodin. Thus, metabolites **A36-1** to **A36-9** were identified to be gallic acid-O-glycoside-O-emodin acyl.

Metabolites **A37-1** to **A37-6** gave a [M-H]^−^ ion at *m/z* 585.1248 (C_28_H_25_O_14_) and product ions at *m/z* 585.1248 (C_28_H_25_O_14_), 541.1355(C_27_H_25_O_12_), 313.0563 (C_13_H_13_O_9_), and 169.0134 (C_7_H_5_O_5_). The lion of 585.1248 was 44 Da (CO_2_) higher than that of metabolites **A25**, and other fragment ions were consistent with metabolites **A25**. Therefore, metabolites **A37-1** to **A37-6** were determined to be gallic acid-O-glycoside-O-tetrahydroxystilbene-COOH.

Metabolites **A38-1** to **A38-7** were characterized as gallic acid-O-glycoside-O-afzelechin acyl, as the [M-H]^−^ ion *m/z* 587.1415 (C_28_H_27_O_14_) and MS^2^ ions at *m/z* 313.0563 (C_13_H_13_O_9_), 273.0769 (C_15_H_13_O_5_), 169.0134 (C_7_H_5_O_5_), and 149.0232 (C_8_H_5_O_3_).

Metabolites **A39-1** to **A39-4** showed the same [M-H]^−^ ion at *m/z* 589.1206 (C_27_H_25_O_15_) and MS^2^ spectra gave ions at *m/z* 465.0675 (C_20_H_17_O_13_), 437.1085 (C_20_H_21_O_11_), 313.0564 (C_13_H_13_O_9_), and 169.0136 (C_7_H_5_O_5_). MS^2^ showed that the ions *m/z* 465.0675 and 437.1085 of C_7_H_8_O_2_ and C_7_H_14_O_4_ were lost due to the hydroxybenzene hexanol and gallic acid moieties. By literature comparison, metabolites **39-1** to **39-4** were identified to be gallic acid-O-glycoside-O- hexahydroxystilbene.

Metabolites **A40-1** to **A40-4** showed the same [M-H]^−^ ion at *m/z* 597.1258 (C_29_H_25_O_14_) and MS^2^ spectra gave ions at *m/z* 313.0564 (C_13_H_13_O_9_), 283.0612 (C_16_H_11_O_5_), 269.0457 (C_15_H_9_O_5_), and 169.0132 (C_7_H_5_O_5_). Therefore, metabolites **A40-1** to **A40-4** were determined to be gallic acid-O-glycoside-O-physcion. Similarly, metabolites **A41-1** and **A41-2** were characterized to be gallic acid-O-glycoside-O-hydroxyemodin. The [M-H]^−^ ion at *m/z* 599.1049 (C_28_H_23_O_15_) and MS^2^ spectra gave ions at *m/z* 313.0563 (C_13_H_13_O_9_), 255.0663(C_15_H_11_O_4_), and 169.0134 (C_7_H_5_O_5_).

Metabolites **A42-1** and **A42-2** showed the same [M-H]^−^ ion at *m/z* 589.1206 (C_27_H_25_O_15_) and MS^2^ spectra gave ions at *m/z* 465.0675 (C_20_H_17_O_13_), 437.1085 (C_20_H_21_O_11_), 313.0564 (C_13_H_13_O_9_), and 169.0136 (C_7_H_5_O_5_). MS^2^ showed that the ions *m/z* 465.0675 and 437.1085 were losses of C_7_H_8_O_2_ and C_7_H_14_O_4_ due to hydroxybenzene hexanol and gallic acid moiety. Fragment ions *m/z* 465.0675, 313.0564, 169.0136 were the determination ion of di-gallic acid glucoside. By literature comparison, metabolites **A42-1** and **A42-2** were identified to be eriodictyol 7-O-(6″-O-galloyl)-β-D-glucopyranoside.

Metabolites **A43-1** to **A43-6** gave a [M-H]^−^ ion at *m/z* 603.1364 (C_28_H_27_O_15_) and product ions at *m/z* 313.0563 (C_13_H_13_O_9_), 289.0718 (C_15_H_13_O_6_), and 169.0134 (C_7_H_5_O_5_). The substituent can be catechin or epicatechin. Their relative retention time were 5.45 min, 8.82 min, 9.33 min, 10.43 min, 10.76 min, and 11.32 min. According to the retention time of catechin and epicatechin, the substituents of **A43-1**, **A43-2**, and **A43-3** should be catechin, and the substituents of **A43-4**, **A43-4** and **A43-5** should be epicatechin. Therefore, metabolites **A43-1** ∼ **A43-3** were determined to be gallic acid-O-glycoside-O-catechin, and metabolites **A43-4** ∼ **A43-6** were determined to be gallic acid-O-glycoside-O-epicatechin.

Metabolite **A45** gave a [M-H]^−^ ion at *m/z* 605.1156 (C_27_H_25_O_16_) and product ions at *m/z* 465.0681(C_20_H_17_O_13_), 313.0558(C_13_H_13_O_9_), 169.0134 (C_7_H_5_O_5_), and 125.0225 (C_6_H_5_O_3_). Therefore, metabolite **A45** was tentatively identified as di-gallic acid-O-glucoside-O-benzyl alcohol. Metabolites **A47-1** to **A47-5** displayed a high resolution [M-H]^−^ ion at *m/z* 613.1207 and gave an element composition of C_29_H_25_O_15_. MS^2^ spectra gave ions at 613.1201 (C_29_H_25_O_15_), 569.1323 (C_28_H_25_O_13_), 313.0563 (C_13_H_13_O_9_), 299.0562 (C_16_H_11_O_6_), 255.0665 (C_15_H_11_O_4_), and 169.0134 (C_7_H_5_O_5_). By literature comparison, metabolites **A47-1** to **A47-5** were tentatively identified as gallic acid-O-glycoside-O-questinol.

Metabolite **A48** gave an [M-H]^−^ ion at *m/z* 617.1157 (C_28_H_25_O_16_) and product ions at *m/z* 599.1043 (C_28_H_23_O_15_), 313.0558 (C_13_H_13_O_9_), 303.0513 (C_15_H_11_O_7_), 285.0410 (C_15_H_9_O_6_), 169.0134 (C_7_H_5_O_5_), and 125.0225 (C_6_H_5_O_3_). By comparison with the literature, metabolite **A48** was tentatively characterized as taxillusin. Similarly, metabolites **A49-1** and **A49-2** showed a [M-H]^−^ion at *m/z* 619.1075 (C_31_H_23_O_14_) and MS^2^ spectra gave ions at *m/z* 313.0558 (C_13_H_13_O_9_), 305.0427 (C_18_H_9_O_5_), 271.0462 (C_15_H_11_O_7_), and 169.0134 (C_7_H_5_O_5_). By comparison with the literature, metabolites **A49-1** and **A49-2** were tentatively characterized to be gallic acid-O-glycoside-O-trihydroxynaphthacenequinone.

Metabolite **A50** gave an [M-H]^−^ ion at *m/z* 629.1158 (C_29_H_25_O_16_) and product ions at *m/z* 477.1046 (C_22_H_21_O_12_), 465.0681(C_20_H_17_O_13_), 313.0558 (C_13_H_13_O_9_), 303.0513 (C_15_H_11_O_7_), 285.0410 (C_15_H_9_O_6_), 169.0134 (C_7_H_5_O_5_), and 125.0225 (C_6_H_5_O_3_). Fragment ions *m/z* 465.0681, 313.0558, and 169.0134 indicated that there were two gallic acid substitutions and fragment ions *m/z* 477.1046, 313.0558, and 169.0134 indicated that there was also a *p*-coumaric acid substituent. Therefore, metabolite **A50** was tentatively characterized as di-gallic acid-O-glycoside-O-coumaric acid.

Metabolites **A52-1** and **A52-2** showed the same [M-H]^−^ ion at *m/z* 631.1311 (C_29_H_27_O_16_), which was 44 Da (CO_2_) higher than that of metabolites **A38**. MS^2^ spectra gave ions at *m/z* 313.0564 (C_13_H_13_O_9_), 273.0771 (C_15_H_13_O_5_), 255.0666 (C_15_H_11_O_4_), 193.0132 (C_15_H_11_O_4_), 169.0136 (C_7_H_5_O_5_), and 149.0232 (C_8_H_5_O_3_), and all fragment ions were consistent with metabolites **A38**. Therefore, the metabolites **A52-1** and **A52-2** were tentatively characterized to be gallic acid-O-glycoside-O-afzelechin acyl-cooh.

Metabolites **A53-1, A53-2**, and **A53-3** showed same [M-H]^−^ ion at *m/z* 635.0899 (C_27_H_23_O_18_) and MS^2^ spectra gave ions at *m/z* 465.0681(C_20_H_17_O_13_), 313.0558 (C_13_H_13_O_9_), and 169.0136 (C_7_H_5_O_5_). Fragment ion *m/z* 465.0681 was obtained by removing one molecule of gallic acid from the parent ion. Metabolites **A53-1, A53-2**, and **A53-3** were identified to be trigalloyl glucose.

Metabolites **A54-1** to **A54-4** showed the same [M-H]^−^ ion at *m/z* 709.1422 (C_34_H_29_O_17_) and MS^2^ spectra gave ions at *m/z* 557.1300 (C_27_H_25_O_13_), 465.0681(C_20_H_17_O_13_), 405.1185 (C_20_H_21_O_9_), 313.0563 (C_13_H_13_O_9_), and 169.0134 (C_7_H_5_O_5_). The ion *m/z* 557.1300 of C_7_H_4_O_4_ was lost due to gallic acid moiety. Therefore, metabolites **A54-1** to **A54-4** were determined as di-gallic acid-O-glycoside-O-tetrahydroxystilbene acyl and metabolites **A55-1** to **A55-7** were tentatively characterized as gallic acid-O-di-glycoside-O-tetrahydroxystilbene-acyl. The [M-H]^−^ ion at *m/z* 719.1839 (C_33_H_35_O_18_) and MS^2^ spectra gave ions at *m/z* 557.1300 (C_27_H_25_O_13_), 465.0681(C_20_H_17_O_13_), 405.1185 (C_20_H_21_O_9_), 313.0563 (C_13_H_13_O_9_), and 169.0134 (C_7_H_5_O_5_).

Metabolites **A56-1** to **A56-5** showed the same [M-H]^−^ ion at *m/z* 727.1313 (C_37_H_27_O_16_) and MS^2^ spectra gave ions at *m/z* 575.1183 (C_30_H_23_O_12_), 539.0986 (C_30_H_19_O_10_), 449.0883 (C_24_H_17_O_9_), 407.0781 (C_22_H_15_O_8_), 289.0721 (C_15_H_13_O_6_), 285.0407 (C_15_H_9_O_6_), 269.0457 (C_15_H_9_O_5_), 241.0504 (C_14_H_9_O_4_), 169.0134 (C_7_H_5_O_5_), and 125.0231 (C_6_H_5_O_3_). Fragment ions *m/z* 407.0781 and 289.0721 indicated that the metabolites were procyanidins, and fragment ion *m/z* 169.0134 indicated that the metabolites were procyanidins and gallic acid. By comparison with the literature, metabolites **A56-1** to **A56-5** were identified to be procyanidin A-O-galloyl. Similarly, metabolites **A57-1** to **A57-12** were tentatively characterized to be procyanidin B2-O-galloyl. Since the [M-H]^−^ ion at *m/z* 729.1470 (C_37_H_29_O_16_) and MS^2^ spectra gave ions at *m/z* 407.0770 (C_22_H_15_O_8_), 289.0721 (C_15_H_13_O_6_), 169.0134 (C_7_H_5_O_5_), 161.0233 (C_9_H_5_O_3_), 137.0232 (C_7_H_5_O_3_), and 125.0231 (C_6_H_5_O_3_). Similarly, metabolites **A59-1** to **A59-5** were tentatively characterized to be procyanidin-O-galloyl, because of the [M-H]^−^ ion at *m/z* 745.1421 (C_37_H_29_O_17_) and because MS^2^ spectra gave ions at *m/z* 407.0770 (C_22_H_15_O_8_), 289.0721 (C_15_H_13_O_6_), 245.0810 (C_14_H_13_O_4_), 169.0134 (C_7_H_5_O_5_), 177.0183 (C_9_H_5_O_4_), 161.0233 (C_9_H_5_O_3_), 137.0232 (C_7_H_5_O_3_), and 125.0231 (C_6_H_5_O_3_).

Metabolites **A60-1** to **A60-7** showed the same [M-H]^−^ ion at *m/z* 881.1581 (C_44_H_33_O_20_) and MS^2^ spectra gave ions at *m/z* 577.1356 (C_30_H_25_O_12_), 407.0781 (C_22_H_15_O_8_), 289.0721 (C_15_H_13_O_6_), 245.0810 (C_14_H_13_O_4_), 169.0134 (C_7_H_5_O_5_), 161.0233 (C_9_H_5_O_3_), 137.0232 (C_7_H_5_O_3_), and 125.0231 (C_6_H_5_O_3_). Fragment ion *m/z* 577.1356 was obtained from the loss of 304 Da (C_14_H_8_O_8_) of the parent ion, which should be two gallic acid substituents. By comparison with the literature, metabolites **A60-1** to **A60-7** were identified to be procyanidin B2-O-di-galloyl. Similarly, metabolites **A61-1**, **A61-2**, and **A61-3** were tentatively characterized to be epicatechin-O-gallate (4β-8)-(−)-epigallocatechin-O-gallate. Since the [M-H]^−^ ion at *m/z* 897.1533 (C_44_H_33_O_21_) and MS^2^ spectra gave ions at *m/z* 407.0770 (C_22_H_15_O_8_), 289.0721 (C_15_H_13_O_6_), 245.0810 (C_14_H_13_O_4_), 177.0183 (C_9_H_5_O_4_),169.0134 (C_7_H_5_O_5_), 161.0233 (C_9_H_5_O_3_), 137.0232 (C_7_H_5_O_3_), and 125.0231 (C_6_H_5_O_3_).

Metabolites **A62-1** to **A62-10** showed the same [M-H]^−^ ion at *m/z* 1,017.2110 (C_52_H_41_O_22_) and MS^2^ spectra gave ions at *m/z* 677.1306 (C_37_H_25_O_13_), 525.0829 (C_29_H_17_O_10_), 451.1047 (C_24_H_19_O_9_), 407.0770 (C_22_H_15_O_8_), 289.0721 (C_15_H_13_O_6_), 245.0810 (C_14_H_13_O_4_), 177.0183 (C_9_H_5_O_4_), 169.0134 (C_7_H_5_O_5_), 161.0233 (C_9_H_5_O_3_), 137.0232 (C_7_H_5_O_3_), and 125.0231 (C_6_H_5_O_3_). Comparison with the literature revealed metabolites **A62-1** to **A62-10** to be [Epicatechin-(4β->8)]2-epicatechin 3‴-gallate. Similarly, metabolites **A63-1** to **A63-5** were characterized tentatively to be procyanidin C-1 3′, 3″-di-O-gallate. The [M-H]^−^ ion at *m/z* 1,169.2220 (C_59_H_45_O_26_) was 152 Da (C_7_H_4_O_4_) higher than that of metabolites **A62**, MS^2^ spectra gave ions at *m/z* 677.1306 (C_37_H_25_O_13_), 559.1238 (C_30_H_23_O_11_), 525.0829 (C_29_H_17_O_10_), 451.1047 (C_24_H_19_O_9_), 407.0770 (C_22_H_15_O_8_), 289.0721 (C_15_H_13_O_6_), 245.0810 (C_14_H_13_O_4_), 177.0183 (C_9_H_5_O_4_), 169.0134 (C_7_H_5_O_5_), 161.0233 (C_9_H_5_O_3_), 137.0232 (C_7_H_5_O_3_), and 125.0231 (C_6_H_5_O_3_).

In addition, metabolites **A28-1** to **A28-4** displayed a high-resolution [M-H]^−^ ion at *m/z* 547.1464, gave an element composition of C_26_H_27_O_13_, and product ions at *m/z* 529.1357 (C_26_H_25_O_12_), 503.1205 (C_24_H_23_O_12_), 313.0563 (C_13_H_13_O_9_), 233.0815 (C_13_H_13_O_4_), 169.0134 (C_7_H_5_O_5_), and 151.0024 (C_7_H_3_O_4_). Metabolites **A44-1** to **A44-4** gave a [M-H]^−^ ion at *m/z* 605.0936 (C_30_H_21_O_14_) and product ions at *m/z* 605.0936 (C_30_H_21_O_14_), 431.0986 (C_20_H_19_O_10_), 413.0874 (C_21_H_17_O_9_), 313.0564 (C_13_H_13_O_9_), 291.0276 (C_17_H_7_O_5_), 269.0457 (C_15_H_9_O_5_), and 169.0134 (C_7_H_5_O_5_), \. Among them, ions at *m/z* 431.0986 and 269.0457 indicated that they should be emodin glucoside, ions at *m/z* 605.0936, 313.0564, and 169.0134 indicated that gallic-acid glucoside was linked to the ion at *m/z* 291.0276. Similarly, metabolites **A46-1** to **A46-4** gave a [M-H]^−^ ion at *m/z* 609.1233 (C_30_H_25_O_14_) and product ions at *m/z* 609.1221 (C_30_H_21_O_14_), 461.0709 (C_21_H_17_O_12_), 417.1190 (C_21_H_21_O_9_), 313.0564 (C_13_H_13_O_9_), 295.0587 (C_17_H_11_O_5_), 273.0768 (C_15_H_13_O_5_), and 169.0134 (C_7_H_5_O_5_), which were 4-Da higher than metabolites **A44**, so they were attributed to be homologous metabolites.

Metabolites **A51-1** and **A51-2** showed the same [M-H]^−^ ion at *m/z* 629.1522 (C_30_H_29_O_15_) and MS^2^ spectra gave identical ions at *m/z* 315.0872 (C_17_H_15_O_6_), 313.0564 (C_13_H_13_O_9_), 297.0771 (C_17_H_13_O_5_), 271.0977 (C_16_H_15_O_4_), and 169.0136 (C_7_H_5_O_5_). The group of the fragment ion at *m/z* 315.0872 was the substituent of the metabolites, but the structure was not known temporarily. Similarly, metabolites **A58-1** and **A58-2** gave a [M-H]^−^ ion at *m/z* 733.1744 (C_37_H_33_O_16_) and MS^2^ spectra gave ions at *m/z* 419.1135 (C_24_H_19_O_7_), 313.0563 (C_13_H_13_O_9_), and 169.0134 (C_7_H_5_O_5_). These metabolites could not be identified accurately based on fragment ions or the literature, and were classified temporarily as “unknown,” all of which were potential new metabolites.

### 3.3 Fragmentation pathway of catechins and derivatives

To identify the derivatives of catechins in the processing of RM, standards of catechin and epicatechin were first analyzed by MS^2^ using the UPLC-Q-Exactive Plus mass spectrometer under the condition mentioned above.

Catechin (**B2-1**, t_R_ = 5.26 min) and epicatechin (**B2-2**, t_R_ = 8.70 min) had an [M-H]^−^ ion at *m/z* 289.0726 (C_15_H_13_O_6_) with MS^2^ ions at *m/z* 289.0721 (C_15_H_13_O_6_), 271.0620 (C_15_H_11_O_5),_ 245.0810 (C_14_H_13_O_4_), 227.0705 (C_14_H_11_O_3_), 205.0498 (C_11_H_9_O_4_), 203.0706 (C_12_H_11_O_3_), 187.0391 (C_11_H_7_O_3_), 179.0340 (C_9_H_7_O_4_), 165.0183 (C_8_H_5_O_4_), 161.0698 (C_10_H_9_O_2_), 151.0390 (C_8_H_7_O_3_), 137.0232 (C_7_H_5_O_3_), and 125.0231 (C_6_H_5_O_3_). The fragmentation pathway we postulated is shown in Figure 5. Among them, ions at *m/z* 289.0726 and 205.0498 were the identification ions of the monomer of catechin metabolites.

Metabolites **B1-1** and **B1-2** showed the same [M-H]^−^ ion at *m/z* 137.0233 (C_7_H_5_O_3_) and MS^2^ spectra gave ions at *m/z* 137.0233 (C_7_H_5_O_3_). Comparison with the literature suggested metabolites **B1-1** and **B1-2** were protocatechualdehyde.

Metabolites **B3-1**, **B3-2**, and **B3-3** showed the same [M-H]^−^ ion at *m/z* 305.0668 (C_15_H_13_O_7_), 16 Da higher than that of metabolites **B2**. MS^2^ spectra gave ions at *m/z* 305.0665 (C_15_H_13_O_7_), 261.0771 (C_14_H_13_O_5_), 243.0663 (C_14_H_11_O_4_), 221.0450 (C_11_H_9_O_4_), 219.0665 (C_12_H_11_O_4_), 179.0340 (C_9_H_7_O_4_), 165.0183 (C_8_H_5_O_4_), 137.0232 (C_7_H_5_O_3_), and 125.0231 (C_6_H_5_O_3_), which were 16 Da higher than those of metabolites **B2**. Comparison with the literature and the relative t_R_ suggested metabolite **B3-1** to be gallic catechin and metabolites **B3-2** and **B3-3** to be epigallocatechin.

Metabolites **B4-1** and **B4-2** showed the same [M-H]^−^ ion at *m/z* 441.0833 (C_7_H_5_O_3_) and MS^2^ spectra gave ions at *m/z* 289.0721 (C_15_H_13_O_6_), 245.0810 (C_14_H_13_O_4_), 227.0705 (C_14_H_11_O_3_), 205.0498 (C_11_H_9_O_4_), and 169.0134 (C_7_H_5_O_5_). Comparison with the literature suggested that metabolites **B4-1** and **B4-2** were catechin-O-galloyl. Similarly, metabolites **B6-1** and **B6-2** were characterized as gallic catechin-O-galloyl because the [M-H]^−^ ions at *m/z* 457.0781 (C_22_H_17_O_11_) and MS^2^ spectra gave identical ions at *m/z* 305.0668 (C_15_H_13_O_7_) and 169.0134 (C_7_H_5_O_5_).

Metabolites **B5-1** and **B5-2** showed the same [M-H]^−^ ion at *m/z* 451.1249 (C_21_H_23_O_11_) and MS^2^ spectra gave ions at *m/z* 289.0721 (C_15_H_13_O_6_), 245.0810 (C_14_H_13_O_4_), 205.0498 (C_11_H_9_O_4_), and 179.0335 (C_9_H_7_O_4_). The ion *m/z* 289.0721 was derived from the loss of C_6_H_10_O_5_ (hexoside). Thus, metabolites **B5-1** and **B5-2** were identified as catechin-O-glycoside.

Metabolites **B7-1** and **B7-2** gave a [M-H]^−^ ion at *m/z* 561.1405 (C_30_H_25_O_11_) and product ions at *m/z* 407.0770 (C_22_H_15_O_8_), 289.0721 (C_15_H_13_O_6_), 245.0810 (C_14_H_13_O_4_), 205.0497 (C_11_H_9_O_4_), 179.0337 (C_9_H_7_O_4_), 137.0232 (C_7_H_5_O_3_), and 125.0231 (C_6_H_5_O_3_). As described for metabolites **A56**, the characteristic fragment ion was a procyanidin. By comparison with the literature, metabolites **B7-1** and **B7-2** were tentatively characterized to be fisetinidol-(4α,8)-catechin. Similarly, metabolites **B8-1** to **B8-4** were characterized as procyanidin A, because the [M-H]^−^ ion at *m/z* 575.1201 (C_30_H_23_O_12_) and MS^2^ spectra gave ions at *m/z* 575.1201 (C_30_H_23_O_12_), 539.0986 (C_30_H_19_O_10_), 449.0883 (C_24_H_17_O_9_), 407.0770 (C_22_H_15_O_8_), 289.0721 (C_15_H_13_O_6_), 285.0407 (C_15_H_9_O_6_), 241.0504 (C_14_H_9_O_4_), and 125.0231 (C_6_H_5_O_3_). And metabolites **B10-1** to **B10-5** showed the same [M-H]^−^ ion at *m/z* 591.1150 (C_30_H_23_O_13_), 16 Da higher than that of metabolites **B8**. MS^2^ spectra gave ions at *m/z* 555.0932 (C_30_H_19_O_11_), 465.0829 (C_24_H_17_O_10_), 407.0770 (C_22_H_15_O_8_), 327.0509 (C_17_H_11_O_7_), 301.0354 (C_15_H_9_O_7_), 289.0721 (C_15_H_13_O_6_), and 165.0182 (C_8_H_5_O_4_). The ions of 555.0932 and 465.0829 were 16 Da higher than those of metabolites **B2**. Therefore, metabolites **B10-1** to **B10-5** were characterized as procyanidin A-OH.

**FIGURE 6:**
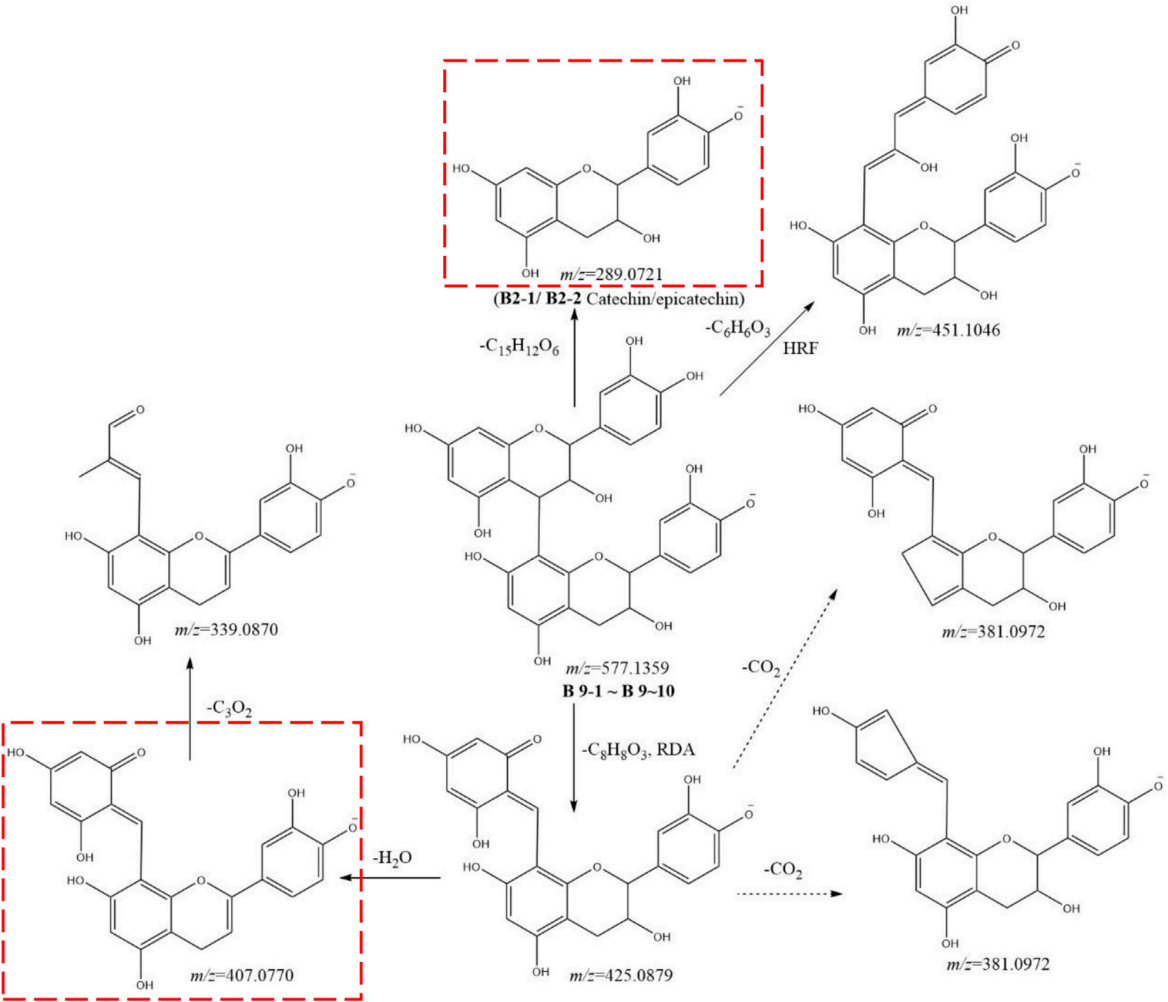
Postulated fragmentation pathways of metabolites B9-1 to B9-10. The ion fragments in the red box are characteristic ion fragments of oligomer catechin metabolites. RDA: retro Diels–Alder reaction; HRF: heterocyclic ring fission.

Metabolites **B9-1** to **B9-10** showed the same [M-H]^−^ ion at *m/z* 557.1359 (C_30_H_25_O_12_) and MS^2^ spectra gave ions at *m/z* 451.1046 (C_24_H_19_O_9_), 425.0879 (C_22_H_17_O_9_), 407.0770 (C_22_H_15_O_8_), 381.0972 (C_21_H_17_O_7_), 339.0870 (C_19_H_15_O_6_), 289.0721 (C_15_H_13_O_6_), 245.0810 (C_14_H_13_O_4_), 225.0500 (C_11_H_9_O_3_), 179.0335 (C_9_H_7_O_4_), 161.0233 (C_9_H_5_O_3_), 137.0232 (C_7_H_5_O_3_), and 125.0231 (C_6_H_5_O_3_). By comparison with the literature, metabolites **B9-1** to **B9-10** were identified as procyanidin B2 (Figure 6) (Qiu, et al., 2013). Similarly, metabolites **B11-1** to **B11-4** were characterized as procyanidin, because of the [M-H]^−^ ion at *m/z* 593.1309 (C_30_H_25_O_13_) and MS^2^ spectra gave ions at *m/z* 407.0770 (C_22_H_15_O_8_), 289.0721 (C_15_H_13_O_6_), 285.0407 (C_15_H_9_O_6_), 245.0810 (C_14_H_13_O_4_), 177.0183 (C_9_H_5_O_4_), 137.0232 (C_7_H_5_O_3_), and 125.0231 (C_6_H_5_O_3_).

Metabolites **B13-1** to **B13-5** showed the same [M-H]^−^ ion at *m/z* 695.1990 (C_35_H_35_O_15_) and MS^2^ spectra gave ions at *m/z* 601.1569 (C_29_H_29_O_14_), 439.1029 (C_23_H_19_O_9_), 393.0973 (C_22_H_17_O_7_), 289.0721 (C_15_H_13_O_6_), and 245.0810 (C_14_H_13_O_4_). By comparison with literature, metabolites **B13-1** to **B13-5** were identified as unknown. Similarly, metabolites **B15-1**, **B15-2**, and **B15-3** were tentatively characterized as metabolites **B13**-OH. Since the [M-H]^−^ ions at *m/z* 711.1940(C_35_H_35_O_16_) and MS^2^ spectra gave ions at *m/z* 407.0770 (C_22_H_15_O_8_), 289.0721 (C_15_H_13_O_6_), 245.0810 (C_14_H_13_O_4_), 177.0183 (C_9_H_5_O_4_),169.0134 (C_7_H_5_O_5_), 161.0233 (C_9_H_5_O_3_), 137.0232 (C_7_H_5_O_3_), and 125.0231 (C_6_H_5_O_3_).

Metabolite **B14** gave a [M-H]-ion at *m/z* 697.1573 (C_37_H_29_O_14_) and MS^2^ spectra gave ions at *m/z* 407.0772 (C_22_H_15_O_8_), 315.0879 (C_17_H_15_O_6_), 289.0721 (C_15_H_13_O_6_), 271.0610 (C_15_H_11_O_5_), 161.0233 (C_9_H_5_O_3_), 137.0232 (C_7_H_5_O_3_), and 125.0231 (C_6_H_5_O_3_). By comparison with literature, metabolite **B14** was tentatively characterized to be procyanidin B2-O-hydroxybenzoyl. Similarly, metabolites **B16-1** and **B16-2** were characterized to be procyanidin B2-O-dihydroxybenzoyl. Since the [M-H]^−^ ion at *m/z* 713.1523 (C_37_H_29_O_15_) was 16 Da (O) higher than that of metabolite **B14**, and MS^2^ spectra gave ions at *m/z* 407.0770 (C_22_H_15_O_8_), 289.0721 (C_15_H_13_O_6_), 271.0610 (C_15_H_11_O_5_), 245.0810 (C_14_H_13_O_4_), 229.0491 (C_13_H_9_O_4_), 137.0232 (C_7_H_5_O_3_), and 125.0231 (C_6_H_5_O_3_).

Metabolites **B20-1** to **B20-7** showed same [M-H]^−^ ion at *m/z* 865.1994 (C_45_H_37_O_18_) and MS^2^ spectra gave identical ions at *m/z* 407.0770 (C_22_H_15_O_8_), 289.0721 (C_15_H_13_O_6_), 245.0825 (C_14_H_13_O_4_), 179.0335 (C_9_H_7_O_4_), 161.0233 (C_9_H_5_O_3_), 137.0232 (C_7_H_5_O_3_), and 125.0231 (C_6_H_5_O_3_), respectively. Comparison with the literature suggested metabolites **B20-1** to **B20-7** to be procyanidin C. Similarly, metabolites **B24-1** to **B24-3** were characterized as cinnamtannin A2 because of the [M-H]^−^ ion at *m/z* 1,153.2628 (C_60_H_49_O_24_) and because MS^2^ spectra gave ions at *m/z* 407.0770 (C_22_H_15_O_8_), 289.0721 (C_15_H_13_O_6_), 287.0568 (C_15_H_11_O_6_), 243.0298 (C_13_H_7_O_5_), and 125.0231 (C_6_H_5_O_3_).

Some metabolites had both catechin and gallic-acid substituents and were identified and analyzed in gallic acid metabolites. These included metabolites **B12-1** to **B12-6** and **A43-1** to **A43-6**, **B17-1** to **B17-5** and **A56-1** to **A56∼5**, **B18-1** to **B18-12** and **A57-1** to **A57-12**, **B19-1** to **B19-5** and **A59-1** to **A59-5**; **B21-1** to **B21-7** and **A60-1** to **60-7**, **B22-1** to **B22-3** and **A61-1** to **A61-3**, **B23-1** to **B23-10** and **A62-1** to **A62-10**, **B25-1** to **B25-6** and **A63-1** to **A63-6**.

### 3.4 Mechanisms of chemical transformation

Several studies have demonstrated that the contents of catechin and epicatechin decrease sharply (and even disappear) after processing, whereas the content of gallic acid increases. Liliang et al. showed that procyanidin was depolymerized into monomeric metabolites, catechin, epicatechin, and epigallocatechin. Then, these monomeric metabolites at high temperatures and high humidity produced protocatechualdehyde and gallic acid. The general metabolic profile was such that our more *nuanced* resolution identified catechins and gallic acid-based metabolites. Of these, 259 metabolites were based on gallic acid, and 112 metabolites were based on catechins. Many metabolites were dehydrated, hydrolyzed, or subjected to more complex reactions during processing. We speculated that procyanidins (including dimers, trimers, and tetramers) generated monomeric metabolites of catechin, epicatechin, gallate catechin, epigallocatechin, and epicatechin due to isomerization during processing at a high temperature and high humidity (Li, et al., 2018). The C-ring, C3-site acyl group of gallate-type metabolites was prone to deglutamylation and hydrolysis reactions.

The phenolic hydroxyl group of the B-ring passes through the “catechin-O-quinone-macromolecular polymer” pathway readily, so oxidation, polymerization, and condensation reactions occur and a yellow-brown oxidation product is generated (Andrew et al., 2008). It is presumed that catechin monomeric metabolites in RM form brown (and even black-brown) products *via* this pathway at a high temperature and high humidity. Also, there is partial decomposition of monomeric metabolites into protocatechualdehyde and gallic acid (Li, et al., 2018). This action also leads to a dramatic decrease in the number of catechins with increasing processing time, which may even be undetectable on equipment with suboptimal detection.

A hydrolysis reaction occurs on the acyl group of gallic acid metabolites. Several metabolites of gallic acid will undergo a hydrolysis reaction. Hence, the content of gallic acid should increase obviously. In reality, the content of gallic acid increases but not very obviously or in a regular fashion. Hence, gallic acid may be consumed by other pathways. Gallic acid may be involved in the Maillard reaction during processing, but so may monosaccharides and amino acids, to generate melanoid metabolites.

The color change during the processing of RM should result from the two pathways: Maillard reaction and browning reaction (the appearance and cross-sectional color are shown in Supplementary Table S2), in which many small-molecule metabolites (e.g., monosaccharides, amino acids, gallic acid, catechins) are consumed.

### 3.5 Trend in content change of gallic acid- and catechin based metabolites

The peak areas of all metabolites were based on the extracted ion chromatographic peaks. Mean values were calculated and the column diagram of each metabolite was drawn, as shown in Supplementary Table S1. In the latter: the black image indicates that the peak area of the metabolites decreases with processing time; the red image indicates that the peak area first increases and then decreases with processing time; the blue image indicates that the peak area increases with processing time; the green image indicates that the change is not significant with processing time. A total of 259 gallic acid metabolites were identified. Of these; 157 gallic acid metabolites had peak areas that decreased with processing time; 71 metabolites had peak areas that increased first and then decreased; 14 metabolites had peak areas that increased with processing time; 17 metabolites had peak areas that did not change significantly during processing. We summed the peak areas of each part. The proportion that decreased with processing time was 48.24%, the proportion that increased with processing time was 36.73%, the proportion that increased first and then decreased was 13.57%, and the proportion that did not change significantly was 1.46%. The content of gallic acid increased with processing time, and the total area of a single metabolite accounted for 35.64%. However, according to the histogram, the increase in the peak area was not very high. In addition to gallic acid, the total area of the other 13 metabolites accounted for 1.09%. This observation bolstered our inference that many gallic acid metabolites lose the galloyl group and form gallic acid during processing, while some gallic acid participates in the Maillard reaction.

**FIGURE 7:**
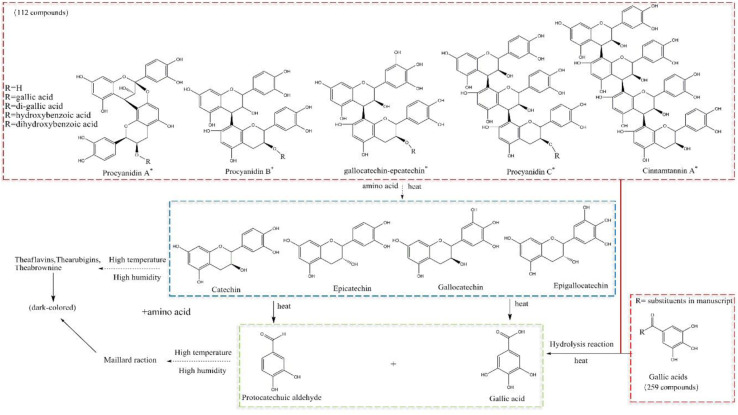
Postulated transformation mechanism of gallic-acid and catechin metabolites during processing, and speculation of surface and section browning.

We identified 112 catechin metabolites. Of these: 81 catechin metabolites had peak areas that decreased with processing time; 30 metabolites had peak areas that increased first and then decreased; one metabolite had a peak area that increased with processing time. We summed the peak areas of each part. The proportion that decreased with processing time was 92.57%, the proportion that increased with processing time was 0.02%, and the proportion that increased first and then decreased was 7.41%. The content of many procyanidins decreased (or even disappeared) during processing, and the content of most of them decreased sharply after 4 h of processing. This process produced catechin, epicatechin, and other metabolites, and the content of these metabolites also decreased gradually. The content of catechin and epicatechin decreased sharply after 4 h of processing and nearly disappeared after 18–24 h of processing. Some studies [14, 17] have shown that catechin and epicatechin break down into protocatechuic aldehyde and gallic acid at a high temperature, but the peak area of protocatechuic aldehyde decreases with processing time. In summary, metabolites such as procyanidins, catechin, and epicatechin are thermally unstable. They break down into many small-molecule metabolites in the early stage of processing, and small-molecule metabolites participate rapidly in the Maillard reaction or other darkening reactions (Figure 7). The literature suggests that procyanidins have antioxidant, anti-inflammatory, immunoregulatory, hepatoprotective, and other effects, but the present study showed that the number of procyanidins decreased (or even disappeared) with processing. The antioxidant effect of RRM is much higher than that of PRM (Gu, et al., 2005; Pu, 2021). Hence, the effects of RM on tonifying the liver and kidneys, nourishing blood essence, and anti-aging are not likely to be related to procyanidins.

## 4 Discussion

In this study, gallic acid and catechin metabolites were identified, and metabolic pathways were analyzed by MS^2^. In the latter, gallicacid metabolites were distinguished by fragment ions, accurate measurement of mass, and fragment pathways of *m/z* 313.0564 and 169.0133. The identification of catechins was relatively complicated. Catechin monomers were identified as diagnostic fragment ions at *m/z* 289.0720 and 245.0816, or diagnostic fragment ions involving oxygenation. Dimers, and trimers at *m/z* 407.0770, 289.0720, and 245.0816. Linked galloyl would also have diagnostic fragment ions from gallic acid. Next, we extracted the ion chromatogram at *m/z* 313.0564 and 289.0720. Then, we set the extraction-ion atlas at ppm <5. Next, we extracted their secondary cracking ions and parent ions one by one to ensure the complete identification of gallic acids and catechin metabolites. Finally, 259 gallic acid and 112 catechin metabolites were identified. The peak areas of 157 gallic acids (accounting for 48.24% of the total area) and 81 catechins (accounting for 92.47% of the total area) decreased gradually. The peak areas of 71 gallic acids (13.57%) and 30 catechins (7.41%) increased first and then decreased. The peak areas of 14 gallic acids (36.73%, of which gallic acid accounted for 35.64%) and one catechin (0.02%) increased gradually, and 17 gallic acids (1.46%) showed no significant change. These data indicated that the processing of RM involved the participation of chemical groups and that the change process was extremely complicated.

We speculate that many gallic-acid metabolites are hydrolyzed to produce gallic acid and that the dimers/trimers of catechins are cleaved into catechins, epicatechin, gallic-acid catechins, and epicatechin monomers, followed by being cleaved into gallic acid and protocatechualdehyde under a high temperature and high humidity and, subsequently, participating in the Maillard reaction and browning reactions, which two reactions deepen the color of RM. Many studies support our speculation (Nonaka et al., 1982; Torres et al., 2002; Wang et al., 2006; li et al., 20,122; Nie, 2017), and similar speculations have also been made in studies related to Polygonum multiflorum (Li et al., 2018). We are conducting a comprehensive identification and analysis of the changes in the binding content of metabolites for speculation.

## 5 Conclusion

This article provides an in-depth identification of the metabolites of gallic acid and catechins, and investigates their transformation during processing. We found that the content of catechins decreased (or even disappeared) after processing, and although the content of gallic acid increased, the amount of gallic acid metabolites also decreased significantly, as reported in literature (Liu et al., 2009; Li et al., 2022). Anthocyanins have antioxidant, anti-inflammatory, immunomodulatory, and hepatoprotective effects. There are also studies on the stronger antioxidant activity of RRM (Liu et al., 2009; Yu et al., 2012), indicating that processing can significantly reduce anthocyanins. Therefore, the role of RM in tonifying the liver and kidney, nourishing blood essence, and anti-aging is not related to anthocyanins. The regularity study of gallic acid and catechins at different processing times in this article can guide the research of processing technology and endpoint. For the study of RM efficacy and toxicity, attention should be paid to the degree of processing of RM and the significant reduction of original catechins, in order to obtain true efficacy and toxicity results.

## Data Availability

The original contributions presented in the study are publicly available. This data can be found here: http://www.gpgenome.com/species/378.
