# Supplementary material for: Processing of Reynoutria multiflora: transformation of catechin and gallic acid derivatives and their identification
Source: Front Pharmacol. 2024 Feb 26;15:1356876. doi: 10.3389/fphar.2024.1356876 (PMC10926517; doi:10.3389/fphar.2024.1356876)
Supplement: Supplementary file 1 [file Table1.DOCX]

Supplementary Table 1

Identification and change curve of the gallic acid compounds in RPM and PPMs by UPLC-Q-Exactive MS

| No. | t_R_(min) | [M-H]^-^ | Error | Formula | Fragment ions | Identification | Change curve |
| --- | --- | --- | --- | --- | --- | --- | --- |
| A1-1 | 1.27 | 169.0133 | -5.600 | C_7_H_5_O_5_ | 169.0133(65%) 125.0232(100%) | gallic acid | 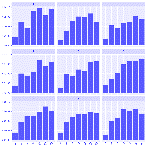 |
| A1-2 | 2.03 | 169.0133 | -5.600 | C_7_H_5_O_5_ | 169.0133(65%) 125.0232(100%) | isomer of gallic acid | 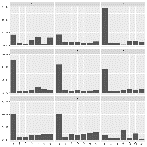 |
| A2-1 | 2.96 | 183.0292 | -3.806 | C_8_H_7_O_5_ | 183.0289(100%) 168.0053(12%) 124.0147(5%) | isomer of Methyl gallate | 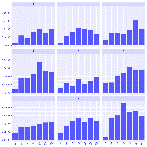 |
| A2-2 | 4.61 | 183.0292 | -3.806 | C_8_H_7_O_5_ | 183.0289(100%) 168.0053(12%) 124.0147(5%) | Methyl gallate | 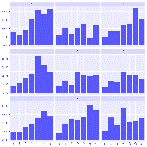 |
| A2-3 | 5.28 | 183.0292 | -3.806 | C_8_H_7_O_5_ | 183.0289(100%) 168.0053(12%) 124.0147(5%) | Methyl gallate | 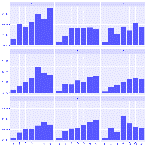 |
| A3-1 | 0.72 | 331.0673 | 0.695 | C_13_H_15_O_10_ | 331.0667(50%) 271.0461(25%) 211.0241(50%) 169.0132(100%) | gallic acid-O-glycoside | 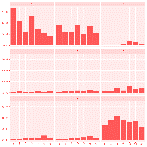 |
| A3-2 | 0.93 | 331.0673 | 0.695 | C_13_H_15_O_10_ | 331.0667(30%) 271.0461(100%) 211.0241(52%) 169.0132(95%) | gallic acid-O-glycoside | 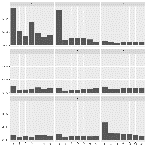 |
| A3-3 | 1.03 | 331.0673 | 0.695 | C_13_H_15_O_10_ | 331.0667(30%) 271.0461(100%) 211.0241(52%) 169.0132(95%) | gallic acid-O-glycoside | 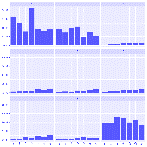 |
| A3-4 | 1.14 | 331.0673 | 0.695 | C_13_H_15_O_10_ | 331.0667(5%) 271.0461(100%) 241.0350(25%) 211.0241(50%) 169.0132(55%) | gallic acid-O-glycoside | 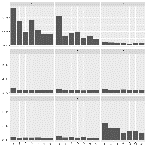 |
| A4-1 | 6.72 | 373.1143 | 0.75 | C_16_H_21_O_10_ | 373.1135(100%) 313.0558(5%) 169.0134(12%) | gallic acid-O-glycoside-O-propanoyl | 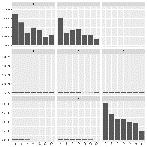 |
| A4-2 | 7.67 | 373.1143 | 0.75 | C_16_H_21_O_10_ | 373.1135(100%) 313.0558(5%) 169.0134(12%) | gallic acid-O-glycoside-O-propanoyl | 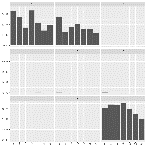 |
| A5-1 | 3.18 | 423.0924 | 0.273 | C_19_H_19_O_12_ | 313.0564(100%) 169.0130(25%) 125.0225(5%) | gallic acid-O-glycoside-O-hydroxyphenyl | 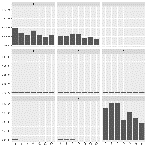 |
| A5-2 | 3.40 | 423.0924 | 0.273 | C_19_H_19_O_12_ | 313.0564(100%) 169.0130(25%) 125.0225(5%) | gallic acid-O-glycoside-O-hydroxyphenyl | 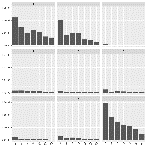 |
| A5-3 | 5.26 | 423.0924 | 0.273 | C_19_H_19_O_12_ | 313.0564(100%) 169.0130(25%) 125.0225(5%) | gallic acid-O-glycoside-O-hydroxyphenyl | 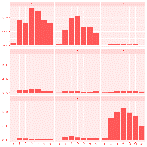 |
| A6 | 10.78 | 437.1090 | 0.15 | C_20_H_21_O_11_ | 313.0565(100%) 169.0134(25%) 151.0023(10%) 123.0432(5%) | gallic acid-O-glycoside-O-methoxyphenoyl | 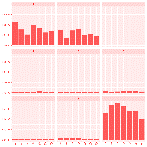 |
| A7-1 | 1.82 | 439.0885 | 0.685 | C_19_H_19_O_12_ | 313.0564(55%) 169.0136(12%) 125.0231(100) | gallic acid-O-glycoside-O-5-hydroxymethylfuryl | 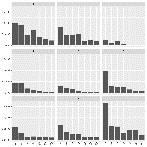 |
| A7-2 | 3.44 | 439.0885 | 0.685 | C_19_H_19_O_12_ | 313.0564(55%) 169.0136(12%) 125.0231(100) | gallic acid-O-glycoside-O-5-hydroxymethylfuryl | 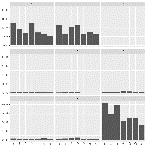 |
| A8-1 | 7.7 | 451.0884 | 0.445 | C_20_H_19_O_12_ | 451.0894(30%) 313.0563(100%) 169.0132(50%) 137.0233(20%) | gallic acid-O-glycoside-O-hydroxybenzoyl | 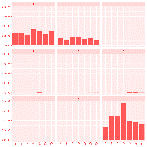 |
| A8-2 | 9.94 | 451.0884 | 0.445 | C_20_H_19_O_12_ | 451.0894(30%) 313.0563(100%) 169.0132(50%) 137.0233(20%) | gallic acid-O-glycoside-O-hydroxybenzoyl | 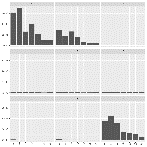 |
| A9-1 | 3.14 | 453.1048 | 0.995 | C_20_H_21_O_12_ | 453.1049(10%) 313.0564(100%) 169.0133(16%) | gallic acid- O-glycoside-O-methoxycatechol acyl | 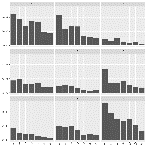 |
| A9-2 | 4.83 | 453.1048 | 0.995 | C_20_H_21_O_12_ | 453.1049(15%) 313.0564(100%) 169.0133(20%) | gallic acid- O-glycoside-O-methoxycatechol acyl | 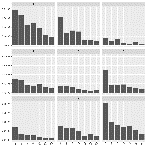 |
| A9-3 | 6.79 | 453.1048 | 0.995 | C_20_H_21_O_12_ | 453.1049(100%) 313.0564(20%) 169.0133(16%) | gallic acid- O-glycoside-O-methoxycatechol acyl | 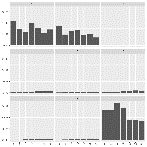 |
| A9-4 | 6.92 | 453.1048 | 0.995 | C_20_H_21_O_12_ | 453.1049(100%) 313.0564(20%) 169.0133(16%) | gallic acid- O-glycoside-O-methoxycatechol acyl | 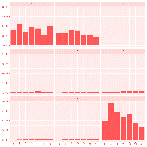 |
| A9-5 | 8.11 | 453.1048 | 0.995 | C_20_H_21_O_12_ | 453.1049(100%) 313.0564(40%) 169.0133(45%) | gallic acid- O-glycoside-O-methoxycatechol acyl | 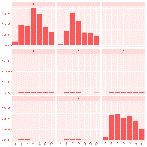 |
| A9-6 | 8.45 | 453.1048 | 0.995 | C_20_H_21_O_12_ | 453.1049(10%) 313.0564(100%) 169.0133(21%) | gallic acid- O-glycoside-O-methoxycatechol acyl | 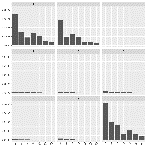 |
| A9-7 | 9.22 | 453.1048 | 0.995 | C_20_H_21_O_12_ | 453.1049(100%) 313.0564(60%) 169.0133(60%) | gallic acid- O-glycoside-O-methoxycatechol acyl | 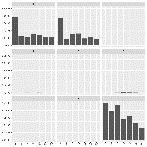 |
| A9-8 | 10 | 453.1048 | 0.995 | C_20_H_21_O_12_ | 453.1049(10%) 313.0564(100%) 169.0133(16%) | gallic acid- O-glycoside-O-methoxycatechol acyl | 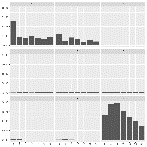 |
| A10-1 | 8.27 | 467.1196 | 0.216 | C_21_H_23_O_12_ | 467.1199(100%) 313.0564(68%) 169.0132(60%) 153.0545(8%) 151.0025(12%) | gallic acid-O-glycoside-O-dimethoxyphenol acyl | 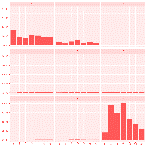 |
| A10-2 | 11.97 | 467.1196 | 0.216 | C_21_H_23_O_12_ | 467.1199(100%) 313.0564(68%) 169.0132(60%) 153.0545(8%) 151.0025(12%) | gallic acid-O-glycoside-O-dimethoxyphenol acyl | 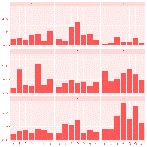 |
| A10-3 | 15.51 | 467.1196 | 0.216 | C_21_H_23_O_12_ | 467.1199(100%) 313.0564(68%) 169.0132(60%) 153.0545(8%) 151.0025(12%) | gallic acid-O-glycoside-O-dimethoxyphenol acyl | 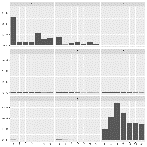 |
| A10-4 | 16.25 | 467.1196 | 0.216 | C_21_H_23_O_12_ | 467.1199(100%) 313.0564(68%) 169.0132(60%) 153.0545(8%) 151.0025(12%) | gallic acid-O-glycoside-O-dimethoxyphenol acyl | 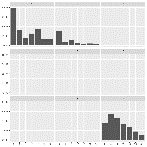 |
| A10-5 | 18.16 | 467.1196 | 0.216 | C_21_H_23_O_12_ | 467.1199(100%) 313.0564(68%) 169.0132(60%) 153.0545(8%) 151.0025(12%) | gallic acid-O-glycoside-O-dimethoxyphenol acyl | 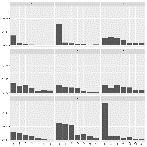 |
| A11-1 | 12.84 | 477.1038 | -0.103 | C_22_H_21_O_12_ | 477.1046(10%) 313.0565(50%) 169.0133(100%) 163.0390(25%) 125.0233(12%) | gallic acid-O-glycoside-O-hydroxycinnamyl | 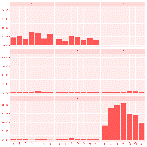 |
| A11-2 | 14.49 | 477.1038 | -0.103 | C_22_H_21_O_12_ | 477.1046(10%) 313.0565(50%) 169.0133(100%) 163.0390(25%) 125.0233(12%) | gallic acid-O-glycoside-O-hydroxycinnamyl | 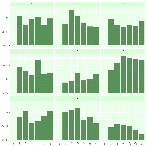 |
| A11-3 | 15.76 | 477.1038 | -0.103 | C_22_H_21_O_12_ | 477.1046(10%) 313.0565(50%) 169.0133(100%) 163.0390(25%) 125.0233(12%) | gallic acid-O-glycoside-O-hydroxycinnamyl | 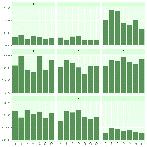 |
| A11-4 | 16.62 | 477.1038 | -0.103 | C_22_H_21_O_12_ | 477.1046(10%) 313.0565(50%) 169.0133(100%) 163.0390(25%) 125.0233(12%) | gallic acid-O-glycoside-O-hydroxycinnamyl | 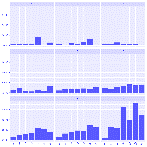 |
| A12-1 | 6.89 | 479.1198 | 0.001 | C_22_H_23_O_12_ | 313.0565(90%) 169.0134(12%) 165.0547(100%) | gallic acid-O-glycoside-O-hydroxyphenyl propionyl | 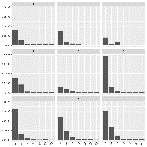 |
| A12-2 | 9.48 | 479.1198 | 0.001 | C_22_H_23_O_12_ | 313.0565(90%) 169.0134(12%) 165.0547(100%) | gallic acid-O-glycoside-O-hydroxyphenyl propionyl | 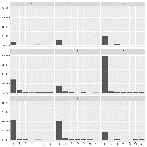 |
| A12-3 | 10.53 | 479.1198 | 0.001 | C_22_H_23_O_12_ | 313.0565(90%) 169.0134(12%) 165.0547(100%) | gallic acid-O-glycoside-O-hydroxyphenyl propionyl | 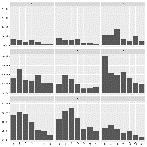 |
| A12-4 | 11.21 | 479.1198 | 0.001 | C_22_H_23_O_12_ | 313.0565(90%) 169.0134(12%) 165.0547(100%) | gallic acid-O-glycoside-O-hydroxyphenyl propionyl | 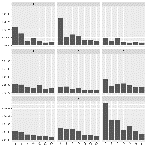 |
| A12-5 | 11.67 | 479.1198 | 0.001 | C_22_H_23_O_12_ | 313.0565(90%) 169.0134(12%) 165.0547(100%) | gallic acid-O-glycoside-O-hydroxyphenyl propionyl | 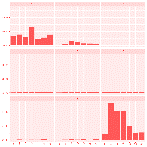 |
| A12-6 | 11.79 | 479.1198 | 0.001 | C_22_H_23_O_12_ | 313.0565(90%) 169.0134(12%) 165.0547(100%) | gallic acid-O-glycoside-O-hydroxyphenyl propionyl | 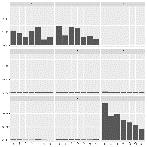 |
| A12-7 | 13.18 | 479.1198 | 0.001 | C_22_H_23_O_12_ | 313.0565(90%) 169.0134(12%) 165.0547(100%) | gallic acid-O-glycoside-O-hydroxyphenyl propionyl | 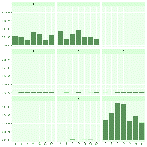 |
| A12-8 | 14.31 | 479.1198 | 0.001 | C_22_H_23_O_12_ | 313.0565(90%) 169.0134(12%) 165.0547(100%) | gallic acid-O-glycoside-O-hydroxyphenyl propionyl | 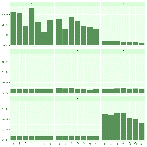 |
| A12-9 | 21.57 | 479.1198 | 0.001 | C_22_H_23_O_12_ | 313.0565(90%) 169.0134(12%) 165.0547(100%) | gallic acid-O-glycoside-O-hydroxyphenyl propionyl | 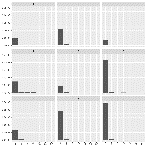 |
| A13-1 | 8.33 | 481.099 | 0.491 | C_21_H_21_O_13_ | 481.0993(30%) 313.0568(100%) 169.0133(55%) | gallic acid-O-glycoside-O-hydroxymethoxybenzoyl | 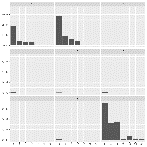 |
| A13-2 | 8.74 | 481.099 | 0.491 | C_21_H_21_O_13_ | 481.0993(30%) 313.0568(100%) 169.0133(55%) | gallic acid-O-glycoside-O-hydroxymethoxybenzoyl | 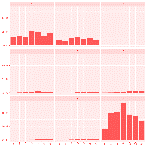 |
| A13-3 | 10.84 | 481.099 | 0.491 | C_21_H_21_O_13_ | 481.0993(30%) 313.0568(100%) 169.0133(55%) | gallic acid-O-glycoside-O-hydroxymethoxybenzoyl | 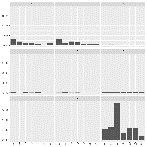 |
| A13-4 | 12.9 | 481.099 | 0.491 | C_21_H_21_O_13_ | 481.0993(30%) 313.0568(100%) 169.0133(55%) | gallic acid-O-glycoside-O-hydroxymethoxybenzoyl | 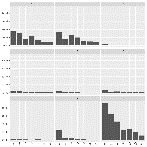 |
| A13-5 | 12.98 | 481.099 | 0.491 | C_21_H_21_O_13_ | 481.0993(30%) 313.0568(100%) 169.0133(55%) | gallic acid-O-glycoside-O-hydroxymethoxybenzoyl | 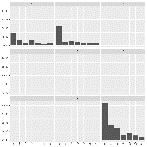 |
| A13-6 | 14.29 | 481.099 | 0.491 | C_21_H_21_O_13_ | 481.0993(30%) 313.0568(100%) 169.0133(55%) | gallic acid-O-glycoside-O-hydroxymethoxybenzoyl | 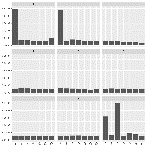 |
| A14-1 | 9.9 | 481.1353 | 0.313 | C_22_H_25_O_12_ | 481.1361(100%) 313.0566(52%) 169.0133(40%) | gallic acid-O-glycoside-O- hydroxymethoxyphenylethanol acyl | 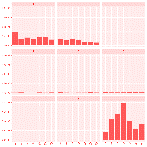 |
| A14-2 | 12.25 | 481.1353 | 0.313 | C_22_H_25_O_12_ | 481.1361(100%) 313.0566(52%) 169.0133(40%) | gallic acid-O-glycoside-O- hydroxymethoxyphenylethanol acyl | 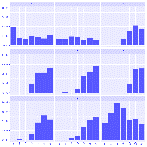 |
| A15-1 | 1.86 | 483.0785 | 0.562 | C_20_H_19_O_14_ | 483.0785(55%) 331.0667(25%) 313.0564(50%) 271.0462(100%) 211.0240(12%) 169.0132(50%) | gallic acid-O-glycoside-O-galloyl | 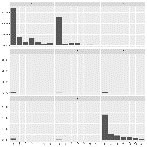 |
| A15-2 | 2.38 | 483.0785 | 0.562 | C_20_H_19_O_14_ | 483.0785(55%) 331.0667(25%) 313.0564(50%) 271.0462(100%) 211.0240(12%) 169.0132(50%) | gallic acid-O-glycoside-O-galloyl | 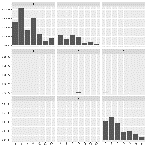 |
| A15-3 | 3.16 | 483.0785 | 0.562 | C_20_H_19_O_14_ | 483.0785(55%) 331.0667(25%) 313.0564(50%) 271.0462(100%) 211.0240(12%) 169.0132(50%) | gallic acid-O-glycoside-O-galloyl | 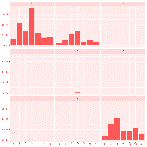 |
| A15-4 | 4.16 | 483.0785 | 0.562 | C_20_H_19_O_14_ | 483.0785(55%) 331.0667(25%) 313.0564(50%) 271.0462(100%) 211.0240(12%) 169.0132(50%) | gallic acid-O-glycoside-O-galloyl | 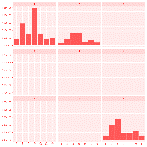 |
| A15-5 | 4.8 | 483.0785 | 0.562 | C_20_H_19_O_14_ | 483.0785(55%) 331.0667(25%) 313.0564(50%) 271.0462(100%) 211.0240(12%) 169.0132(50%) | gallic acid-O-glycoside-O-galloyl | 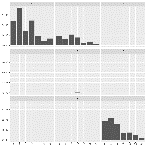 |
| A15-6 | 5.11 | 483.0785 | 0.562 | C_20_H_19_O_14_ | 483.0785(55%) 331.0667(25%) 313.0564(50%) 271.0462(100%) 211.0240(12%) 169.0132(50%) | gallic acid-O-glycoside-O-galloyl | 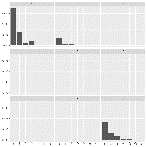 |
| A15-7 | 5.82 | 483.0785 | 0.562 | C_20_H_19_O_14_ | 483.0785(55%) 331.0667(25%) 313.0564(50%) 271.0462(100%) 211.0240(12%) 169.0132(50%) | gallic acid-O-glycoside-O-galloyl | 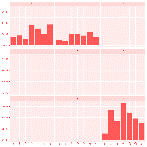 |
| A15-8 | 6 | 483.0785 | 0.562 | C_20_H_19_O_14_ | 483.0785(55%) 331.0667(25%) 313.0564(50%) 271.0462(100%) 211.0240(12%) 169.0132(50%) | gallic acid-O-glycoside-O-galloyl | 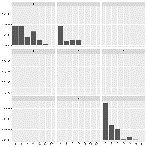 |
| A15-9 | 7.15 | 483.0785 | 0.562 | C_20_H_19_O_14_ | 483.0785(55%) 331.0667(25%) 313.0564(50%) 271.0462(100%) 211.0240(12%) 169.0132(50%) | gallic acid-O-glycoside-O-galloyl | 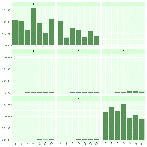 |
| A16-1 | 15.27 | 493.1352 | 0.103 | C_23_H_25_O_12_ | 313.0567(100%) 179.0704(65%) 169.0131(26%) 151.0022(10%) | gallic acid-O-glycoside-O-methoxyphenylpropionyl | 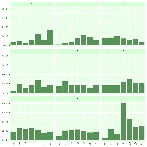 |
| A16-2 | 15.71 | 493.1352 | 0.103 | C_23_H_25_O_12_ | 313.0567(100%) 179.0704(65%) 169.0131(26%) 151.0022(10%) | gallic acid-O-glycoside-O-methoxyphenylpropionyl | 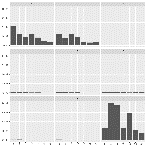 |
| A16-3 | 15.89 | 493.1352 | 0.103 | C_23_H_25_O_12_ | 313.0567(100%) 179.0704(65%) 169.0131(26%) 151.0022(10%) | gallic acid-O-glycoside-O-methoxyphenylpropionyl | 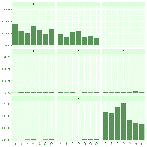 |
| A16-4 | 16.46 | 493.1352 | 0.103 | C_23_H_25_O_12_ | 313.0567(100%) 179.0704(65%) 169.0131(26%) 151.0022(10%) | gallic acid-O-glycoside-O-methoxyphenylpropionyl | 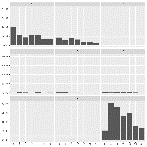 |
| A16-5 | 18.09 | 493.1352 | 0.103 | C_23_H_25_O_12_ | 313.0567(100%) 179.0704(65%) 169.0131(26%) 151.0022(10%) | gallic acid-O-glycoside-O-methoxyphenylpropionyl | 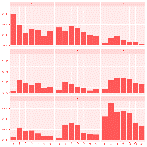 |
| A16-6 | 18.58 | 493.1352 | 0.103 | C_23_H_25_O_12_ | 313.0567(100%) 179.0704(65%) 169.0131(26%) 151.0022(10%) | gallic acid-O-glycoside-O-methoxyphenylpropionyl | 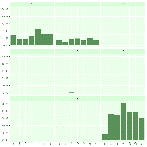 |
| A17-1 | 10.77 | 495.1512 | 0.809 | C_23_H_27_O_12_ | 495.1514(100%) 313.0564(30%) 189.0553(6%) 169.0130(12%) | gallic acid-O-glycoside-O-hydroxy-methoxy-phenylpropanol acyl | 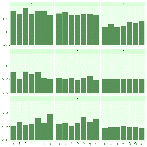 |
| A17-2 | 12.55 | 495.1512 | 0.809 | C_23_H_27_O_12_ | 495.1514(100%) 313.0564(30%) 189.0553(6%) 169.0130(12%) | gallic acid-O-glycoside-O-hydroxy-methoxy-phenylpropanol acyl | 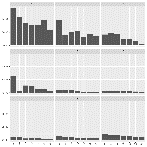 |
| A18 | 14.77 | 497.0941 | 0.848 | C_21_H_21_O_14_ | 345.0823(100%) 313.0563(10%) 183.0291(25%) 169.0127(10%) | gallic acid-O-glycoside-O-methoxyphenoyl | 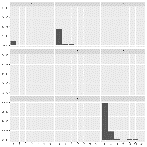 |
| A19-1 | 12.1 | 497.1305 | 0.877 | C_22_H_25_O_13_ | 313.0565(100%) 169.0133(32%) 151.0024(14%) | gallic acid-O-glycoside-O-trimethoxyphenol acyl | 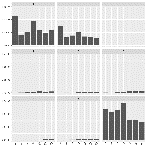 |
| A19-2 | 12.93 | 497.1305 | 0.877 | C_22_H_25_O_13_ | 313.0565(100%) 169.0133(32%) 151.0024(14%) | gallic acid-O-glycoside-O-trimethoxyphenol acyl | 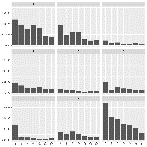 |
| A20-1 | 13.31 | 503.1201 | 1.194 | C_24_H_23_O_12_ | 503.1201(25%) 313.0563(100%) 189.0547(30%) 169.0132(50%) 151.0028(10%) 125.0234(8%) | gallic acid-O-glycoside-O-dimethyl-hydroxycoumarin acyl | 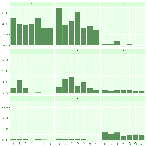 |
| A20-2 | 13.66 | 503.1201 | 1.194 | C_24_H_23_O_12_ | 503.1201(25%) 313.0563(100%) 189.0547(30%) 169.0132(50%) 151.0028(10%) 125.0234(8%) | gallic acid-O-glycoside-O-dimethyl-hydroxycoumarin acyl | 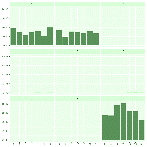 |
| A20-3 | 14.42 | 503.1201 | 1.194 | C_24_H_23_O_12_ | 503.1201(25%) 313.0563(100%) 189.0547(30%) 169.0132(50%) 151.0028(10%) 125.0234(8%) | gallic acid-O-glycoside-O-dimethyl-hydroxycoumarin acyl | 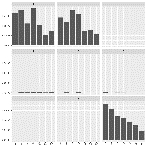 |
| A20-4 | 15.2 | 503.1201 | 1.194 | C_24_H_23_O_12_ | 503.1201(25%) 313.0563(100%) 189.0547(30%) 169.0132(50%) 151.0028(10%) 125.0234(8%) | gallic acid-O-glycoside-O-dimethyl-hydroxycoumarin acyl | 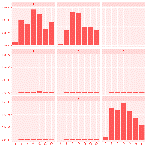 |
| A20-5 | 15.35 | 503.1201 | 1.194 | C_24_H_23_O_12_ | 503.1201(25%) 313.0563(100%) 189.0547(30%) 169.0132(50%) 151.0028(10%) 125.0234(8%) | gallic acid-O-glycoside-O-dimethyl-hydroxycoumarin acyl | 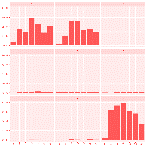 |
| A20-6 | 21.83 | 503.1201 | 1.194 | C_24_H_23_O_12_ | 503.1201(1-%) 313.0563(100%) 189.0547(78%) 169.0132(42%) 151.0028(10%) 125.0234(8%) | gallic acid-O-glycoside-O-dimethyl-hydroxycoumarin acyl | 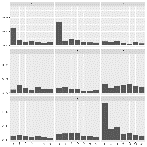 |
| A21-1 | 12.53 | 505.0994 | 1.259 | C_23_H_21_O_13_ | 505.0995(50%) 313.0565(100%) 191.0341(85%) 169.0130(35%) | gallic acid-O-glycoside-O-carboxy-cinnamoyl | 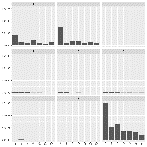 |
| A21-2 | 13.97 | 505.0994 | 1.259 | C_23_H_21_O_13_ | 505.0995(50%) 313.0565(100%) 191.0341(85%) 169.0130(35%) | gallic acid-O-glycoside-O-carboxy-cinnamoyl | 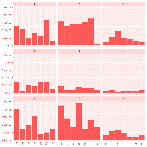 |
| A21-3 | 15.07 | 505.0994 | 1.259 | C_23_H_21_O_13_ | 505.0995(50%) 313.0565(100%) 191.0341(85%) 169.0130(35%) | gallic acid-O-glycoside-O-carboxy-cinnamoyl | 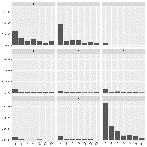 |
| A21-4 | 15.91 | 505.0994 | 1.259 | C_23_H_21_O_13_ | 505.0995(50%) 313.0565(100%) 191.0341(85%) 169.0130(35%) | gallic acid-O-glycoside-O-carboxy-cinnamoyl | 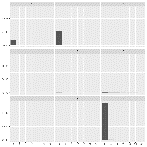 |
| A22-1 | 12.82 | 507.1145 | 0.17 | C_23_H_23_O_13_ | 313.0564(50%) 193.0499(44%) 169.0132(100%) 151.0016(15%) 125.0230(22%) | gallic acid-O-glycoside-O-hydroxy-methoxy-cinnamoyl | 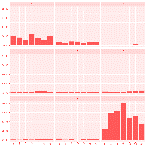 |
| A22-2 | 13.62 | 507.1145 | 0.17 | C_23_H_23_O_13_ | 313.0564(50%) 193.0499(44%) 169.0132(100%) 151.0016(15%) 125.0230(22%) | gallic acid-O-glycoside-O-hydroxy-methoxy-cinnamoyl | 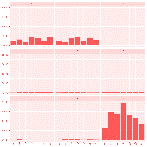 |
| A23-1 | 10.21 | 509.1302 | 0.267 | C_23_H_25_O_13_ | 491.1200(45%) 313.0565(100%) 195.0659(15%) 169.0131(35%) | gallic acid-O-glycoside-O-dimethoxy-phenylacetate acyl | 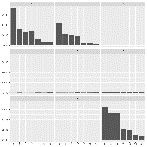 |
| A23-2 | 12.37 | 509.1302 | 0.267 | C_23_H_25_O_13_ | 491.1200(45%) 313.0565(100%) 195.0659(15%) 169.0131(35%) | gallic acid-O-glycoside-O-dimethoxy-phenylacetate acyl | 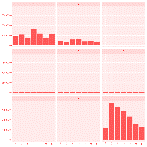 |
| A24 | 5.5 | 511.1095 | 0.336 | C_22_H_23_O_14_ | 511.1095(55%) 467.1206(30%) 313.0564(100%) 169.0134(40%) | gallic acid-O-glycoside-O-hydroxy-dimethoxybenzoyl | 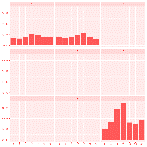 |
| A25-1 | 16.4 | 541.1359 | 1.387 | C_27_H_25_O_12_ | 313.0563(100%) 243.0661(32%) 227.0707(30%) 169.0132(20%) | gallic acid-O-glycoside-O-trihydroxystilbene | 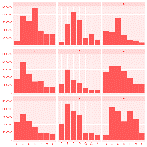 |
| A25-2 | 16.99 | 541.1359 | 1.387 | C_27_H_25_O_12_ | 313.0563(100%) 243.0661(32%) 227.0707(30%) 169.0132(20%) | gallic acid-O-glycoside-O-trihydroxystilbene | 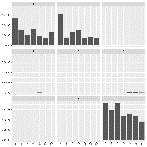 |
| A25-3 | 17.77 | 541.1359 | 1.387 | C_27_H_25_O_12_ | 313.0563(100%) 243.0661(32%) 227.0707(30%) 169.0132(20%) | gallic acid-O-glycoside-O-trihydroxystilbene | 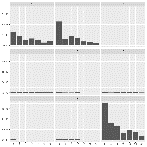 |
| A25-4 | 18.4 | 541.1359 | 1.387 | C_27_H_25_O_12_ | 313.0563(100%) 243.0661(32%) 227.0707(30%) 169.0132(20%) | gallic acid-O-glycoside-O-trihydroxystilbene | 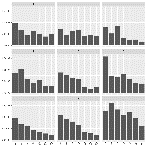 |
| A25-5 | 18.78 | 541.1359 | 1.387 | C_27_H_25_O_12_ | 313.0563(100%) 243.0661(32%) 227.0707(30%) 169.0132(20%) | gallic acid-O-glycoside-O-trihydroxystilbene | 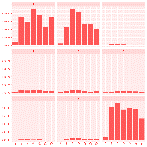 |
| A25-6 | 19.69 | 541.1359 | 1.387 | C_27_H_25_O_12_ | 313.0563(100%) 243.0661(32%) 227.0707(30%) 169.0132(20%) | gallic acid-O-glycoside-O-trihydroxystilbene | 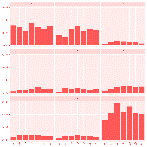 |
| A26-1 | 17.66 | 543.1513 | 0.922 | C_27_H_27_O_12_ | 543.1515(100%) 313.0561(45%) 229.0865(15%) 169.0132(20%) | gallic acid-O-glycoside-O-dihydrotrihydroxystilbene | 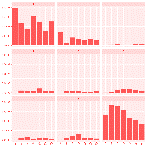 |
| A26-2 | 18.46 | 543.1513 | 0.922 | C_27_H_27_O_12_ | 543.1515(100%) 313.0561(45%) 229.0865(15%) 169.0132(20%) | gallic acid-O-glycoside-O-dihydrotrihydroxystilbene | 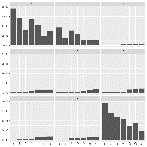 |
| A27-1 | 11.51 | 545.1306 | 0.983 | C_26_H_25_O_13_ | 545.1317(5%) 313.0567(30%) 231.0658(100%) 169.0133(15%) | gallic acid-O-glycoside-O-hydroxymusizin | 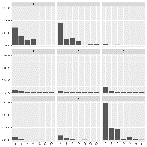 |
| A27-2 | 12.76 | 545.1306 | 0.983 | C_26_H_25_O_13_ | 545.1317(5%) 313.0567(30%) 231.0658(100%) 169.0133(15%) | gallic acid-O-glycoside-O-hydroxymusizin | 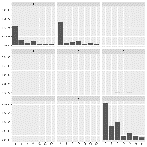 |
| A27-3 | 13.21 | 545.1306 | 0.983 | C_26_H_25_O_13_ | 545.1317(5%) 313.0567(30%) 231.0658(100%) 169.0133(15%) | gallic acid-O-glycoside-O-hydroxymusizin | 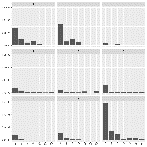 |
| A27-4 | 13.52 | 545.1306 | 0.983 | C_26_H_25_O_13_ | 545.1317(5%) 313.0567(30%) 231.0658(100%) 169.0133(15%) | gallic acid-O-glycoside-O-hydroxymusizin | 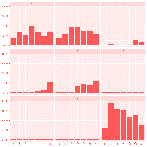 |
| A27-5 | 16.44 | 545.1306 | 0.983 | C_26_H_25_O_13_ | 545.1317(5%) 313.0567(30%) 231.0658(100%) 169.0133(15%) | gallic acid-O-glycoside-O-hydroxymusizin |  |
| A27-6 | 17.28 | 545.1306 | 0.983 | C_26_H_25_O_13_ | 545.1317(5%) 313.0567(30%) 231.0658(100%) 169.0133(15%) | gallic acid-O-glycoside-O-hydroxymusizin |  |
| A27-7 | 19.85 | 545.1306 | 0.983 | C_26_H_25_O_13_ | 545.1317(5%) 313.0567(30%) 231.0658(100%) 169.0133(15%) | gallic acid-O-glycoside-O-hydroxymusizin |  |
| A27-8 | 21.8 | 545.1306 | 0.983 | C_26_H_25_O_13_ | 545.1317(5%) 313.0567(30%) 231.0658(100%) 169.0133(15%) | gallic acid-O-glycoside-O-hydroxymusizin |  |
| A28-1 | 12.82 | 547.1464 | 1.254 | C_26_H_27_O_13_ | 529.1357(25%) 503.1205(25%) 313.0568(100%) 233.0815(12%) 169.0133(30%) 151.0025(5%) 125.0231(5%) | unknown |  |
| A28-2 | 13.11 | 547.1464 | 1.254 | C_26_H_27_O_13_ | 529.1357(25%) 503.1205(25%) 313.0568(100%) 233.0815(12%) 169.0133(30%) 151.0025(5%) 125.0231(5%) | unknown |  |
| A28-3 | 16.72 | 547.1464 | 1.254 | C_26_H_27_O_13_ | 529.1357(25%) 503.1205(25%) 313.0568(100%) 233.0815(12%) 169.0133(30%) 151.0025(5%) 125.0231(5%) | unknown |  |
| A28-4 | 18.69 | 547.1464 | 1.254 | C_26_H_27_O_13_ | 529.1357(25%) 503.1205(25%) 313.0568(100%) 233.0815(12%) 169.0133(30%) 151.0025(5%) 125.0231(5%) | unknown |  |
| A29-1 | 21.97 | 555.1508 | 0.001 | C_28_H_27_O_12_ | 555.1511(100%) 313.0564(40%) 241.0865(12%) 169.0130(15%) | gallic acid-O-glycoside-O-tetrahydroxyphenanthryl |  |
| A29-2 | 23.39 | 555.1508 | 0.001 | C_28_H_27_O_12_ | 555.1511(100%) 313.0564(40%) 241.0865(12%) 169.0130(15%) | gallic acid-O-glycoside-O-tetrahydroxyphenanthryl |  |
| A29-3 | 23.85 | 555.1508 | 0.001 | C_28_H_27_O_12_ | 555.1511(100%) 313.0564(40%) 241.0865(12%) 169.0130(15%) | gallic acid-O-glycoside-O-tetrahydroxyphenanthryl |  |
| A29-4 | 24.53 | 555.1508 | 0.001 | C_28_H_27_O_12_ | 555.1511(100%) 313.0564(40%) 241.0865(12%) 169.0130(15%) | gallic acid-O-glycoside-O-tetrahydroxyphenanthryl |  |
| A29-5 | 24.89 | 555.1508 | 0.001 | C_28_H_27_O_12_ | 555.1511(100%) 313.0564(40%) 241.0865(12%) 169.0130(15%) | gallic acid-O-glycoside-O-tetrahydroxyphenanthryl |  |
| A30-1 | 13.02 | 557.1307 | 1.142 | C_27_H_25_O_13_ | 557.1315(5%) 405.1187(12%) 313.0563(35%) 243.0658(100%) 169.0132(8%) 151.0025(4%) | gallic acid-O-glycoside-O-tetrahydroxystilbene acyl |  |
| A30-2 | 15.51 | 557.1307 | 1.142 | C_27_H_25_O_13_ | 557.1315(5%) 405.1187(12%) 313.0563(35%) 243.0658(100%) 169.0132(8%) 151.0025(4%) | gallic acid-O-glycoside-O-tetrahydroxystilbene acyl |  |
| A30-3 | 15.61 | 557.1307 | 1.142 | C_27_H_25_O_13_ | 557.1315(5%) 405.1187(12%) 313.0563(35%) 243.0658(100%) 169.0132(8%) 151.0025(4%) | gallic acid-O-glycoside-O-tetrahydroxystilbene acyl |  |
| A30-4 | 17.38 | 557.1307 | 1.142 | C_27_H_25_O_13_ | 557.1315(5%) 405.1187(12%) 313.0563(35%) 243.0658(100%) 169.0132(8%) 151.0025(4%) | gallic acid-O-glycoside-O-tetrahydroxystilbene acyl |  |
| A30-5 | 18.8 | 557.1307 | 1.142 | C_27_H_25_O_13_ | 557.1315(5%) 405.1187(12%) 313.0563(35%) 243.0658(100%) 169.0132(8%) 151.0025(4%) | gallic acid-O-glycoside-O-tetrahydroxystilbene acyl |  |
| A30-6 | 19.59 | 557.1307 | 1.142 | C_27_H_25_O_13_ | 557.1315(5%) 405.1187(12%) 313.0563(35%) 243.0658(100%) 169.0132(8%) 151.0025(4%) | gallic acid-O-glycoside-O-tetrahydroxystilbene acyl |  |
| A30-7 | 20.94 | 557.1307 | 1.142 | C_27_H_25_O_13_ | 557.1315(5%) 405.1187(12%) 313.0563(35%) 243.0658(100%) 169.0132(8%) 151.0025(4%) | gallic acid-O-glycoside-O-tetrahydroxystilbene acyl |  |
| A31-1 | 16.02 | 559.146 | 0.511 | C_27_H_27_O_13_ | 559.1458(95%) 407.1342(30%) 313.0562(100%) 245.0817(95%) 169.0132(68%) | gallic acid-O-glycoside-O-dihydrotetrahydroxystilbene acyl |  |
| A31-2 | 25.6 | 559.146 | 0.511 | C_27_H_27_O_13_ | 559.1458(95%) 407.1342(30%) 313.0562(100%) 245.0817(95%) 169.0132(68%) | gallic acid-O-glycoside-O-dihydrotetrahydroxystilbene acyl |  |
| A31-3 | 26.7 | 559.146 | 0.511 | C_27_H_27_O_13_ | 559.1458(95%) 407.1342(30%) 313.0562(100%) 245.0817(95%) 169.0132(68%) | gallic acid-O-glycoside-O-dihydrotetrahydroxystilbene acyl |  |
| A31-4 | 27.66 | 559.146 | 0.511 | C_27_H_27_O_13_ | 559.1458(95%) 407.1342(30%) 313.0562(100%) 245.0817(95%) 169.0132(68%) | gallic acid-O-glycoside-O-dihydrotetrahydroxystilbene acyl |  |
| A32 | 17 | 563.1171 | -3.55 | C_29_H_23_O_12_ | 563.1170(100%) 313.0566(30%) 249.0533(30%) 227.0707(30%) 169.0132(24%) | gallic acid-O-glycoside-O- hydroxy-methyl-anthraquinoyl |  |
| A33 | 16.59 | 569.1309 | 1.469 | C_28_H_25_O_13_ | 313.0562(40%) 255.0663(100%) 169.0131(10%) 151.0018(5%) | gallic acid-O-glycoside-O-emodin anthrone acyl |  |
| A34-1 | 11.67 | 571.1094 | 0.125 | C_27_H_23_O_14_ | 571.1083(65%) 419.0987(/30%) 313.0557(100%) 257,0453(80%) 169.0133(80%) 151.0025(10%) | gallic acid-O-glycoside-O- salicylate diester |  |
| A34-2 | 12.02 | 571.1094 | 0.125 | C_27_H_23_O_14_ | 571.1083(65%) 419.0987(/30%) 313.0557(100%) 257,0453(80%) 169.0133(80%) 151.0025(10%) | gallic acid-O-glycoside-O- salicylate diester |  |
| A34-3 | 15.96 | 571.1094 | 0.125 | C_27_H_23_O_14_ | 571.1083(65%) 419.0987(/30%) 313.0557(100%) 257,0453(80%) 169.0133(80%) 151.0025(10%) | gallic acid-O-glycoside-O- salicylate diester |  |
| A35-1 | 11.09 | 573.1259 | 0.735 | C_27_H_25_O_14_ | 573.1241(45%) 313.0562(40%) 259.0610(100%) 245.0453(20%) 169.0132(55%) | gallic acid-O-glycoside-O-tetrahydroxyphenanthryl |  |
| A35-2 | 12.4 | 573.1259 | 0.735 | C_27_H_25_O_14_ | 573.1241(45%) 313.0562(40%) 259.0610(100%) 245.0453(20%) 169.0132(55%) | gallic acid-O-glycoside-O-tetrahydroxyphenanthryl |  |
| A35-3 | 14.68 | 573.1259 | 0.735 | C_27_H_25_O_14_ | 573.1241(45%) 313.0562(40%) 259.0610(100%) 245.0453(20%) 169.0132(55%) | gallic acid-O-glycoside-O-tetrahydroxyphenanthryl |  |
| A35-4 | 15.09 | 573.1259 | 0.735 | C_27_H_25_O_14_ | 573.1241(45%) 313.0562(40%) 259.0610(100%) 245.0453(20%) 169.0132(55%) | gallic acid-O-glycoside-O-tetrahydroxyphenanthryl |  |
| A35-5 | 20.99 | 573.1259 | 0.735 | C_27_H_25_O_14_ | 573.1241(55%) 531.1160(25%) 313.0562(100%) 259.0615(48%) 245.0453(60%) 169.0132(68%) | gallic acid-O-glycoside-O-tetrahydroxyphenanthryl |  |
| A36-1 | 18.90 | 583.1102 | 1.152 | C_28_H_23_O_14_ | 583.1095(100%) 431.0983(15%) 313.0564(32%) 269.0458(55%) 169.0132(25%) | gallic acid-C-glycoside-O-emodin acyl |  |
| A36-2 | 19.42 | 583.1102 | 1.152 | C_28_H_23_O_14_ | 583.1095(100%) 431.0983(15%) 313.0564(32%) 269.0458(55%) 169.0132(25%) | gallic acid-C-glycoside-O-emodin acyl |  |
| A36-3 | 21.91 | 583.1102 | 1.152 | C_28_H_23_O_14_ | 583.1095(100%) 431.0983(15%) 313.0564(32%) 269.0458(55%) 169.0132(25%) | gallic acid-C-glycoside-O-emodin acyl |  |
| A36-4 | 22.74 | 583.1102 | 1.152 | C_28_H_23_O_14_ | 583.1095(100%) 431.0983(15%) 313.0564(32%) 269.0458(55%) 169.0132(25%) | gallic acid-C-glycoside-O-emodin acyl |  |
| A36-5 | 25.20 | 583.1102 | 1.152 | C_28_H_23_O_14_ | 583.1095(100%) 431.0983(15%) 313.0564(32%) 269.0458(55%) 169.0132(25%) | gallic acid-C-glycoside-O-emodin acyl |  |
| A36-6 | 25.93 | 583.1102 | 1.152 | C_28_H_23_O_14_ | 583.1095(100%) 431.0983(15%) 313.0564(32%) 269.0458(55%) 169.0132(25%) | gallic acid-C-glycoside-O-emodin acyl |  |
| A36-7 | 27.28 | 583.1102 | 1.152 | C_28_H_23_O_14_ | 583.1095(100%) 431.0983(15%) 313.0564(32%) 269.0458(55%) 169.0132(25%) | gallic acid-C-glycoside-O-emodin acyl |  |
| A36-8 | 27.68 | 583.1102 | 1.152 | C_28_H_23_O_14_ | 583.1095(100%) 431.0983(15%) 313.0564(32%) 269.0458(55%) 169.0132(25%) | gallic acid-C-glycoside-O-emodin acyl |  |
| A36-9 | 27.89 | 583.1102 | 1.152 | C_28_H_23_O_14_ | 583.1095(100%) 431.0983(15%) 313.0564(32%) 269.0458(55%) 169.0132(25%) | gallic acid-C-glycoside-O-emodin acyl |  |
| A37-1 | 14.92 | 585.1248 | 1.404 | C_28_H_25_O_14_ | 585.1248(30%) 541.1355(100%) 313.0567(30%) 227.0710(6%) 169.0123(10%) | gallic acid-O-glycoside-O- tetrahydroxystilbene-COOH |  |
| A37-2 | 18.54 | 585.1248 | 1.404 | C_28_H_25_O_14_ | 585.1248(30%) 541.1355(100%) 313.0567(30%) 227.0710(6%) 169.0123(10%) | gallic acid-O-glycoside-O- tetrahydroxystilbene-COOH |  |
| A37-3 | 19.39 | 585.1248 | 1.404 | C_28_H_25_O_14_ | 585.1248(30%) 541.1355(100%) 313.0567(30%) 227.0710(6%) 169.0123(10%) | gallic acid-O-glycoside-O- tetrahydroxystilbene-COOH |  |
| A37-4 | 19.58 | 585.1248 | 1.404 | C_28_H_25_O_14_ | 585.1248(30%) 541.1355(100%) 313.0567(30%) 227.0710(6%) 169.0123(10%) | gallic acid-O-glycoside-O- tetrahydroxystilbene-COOH |  |
| A37-5 | 19.8 | 585.1248 | 1.404 | C_28_H_25_O_14_ | 585.1248(30%) 541.1355(100%) 313.0567(30%) 227.0710(6%) 169.0123(10%) | gallic acid-O-glycoside-O- tetrahydroxystilbene-COOH |  |
| A37-6 | 20.25 | 585.1248 | 1.404 | C_28_H_25_O_14_ | 585.1248(30%) 541.1355(100%) 313.0567(30%) 227.0710(6%) 169.0123(10%) | gallic acid-O-glycoside-O- tetrahydroxystilbene-COOH |  |
| A38-1 | 11.07 | 587.1415 | 1.484 | C_28_H_27_O_14_ | 313.0565(85%) 273.0769(82%) 169.0134(15%) 149.0232(100%) | gallic acid-O-glycoside-O-afzelechin acyl |  |
| A38-2 | 17.89 | 587.1415 | 1.484 | C_28_H_27_O_14_ | 313.0565(85%) 273.0769(82%) 169.0134(15%) 149.0232(100%) | gallic acid-O-glycoside-O-afzelechin acyl |  |
| A38-3 | 18.26 | 587.1415 | 1.484 | C_28_H_27_O_14_ | 313.0565(85%) 273.0769(82%) 169.0134(15%) 149.0232(100%) | gallic acid-O-glycoside-O-afzelechin acyl |  |
| A38-4 | 20.68 | 587.1415 | 1.484 | C_28_H_27_O_14_ | 313.0565(85%) 273.0769(82%) 169.0134(15%) 149.0232(100%) | gallic acid-O-glycoside-O-afzelechin acyl |  |
| A38-5 | 21.60 | 587.1415 | 1.484 | C_28_H_27_O_14_ | 313.0565(85%) 273.0769(82%) 169.0134(15%) 149.0232(100%) | gallic acid-O-glycoside-O-afzelechin acyl |  |
| A38-6 | 23.52 | 587.1415 | 1.484 | C_28_H_27_O_14_ | 313.0565(85%) 273.0769(82%) 169.0134(15%) 149.0232(100%) | gallic acid-O-glycoside-O-afzelechin acyl |  |
| A38-7 | 24.2 | 587.1415 | 1.484 | C_28_H_27_O_14_ | 313.0565(85%) 273.0769(82%) 169.0134(15%) 149.0232(100%) | gallic acid-O-glycoside-O-afzelechin acyl |  |
| A39-1 | 12.01 | 589.1206 | 1.2 | C_27_H_25_O_15_ | 313.0561(90%) 275.0565(65%) 257.0456(45%) 231.0652(38%) 211.0241(30%) 169.0127(40%) 151.0026(100%) 125.0232(68%) | gallic acid-O-glycoside-O- hexahydroxystilbene |  |
| A39-2 | 12.26 | 589.1206 | 1.2 | C_27_H_25_O_15_ | 313.0561(90%) 275.0565(65%) 257.0456(45%) 231.0652(38%) 211.0241(30%) 169.0127(40%) 151.0026(100%) 125.0232(68%) | gallic acid-O-glycoside-O- hexahydroxystilbene |  |
| A39-3 | 12.88 | 589.1206 | 1.2 | C_27_H_25_O_15_ | 313.0561(90%) 275.0565(65%) 257.0456(45%) 231.0652(38%) 211.0241(30%) 169.0127(40%) 151.0026(100%) 125.0232(68%) | gallic acid-O-glycoside-O- hexahydroxystilbene |  |
| A39-4 | 14.49 | 589.1206 | 1.2 | C_27_H_25_O_15_ | 313.0561(90%) 275.0565(65%) 257.0456(45%) 231.0652(38%) 211.0241(30%) 169.0127(40%) 151.0026(100%) 125.0232(68%) | gallic acid-O-glycoside-O- hexahydroxystilbene |  |
| A40-1 | 26.4 | 597.1258 | 1.376 | C_29_H_25_O_14_ | 597.1248(100%) 313.0564(89%) 283.0612(22%) 269.0457(40%) 169.0131(100%) | gallic acid-O-glycoside-O-physcion |  |
| A40-2 | 27.31 | 597.1258 | 1.376 | C_29_H_25_O_14_ | 597.1248(100%) 313.0564(89%) 283.0612(22%) 269.0457(40%) 169.0131(100%) | gallic acid-O-glycoside-O-physcion |  |
| A40-3 | 27.89 | 597.1258 | 1.376 | C_29_H_25_O_14_ | 597.1248(100%) 313.0564(89%) 283.0612(22%) 269.0457(40%) 169.0131(100%) | gallic acid-O-glycoside-O-physcion |  |
| A40-4 | 28.67 | 597.1258 | 1.376 | C_29_H_25_O_14_ | 597.1248(100%) 313.0564(89%) 283.0612(22%) 269.0457(40%) 169.0131(100%) | gallic acid-O-glycoside-O-physcion |  |
| A41-1 | 17.82 | 599.1049 | 1.096 | C_28_H_23_O_15_ | 599.1048(100%) 313.0568(15%) 285.0405(22%) 169.0131(5%) | gallic acid-O-glycoside-O-hydroxyemodin |  |
| A41-2 | 19.62 | 599.1049 | 1.096 | C_28_H_23_O_15_ | 599.1048(100%) 313.0568(15%) 285.0405(22%) 169.0131(5%) | gallic acid-O-glycoside-O-hydroxyemodin |  |
| A42-1 | 8.26 | 601.1205 | 1.01 | C_28_H_25_O_15_ | 601.1202(30%) 583.1093(55%) 573.1247(20%) 466.0758(25%) 439.0884(45%) 313.0563(100%) 287.056(40%) 269.0456(75%) 259.0613( 35%) 169.0132(35%) 125.0231(12%) | eriodictyol 7-O-(6''-O-galloyl)-β-D-glucopyranoside |  |
| A42-2 | 13.21 | 601.1205 | 1.01 | C_28_H_25_O_15_ | 601.1202(30%) 583.1093(55%) 573.1247(20%) 466.0758(25%) 439.0884(45%) 313.0563(100%) 287.056(40%) 269.0456(75%) 259.0613( 35%) 169.0132(35%) 125.0231(12%) | eriodictyol 7-O-(6''-O-galloyl)-β-D-glucopyranoside |  |
| A43-1 | 5.45 | 603.1364 | 1.421 | C_28_H_27_O_15_ | 603.1349(10%) 313.0563(45%) 289.0718(100%) 245.0816(26%) 169.0131(10%) 137.0231(15%) | gallic acid-O-glycoside-O-catechin |  |
| A43-2 | 8.82 | 603.1364 | 1.421 | C_28_H_27_O_15_ | 603.1349(10%) 313.0563(45%) 289.0718(100%) 245.0816(26%) 169.0131(10%) 137.0231(15%) | gallic acid-O-glycoside-O-catechin |  |
| A43-3 | 9.33 | 603.1364 | 1.421 | C_28_H_27_O_15_ | 603.1349(10%) 313.0563(45%) 289.0718(100%) 245.0816(26%) 169.0131(10%) 137.0231(15%) | gallic acid-O-glycoside-O-catechin |  |
| A43-4 | 10.43 | 603.1364 | 1.421 | C_28_H_27_O_15_ | 603.1349(10%) 313.0563(45%) 289.0718(100%) 245.0816(26%) 169.0131(10%) 137.0231(15%) | gallic acid-O-glycoside-O-epicatechin |  |
| A43-5 | 10.76 | 603.1364 | 1.421 | C_28_H_27_O_15_ | 603.1349(10%) 313.0563(45%) 289.0718(100%) 245.0816(26%) 169.0131(10%) 137.0231(15%) | gallic acid-O-glycoside-O-epicatechin |  |
| A43-6 | 11.32 | 603.1364 | 1.421 | C_28_H_27_O_15_ | 603.1349(10%) 313.0563(45%) 289.0718(100%) 245.0816(26%) 169.0131(10%) 137.0231(15%) | gallic acid-O-glycoside-O-epicatechin |  |
| A44-1^*^ | 18.44 | 605.0936 | -0.013 | C_30_H_21_O_14_ | 605.0936(10%) 431.0986(15%) 413.0874(70%) 313.0564(100%) 291.0276(20%) 269.0457(60%) 169.0123(35%) | unknown |  |
| A44-2^*^ | 21.91 | 605.0936 | -0.013 | C_30_H_21_O_14_ | 605.0936(10%) 431.0986(15%) 413.0874(70%) 313.0564(100%) 291.0276(20%) 269.0457(60%) 169.0123(35%) | unknown |  |
| A44-3^*^ | 22.74 | 605.0936 | -0.013 | C_30_H_21_O_14_ | 605.0936(10%) 431.0986(15%) 413.0874(70%) 313.0564(100%) 291.0276(20%) 269.0457(60%) 169.0123(35%) | unknown |  |
| A44-4^*^ | 25.94 | 605.0936 | -0.013 | C_30_H_21_O_14_ | 605.0936(10%) 431.0986(15%) 413.0874(70%) 313.0564(100%) 291.0276(20%) 269.0457(60%) 169.0123(35%) | unknown |  |
| A45 | 15.01 | 605.1156 | 1.309 | C_27_H_25_O_16_ | 465.0681(100%) 313.0565(98%) 169.0133(86%) 125.0225(12%) | di-gallic acid-O-glucoside-O-benzyl alcohol |  |
| A46-1^*^ | 17.00 | 609.1233 | -2.756 | C_30_H_25_O_14_ | 609.1221(100%) 461.0709(75%) 417.1190(25%) 313.0565(60%) 295.0587(32%) 273.0768(52%) 169.0133(30%) | unknown |  |
| A46-2^*^ | 20.68 | 609.1233 | -2.756 | C_30_H_25_O_14_ | 609.1221(100%) 461.0709(75%) 417.1190(25%) 313.0565(60%) 295.0587(32%) 273.0768(52%) 169.0133(30%) | unknown |  |
| A46-3^*^ | 21.60 | 609.1233 | -2.756 | C_30_H_25_O_14_ | 609.1221(100%) 461.0709(75%) 417.1190(25%) 313.0565(60%) 295.0587(32%) 273.0768(52%) 169.0133(30%) | unknown |  |
| A46-4^*^ | 17.78 | 609.1233 | -2.756 | C_30_H_25_O_14_ | 609.1221(100%) 461.0709(75%) 417.1190(25%) 313.0565(60%) 295.0587(32%) 273.0768(52%) 169.0133(30%) | unknown |  |
| A47-1 | 17.99 | 613.1207 | 1.326 | C_29_H_25_O_15_ | 613.1201(45%) 569.1323(10%) 313.0563(100%) 299.0562(56%) 255.0665(15%) 169.0132(30%) | gallic acid-O-glycoside-O-questinol |  |
| A47-2 | 18.4 | 613.1207 | 1.326 | C_29_H_25_O_15_ | 613.1201(45%) 569.1323(10%) 313.0563(100%) 299.0562(56%) 255.0665(15%) 169.0132(30%) | gallic acid-O-glycoside-O-questinol |  |
| A47-3 | 19.88 | 613.1207 | 1.326 | C_29_H_25_O_15_ | 613.1201(45%) 569.1323(10%) 313.0563(100%) 299.0562(56%) 255.0665(15%) 169.0132(30%) | gallic acid-O-glycoside-O-questinol |  |
| A47-4 | 20.96 | 613.1207 | 1.326 | C_29_H_25_O_15_ | 613.1201(45%) 569.1323(10%) 313.0563(100%) 299.0562(56%) 255.0665(15%) 169.0132(30%) | gallic acid-O-glycoside-O-questinol |  |
| A47-5 | 21.2 | 613.1207 | 1.326 | C_29_H_25_O_15_ | 613.1201(45%) 569.1323(10%) 313.0563(100%) 299.0562(56%) 255.0665(15%) 169.0132(30%) | gallic acid-O-glycoside-O-questinol |  |
| A48 | 11.09 | 617.1157 | 1.446 | C_28_H_25_O_16_ | 599.1043(80%) 465.0682(35%) 439.0882(40%) 313.0563(100%) 303.0513(25%) 285.0410(85%) 169.0127(22%) 125.0231(25%) | Taxillusin |  |
| A49-1 | 15.71 | 619.1075 | -2.953 | C_31_H_23_O_14_ | 313.0565(100%) 305.0427(5%) 271.0462(10%) 169.0132(55%) | gallic acid-O-glycoside-O-trihydroxynaphthacenequinone |  |
| A49-2 | 27.89 | 619.1075 | -2.953 | C_31_H_23_O_14_ | 313.0565(100%) 305.0427(5%) 271.0462(10%) 169.0132(55%) | gallic acid-O-glycoside-O-trihydroxynaphthacenequinone |  |
| A50 | 16.8 | 629.1158 | 1.577 | C_29_H_25_O_16_ | 465.0675(100%) 459.0928(50%) 313.0563(85%) 295.0448(15%) 271.0460(40%) 169.0131(50%) | di-gallic acid-glycoside-coumaric acid |  |
| A51-1 | 17.88 | 629.1522 | 1.6 | C_30_H_29_O_15_ | 315.0872(100%) 313.0565(60%) 297.0771(35%) 271.0977(48%) 191.0339(32%) 169.0131(12%) | unknown |  |
| A51-2 | 19.36 | 629.1522 | 1.6 | C_30_H_29_O_15_ | 315.0872(100%) 313.0565(60%) 297.0771(35%) 271.0977(48%) 191.0339(32%) 169.0131(12%) | unknown |  |
| A52-1 | 12.9 | 631.1311 | 1.018 | C_29_H_27_O_16_ | 313.0562(60%) 273.0771(88%) 255.0666(35%) 193.0132(15%) 169.0132(16%) 149.0232(100%) | gallic acid-O-glycoside-O-afzelechin acyl-COOH |  |
| A52-2 | 13.54 | 631.1311 | 1.018 | C_29_H_27_O_16_ | 313.0562(60%) 273.0771(88%) 255.0666(35%) 193.0132(15%) 169.0132(16%) 149.0232(100%) | gallic acid-O-glycoside-O-afzelechin acyl-COOH |  |
| A53-1 | 8.70 | 635.0899 | 1.438 | C_27_H_23_O_18_ | 465.0679(100%) 313.0566(60%) 169.0132(70%) | 1,3,6-Trigalloyl glucose |  |
| A53-2 | 9.50 | 635.0899 | 1.438 | C_27_H_23_O_18_ | 465.0679(100%) 313.0566(60%) 169.0132(70%) | 1,3,6-Trigalloyl glucose |  |
| A53-3 | 10.51 | 635.0899 | 1.438 | C_27_H_23_O_18_ | 465.0679(100%) 313.0566(60%) 169.0132(70%) | 1,3,6-Trigalloyl glucose |  |
| A54-1 | 18.46 | 709.1422 | 1.66 | C_34_H_29_O_17_ | 557.1300(75%) 465.0676(100%) 405.1185(15%) 313.0563(90%) 243.0659(35%) 169.0131(57%) | di-gallic acid-O-glycoside-O-tetrahydroxystilbene acyl |  |
| A54-2 | 18.78 | 709.1422 | 1.66 | C_34_H_29_O_17_ | 557.1300(75%) 465.0676(100%) 405.1185(15%) 313.0563(90%) 243.0659(35%) 169.0131(57%) | di-gallic acid-O-glycoside-O-tetrahydroxystilbene acyl |  |
| A54-3 | 21.1 | 709.1422 | 1.66 | C_34_H_29_O_17_ | 557.1300(75%) 465.0676(100%) 405.1185(15%) 313.0563(90%) 243.0659(35%) 169.0131(57%) | di-gallic acid-O-glycoside-O-tetrahydroxystilbene acyl |  |
| A54-4 | 22.38 | 709.1422 | 1.66 | C_34_H_29_O_17_ | 557.1300(75%) 465.0676(100%) 405.1185(15%) 313.0563(90%) 243.0659(35%) 169.0131(57%) | di-gallic acid-O-glycoside-O-tetrahydroxystilbene acyl |  |
| A55-1 | 12.55 | 719.1839 | 1.408 | C_33_H_35_O_18_ | 719.1804(15%) 557.1302(100%) 405.1196(10%) 313.0562(20%) 243.0659(15%) 169.0128(5%) | gallic acid-O-di-glycoside-O-tetrahydroxystilbene acyl |  |
| A55-2 | 14.05 | 719.1839 | 1.408 | C_33_H_35_O_18_ | 719.1804(15%) 557.1302(100%) 405.1196(10%) 313.0562(20%) 243.0659(15%) 169.0128(5%) | gallic acid-O-di-glycoside-O-tetrahydroxystilbene acyl |  |
| A55-3 | 14.41 | 719.1839 | 1.408 | C_33_H_35_O_18_ | 719.1804(15%) 557.1302(100%) 405.1196(10%) 313.0562(20%) 243.0659(15%) 169.0128(5%) | gallic acid-O-di-glycoside-O-tetrahydroxystilbene acyl |  |
| A55-4 | 14.67 | 719.1839 | 1.408 | C_33_H_35_O_18_ | 719.1804(15%) 557.1302(100%) 405.1196(10%) 313.0562(20%) 243.0659(15%) 169.0128(5%) | gallic acid-O-di-glycoside-O-tetrahydroxystilbene acyl |  |
| A55-5 | 15.15 | 719.1839 | 1.408 | C_33_H_35_O_18_ | 719.1804(15%) 557.1302(100%) 405.1196(10%) 313.0562(20%) 243.0659(15%) 169.0128(5%) | gallic acid-O-di-glycoside-O-tetrahydroxystilbene acyl |  |
| A55-6 | 15.38 | 719.1839 | 1.408 | C_33_H_35_O_18_ | 719.1804(15%) 557.1302(100%) 405.1196(10%) 313.0562(20%) 243.0659(15%) 169.0128(5%) | gallic acid-O-di-glycoside-O-tetrahydroxystilbene acyl |  |
| A55-7 | 16.44 | 719.1839 | 1.408 | C_33_H_35_O_18_ | 719.1804(15%) 557.1302(100%) 405.1196(10%) 313.0562(20%) 243.0659(15%) 169.0128(5%) | gallic acid-O-di-glycoside-O-tetrahydroxystilbene acyl |  |
| A56-1 | 8.16 | 727.1313 | 1.158 | C_37_H_27_O_16_ | 575.1183(15%) 539.0986(80%) 449.0883(25%) 407.0781(25%) 289.0721(38%) 285.0407(100%) 269.0457(28%) 241.0504(/19%) 169.0133(25%) 125.0231(22%) | procyanidin A-O-galloyl |  |
| A56-2 | 11.09 | 727.1313 | 1.158 | C_37_H_27_O_16_ | 575.1183(15%) 539.0986(80%) 449.0883(25%) 407.0781(25%) 289.0721(38%) 285.0407(100%) 269.0457(28%) 241.0504(/19%) 169.0133(25%) 125.0231(22%) | procyanidin A-O-galloyl |  |
| A56-3 | 17.25 | 727.1313 | 1.158 | C_37_H_27_O_16_ | 575.1183(15%) 539.0986(80%) 449.0883(25%) 407.0781(25%) 289.0721(38%) 285.0407(100%) 269.0457(28%) 241.0504(/19%) 169.0133(25%) 125.0231(22%) | procyanidin A-O-galloyl |  |
| A56-4 | 19.22 | 727.1313 | 1.158 | C_37_H_27_O_16_ | 575.1183(15%) 539.0986(80%) 449.0883(25%) 407.0781(25%) 289.0721(38%) 285.0407(100%) 269.0457(28%) 241.0504(/19%) 169.0133(25%) 125.0231(22%) | procyanidin A-O-galloyl |  |
| A56-5 | 21.29 | 727.1313 | 1.158 | C_37_H_27_O_16_ | 575.1183(15%) 539.0986(80%) 449.0883(25%) 407.0781(25%) 289.0721(38%) 285.0407(100%) 269.0457(28%) 241.0504(/19%) 169.0133(25%) 125.0231(22%) | procyanidin A-O-galloyl |  |
| A57-1 | 9.48 | 729.147 | 1.224 | C_37_H_29_O_16_ | 407.0770(100%) 289.0721(100%) 245.0447(15%) 169.0132(36%) 161.0233(30%) 137.0232(12%) 125.0231(95%) | procyanidin B2-O-galloyl |  |
| A57-2 | 9.9 | 729.147 | 1.224 | C_37_H_29_O_16_ | 407.0770(100%) 289.0721(100%) 245.0447(15%) 169.0132(36%) 161.0233(30%) 137.0232(12%) 125.0231(95%) | procyanidin B2-O-galloyl |  |
| A57-3 | 10.53 | 729.147 | 1.224 | C_37_H_29_O_16_ | 407.0770(100%) 289.0721(100%) 245.0447(15%) 169.0132(36%) 161.0233(30%) 137.0232(12%) 125.0231(95%) | procyanidin B2-O-galloyl |  |
| A57-4 | 11.19 | 729.147 | 1.224 | C_37_H_29_O_16_ | 407.0770(100%) 289.0721(100%) 245.0447(15%) 169.0132(36%) 161.0233(30%) 137.0232(12%) 125.0231(95%) | procyanidin B2-O-galloyl |  |
| A57-5 | 12.62 | 729.147 | 1.224 | C_37_H_29_O_16_ | 407.0770(100%) 289.0721(100%) 245.0447(15%) 169.0132(36%) 161.0233(30%) 137.0232(12%) 125.0231(95%) | procyanidin B2-O-galloyl |  |
| A57-6 | 13.55 | 729.147 | 1.224 | C_37_H_29_O_16_ | 407.0770(100%) 289.0721(100%) 245.0447(15%) 169.0132(36%) 161.0233(30%) 137.0232(12%) 125.0231(95%) | procyanidin B2-O-galloyl |  |
| A57-7 | 14.87 | 729.147 | 1.224 | C_37_H_29_O_16_ | 407.0770(100%) 289.0721(100%) 245.0447(15%) 169.0132(36%) 161.0233(30%) 137.0232(12%) 125.0231(95%) | procyanidin B2-O-galloyl |  |
| A57-8 | 15.27 | 729.147 | 1.224 | C_37_H_29_O_16_ | 407.0770(100%) 289.0721(100%) 245.0447(15%) 169.0132(36%) 161.0233(30%) 137.0232(12%) 125.0231(95%) | procyanidin B2-O-galloyl |  |
| A57-9 | 15.97 | 729.147 | 1.224 | C_37_H_29_O_16_ | 407.0770(100%) 289.0721(100%) 245.0447(15%) 169.0132(36%) 161.0233(30%) 137.0232(12%) 125.0231(95%) | procyanidin B2-O-galloyl |  |
| A57-10 | 17.13 | 729.147 | 1.224 | C_37_H_29_O_16_ | 407.0770(100%) 289.0721(100%) 245.0447(15%) 169.0132(36%) 161.0233(30%) 137.0232(12%) 125.0231(95%) | procyanidin B2-O-galloyl |  |
| A57-11 | 17.63 | 729.147 | 1.224 | C_37_H_29_O_16_ | 407.0770(100%) 289.0721(100%) 245.0447(15%) 169.0132(36%) 161.0233(30%) 137.0232(12%) 125.0231(95%) | procyanidin B2-O-galloyl |  |
| A57-12 | 18.69 | 729.147 | 1.224 | C_37_H_29_O_16_ | 407.0770(100%) 289.0721(100%) 245.0447(15%) 169.0132(36%) 161.0233(30%) 137.0232(12%) 125.0231(95%) | procyanidin B2-O-galloyl |  |
| A58-1 | 26.93 | 733.1783 | 1.271 | C_37_H_33_O_16_ | 733.1744(5%) 419.1135(100%) 313.0562(40%) 169.0132(15%) | unknown |  |
| A58-2 | 27.5 | 733.1783 | 1.271 | C_37_H_33_O_16_ | 733.1744(5%) 419.1135(100%) 313.0562(40%) 169.0132(15%) | unknown |  |
| A59-1 | 4.41 | 745.1421 | 1.446 | C_37_H_29_O_17_ | 407.0767(75%) 289.0720(100%)245.0810(6%) 177.0183(70%) 169.0125(12%) 137.0224(15%) 125.0230(55%) | procyanidin-O-galloyl |  |
| A59-2 | 7.53 | 745.1421 | 1.446 | C_37_H_29_O_17_ | 407.0767(75%) 289.0720(100%)245.0810(6%) 177.0183(70%) 169.0125(12%) 137.0224(15%) 125.0230(55%) | procyanidin-O-galloyl |  |
| A59-3 | 7.92 | 745.1421 | 1.446 | C_37_H_29_O_17_ | 407.0767(75%) 289.0720(100%)245.0810(6%) 177.0183(70%) 169.0125(12%) 137.0224(15%) 125.0230(55%) | procyanidin-O-galloyl |  |
| A59-4 | 8.79 | 745.1421 | 1.446 | C_37_H_29_O_17_ | 407.0767(75%) 289.0720(100%)245.0810(6%) 177.0183(70%) 169.0125(12%) 137.0224(15%) 125.0230(55%) | procyanidin-O-galloyl |  |
| A59-5 | 11.71 | 745.1421 | 1.446 | C_37_H_29_O_17_ | 407.0767(75%) 289.0720(100%)245.0810(6%) 177.0183(70%) 169.0125(12%) 137.0224(15%) 125.0230(55%) | procyanidin-O-galloyl |  |
| A60-1 | 13.85 | 881.1581 | 1.173 | C_44_H_33_O_20_ | 577.1356(10%) 407.0770(100%) 289.0721(65%) 245.0451(12%) 169.0132(26%) 161.0233(30%) 137.0232(12%) 125.0231(57%) | procyanidin B2-O-di-galloyl |  |
| A60-2 | 14.41 | 881.1581 | 1.173 | C_44_H_33_O_20_ | 577.1356(10%) 407.0770(100%) 289.0721(65%) 245.0451(12%) 169.0132(26%) 161.0233(30%) 137.0232(12%) 125.0231(57%) | procyanidin B2-O-di-galloyl |  |
| A60-3 | 16.10 | 881.1581 | 1.173 | C_44_H_33_O_20_ | 577.1356(10%) 407.0770(100%) 289.0721(65%) 245.0451(12%) 169.0132(26%) 161.0233(30%) 137.0232(12%) 125.0231(57%) | procyanidin B2-O-di-galloyl |  |
| A60-4 | 16.54 | 881.1581 | 1.173 | C_44_H_33_O_20_ | 577.1356(10%) 407.0770(100%) 289.0721(65%) 245.0451(12%) 169.0132(26%) 161.0233(30%) 137.0232(12%) 125.0231(57%) | procyanidin B2-O-di-galloyl |  |
| A60-5 | 17.27 | 881.1581 | 1.173 | C_44_H_33_O_20_ | 577.1356(10%) 407.0770(100%) 289.0721(65%) 245.0451(12%) 169.0132(26%) 161.0233(30%) 137.0232(12%) 125.0231(57%) | procyanidin B2-O-di-galloyl |  |
| A60-6 | 18.42 | 881.1581 | 1.173 | C_44_H_33_O_20_ | 577.1356(10%) 407.0770(100%) 289.0721(65%) 245.0451(12%) 169.0132(26%) 161.0233(30%) 137.0232(12%) 125.0231(57%) | procyanidin B2-O-di-galloyl |  |
| A60-7 | 20.29 | 881.1581 | 1.173 | C_44_H_33_O_20_ | 577.1356(10%) 407.0770(100%) 289.0721(65%) 245.0451(12%) 169.0132(26%) 161.0233(30%) 137.0232(12%) 125.0231(57%) | procyanidin B2-O-di-galloyl |  |
| A61-1 | 11.12 | 897.1533 | 1.47 | C_44_H_33_O_21_ | 407.0767(100%) 289.0720(65%)245.0810(26%) 177.0183(40%) 169.0135(28%) 137.0224(15%) 125.0230(38%) | (-)-epicatechin 3-O-gallate (4β-8)-(-)-epigallocatechin 3-O-gallate |  |
| A61-2 | 11.6 | 897.1533 | 1.47 | C_44_H_33_O_21_ | 407.0767(100%) 289.0720(65%)245.0810(26%) 177.0183(40%) 169.0135(28%) 137.0224(15%) 125.0230(38%) | (-)-epicatechin 3-O-gallate (4β-8)-(-)-epigallocatechin 3-O-gallate |  |
| A61-3 | 17.73 | 897.1533 | 1.47 | C_44_H_33_O_21_ | 407.0767(100%) 289.0720(65%)245.0810(26%) 177.0183(40%) 169.0135(28%) 137.0224(15%) 125.0230(38%) | (-)-epicatechin 3-O-gallate (4β-8)-(-)-epigallocatechin 3-O-gallate |  |
| A62-1 | 8.00 | 1017.211 | 1.577 | C_52_H_41_O_22_ | 677.1306(15%) 525.0829(20%) 451.1047(20%) 407.0766(80%) 289.0720(65%) 245.0810(26%) 177.0183(10%) 169.0135(15%) 161.0235(30%) 137.0224(15%) 125.0230(100%) | [Epicatechin-(4β-&gt;8)]2-epicatechin 3'''-gallate |  |
| A62-2 | 8.73 | 1017.211 | 1.577 | C_52_H_41_O_22_ | 677.1306(15%) 525.0829(20%) 451.1047(20%) 407.0766(80%) 289.0720(65%) 245.0810(26%) 177.0183(10%) 169.0135(15%) 161.0235(30%) 137.0224(15%) 125.0230(100%) | [Epicatechin-(4β-&gt;8)]2-epicatechin 3'''-gallate |  |
| A62-3 | 9.62 | 1017.211 | 1.577 | C_52_H_41_O_22_ | 677.1306(15%) 525.0829(20%) 451.1047(20%) 407.0766(80%) 289.0720(65%) 245.0810(26%) 177.0183(10%) 169.0135(15%) 161.0235(30%) 137.0224(15%) 125.0230(100%) | [Epicatechin-(4β-&gt;8)]2-epicatechin 3'''-gallate |  |
| A62-4 | 11.1 | 1017.211 | 1.577 | C_52_H_41_O_22_ | 677.1306(15%) 525.0829(20%) 451.1047(20%) 407.0766(80%) 289.0720(65%) 245.0810(26%) 177.0183(10%) 169.0135(15%) 161.0235(30%) 137.0224(15%) 125.0230(100%) | [Epicatechin-(4β-&gt;8)]2-epicatechin 3'''-gallate |  |
| A62-5 | 11.34 | 1017.211 | 1.577 | C_52_H_41_O_22_ | 677.1306(15%) 525.0829(20%) 451.1047(20%) 407.0766(80%) 289.0720(65%) 245.0810(26%) 177.0183(10%) 169.0135(15%) 161.0235(30%) 137.0224(15%) 125.0230(100%) | [Epicatechin-(4β-&gt;8)]2-epicatechin 3'''-gallate |  |
| A62-6 | 11.67 | 1017.211 | 1.577 | C_52_H_41_O_22_ | 677.1306(15%) 525.0829(20%) 451.1047(20%) 407.0766(80%) 289.0720(65%) 245.0810(26%) 177.0183(10%) 169.0135(15%) 161.0235(30%) 137.0224(15%) 125.0230(100%) | [Epicatechin-(4β-&gt;8)]2-epicatechin 3'''-gallate |  |
| A62-7 | 12.42 | 1017.211 | 1.577 | C_52_H_41_O_22_ | 677.1306(15%) 525.0829(20%) 451.1047(20%) 407.0766(80%) 289.0720(65%) 245.0810(26%) 177.0183(10%) 169.0135(15%) 161.0235(30%) 137.0224(15%) 125.0230(100%) | [Epicatechin-(4β-&gt;8)]2-epicatechin 3'''-gallate |  |
| A62-8 | 14.19 | 1017.211 | 1.577 | C_52_H_41_O_22_ | 677.1306(15%) 525.0829(20%) 451.1047(20%) 407.0766(80%) 289.0720(65%) 245.0810(26%) 177.0183(10%) 169.0135(15%) 161.0235(30%) 137.0224(15%) 125.0230(100%) | [Epicatechin-(4β-&gt;8)]2-epicatechin 3'''-gallate |  |
| A62-9 | 15.12 | 1017.211 | 1.577 | C_52_H_41_O_22_ | 677.1306(15%) 525.0829(20%) 451.1047(20%) 407.0766(80%) 289.0720(65%) 245.0810(26%) 177.0183(10%) 169.0135(15%) 161.0235(30%) 137.0224(15%) 125.0230(100%) | [Epicatechin-(4β-&gt;8)]2-epicatechin 3'''-gallate |  |
| A62-10 | 17.69 | 1017.211 | 1.577 | C_52_H_41_O_22_ | 677.1306(15%) 525.0829(20%) 451.1047(20%) 407.0766(80%) 289.0720(65%) 245.0810(26%) 177.0183(10%) 169.0135(15%) 161.0235(30%) 137.0224(15%) 125.0230(100%) | [Epicatechin-(4β-&gt;8)]2-epicatechin 3'''-gallate |  |
| A63-1 | 11.67 | 1169.222 | 0.98 | C_59_H_45_O_26_ | 677.1306(40%) 559.1238(35%) 525.0829(20%) 451.1047(20%) 433.0940(40%) 407.0766(55%) 395.0771(70%) 289.0720(65%) 287.0559(75%) 269.0459(75%) 245.0810(26%) 169.0135(20%) 161.0235(30%) 137.0224(22%) 125.0230(100%) | procyanidin C-1 3',3''-di-O-gallate |  |
| A63-2 | 13.85 | 1169.222 | 0.98 | C_59_H_45_O_26_ | 677.1306(40%) 559.1238(35%) 525.0829(20%) 451.1047(20%) 433.0940(40%) 407.0766(55%) 395.0771(70%) 289.0720(65%) 287.0559(75%) 269.0459(75%) 245.0810(26%) 169.0135(20%) 161.0235(30%) 137.0224(22%) 125.0230(100%) | procyanidin C-1 3',3''-di-O-gallate |  |
| A63-3 | 15.14 | 1169.222 | 0.98 | C_59_H_45_O_26_ | 677.1306(40%) 559.1238(35%) 525.0829(20%) 451.1047(20%) 433.0940(40%) 407.0766(55%) 395.0771(70%) 289.0720(65%) 287.0559(75%) 269.0459(75%) 245.0810(26%) 169.0135(20%) 161.0235(30%) 137.0224(22%) 125.0230(100%) | procyanidin C-1 3',3''-di-O-gallate |  |
| A63-4 | 16.09 | 1169.222 | 0.98 | C_59_H_45_O_26_ | 677.1306(40%) 559.1238(35%) 525.0829(20%) 451.1047(20%) 433.0940(40%) 407.0766(55%) 395.0771(70%) 289.0720(65%) 287.0559(75%) 269.0459(75%) 245.0810(26%) 169.0135(20%) 161.0235(30%) 137.0224(22%) 125.0230(100%) | procyanidin C-1 3',3''-di-O-gallate |  |
| A63-5 | 17.33 | 1169.222 | 0.98 | C_59_H_45_O_26_ | 677.1306(40%) 559.1238(35%) 525.0829(20%) 451.1047(20%) 433.0940(40%) 407.0766(55%) 395.0771(70%) 289.0720(65%) 287.0559(75%) 269.0459(75%) 245.0810(26%) 169.0135(20%) 161.0235(30%) 137.0224(22%) 125.0230(100%) | procyanidin C-1 3',3''-di-O-gallate |  |

Identification and change curve of the catechin compounds in RRM and PRMs by UPLC-Q-Exactive MS

| No. | t_R_(min) | [M-H]^-^ | Error | Formula | Fragment ions | Identification | Change curve |
| --- | --- | --- | --- | --- | --- | --- | --- |
| B1-1 | 3.86 | 137.0233 | -0.021 | C_7_H_5_O_3_ | 137.0232(100%) | protocatechualdehyde |  |
| B1-2 | 4.26 | 137.0233 | -0.021 | C_7_H_5_O_3_ | 137.0232(100%) | protocatechualdehyde |  |
| B2-1 | 5.26 | 289.0726 | 1.171 | C_15_H_13_O_6_ | 289.0720(100%) 271.0620(10%) 245.0816(90%) 227.0705(10%) 205.0498(40%) 203.0706(45%) 187.0391(15%) 179.0340(30%) 165.0183(15%) 161.0698(13%) 151.0390(20%) 137.0232(20%) 125.0231(30%) | catechin |  |
| B2-2 | 8.7 | 289.0726 | 1.171 | C_15_H_13_O_6_ | 289.0720(100%) 271.0620(10%) 245.0816(90%) 227.0705(10%) 205.0498(40%) 203.0706(45%) 187.0391(15%) 179.0340(30%) 165.0183(15%) 161.0698(13%) 151.0390(20%) 137.0232(20%) 125.0231(30%) | epicatechin |  |
| B3-1 | 2.03 | 305.0668 | 0.407 | C_15_H_13_O_7_ | 305.0665(50%) 261.0771(20%) 243.0663(10%) 221.0450(22%) 219.0655(35%) 179.0340(42%) 167.0339(40%) 165.0183(38%) 139.0388(30%) 137.0231(30%) 125.0231(100%) | gallic catechin |  |
| B3-2 | 4.19 | 305.0668 | 0.407 | C_15_H_13_O_7_ | 305.0665(50%) 261.0771(20%) 243.0663(10%) 221.0450(22%) 219.0655(35%) 179.0340(42%) 167.0339(40%) 165.0183(38%) 139.0388(30%) 137.0231(30%) 125.0231(100%) | epigallocatechin |  |
| B3-3 | 5.71 | 305.0668 | 0.407 | C_15_H_13_O_7_ | 305.0665(50%) 261.0771(20%) 243.0663(10%) 221.0450(22%) 219.0655(35%) 179.0340(42%) 167.0339(40%) 165.0183(38%) 139.0388(30%) 137.0231(30%) 125.0231(100%) | epigallocatechin |  |
| B4-1 | 13.93 | 441.0833 | 1.315 | C_22_H_17_O_10_ | 289.0721(55%) 245.0817(20%) 205.0497(5%) 169.0132(100%) | catechin-O-galloyl |  |
| B4-2 | 14.63 | 441.0833 | 1.315 | C_22_H_17_O_10_ | 289.0721(55%) 245.0817(20%) 205.0497(5%) 169.0132(100%) | catechin-O-galloyl |  |
| B5-1 | 2.85 | 451.1249 | 0.699 | C_21_H_23_O_11_ | 289.0719(100%) 245.0819(42%) 205.0504(15%) 179.0335(10%) 137.0222(10%) | catechin-O-glycoside |  |
| B5-2 | 3.36 | 451.1249 | 0.699 | C_21_H_23_O_11_ | 289.0719(100%) 245.0819(42%) 205.0504(15%) 179.0335(10%) 137.0222(10%) | catechin-O-glycoside |  |
| B6-1 | 9.1 | 457.0781 | 1.018 | C_22_H_17_O_11_ | 305.0662(10%) 169.0132(100%) | gallic catechin-O-galloyl |  |
| B6-2 | 11.7 | 457.0781 | 1.018 | C_22_H_17_O_11_ | 305.0662(10%) 169.0132(100%) | gallic catechin-O-galloyl |  |
| B7-1 | 6.41 | 561.1405 | 0.473 | C_30_H_25_O_11_ | 407.0761(20%) 289.0720(100%) 245.0818(32%) 205.0497(10%) 179.0337(8%) 137.0224(20%) 125.0229(30%) | fisetinidol-(4α,8)-catechin |  |
| B7-2 | 10.13 | 561.1405 | 0.473 | C_30_H_25_O_11_ | 407.0761(20%) 289.0720(100%) 245.0818(32%) 205.0497(10%) 179.0337(8%) 137.0224(20%) 125.0229(30%) | fisetinidol-(4α,8)-catechin |  |
| B8-1 | 12.39 | 575.1201 | 1.044 | C_30_H_23_O_12_ | 575.1216(15%) 539.0986(42%) 449.0883(55%) 407.0781(25%) 289.0721(80%) 285.0407(100%) 241.0504(19%) 125.0231(26%) | procyanidin A |  |
| B8-2 | 14.39 | 575.1201 | 1.044 | C_30_H_23_O_12_ | 575.1216(15%) 539.0986(42%) 449.0883(55%) 407.0781(25%) 289.0721(80%) 285.0407(100%) 241.0504(19%) 125.0231(26%) | procyanidin A |  |
| B8-3 | 17.02 | 575.1201 | 1.044 | C_30_H_23_O_12_ | 575.1216(15%) 539.0986(42%) 449.0883(55%) 407.0781(25%) 289.0721(80%) 285.0407(100%) 241.0504(19%) 125.0231(26%) | procyanidin A |  |
| B8-4 | 18.29 | 575.1201 | 1.044 | C_30_H_23_O_12_ | 575.1216(15%) 539.0986(42%) 449.0883(55%) 407.0781(25%) 289.0721(80%) 285.0407(100%) 241.0504(19%) 125.0231(26%) | procyanidin A |  |
| B9-1 | 3.8 | 577.1359 | 1.301 | C_30_H_25_O_12_ | 451.1046(5%) 425.0879(5%) 407.0770(90%) 381.0972(8%) 339.0870(16%) 289.0721(100%) 245.0825(26%) 225.0500(10%) 179.0341(10%) 161.0233(24%) 137.0232(12%) 125.0231(70%) | procyanidin B2 |  |
| B9-2 | 4.57 | 577.1359 | 1.301 | C_30_H_25_O_12_ | 451.1046(5%) 425.0879(5%) 407.0770(90%) 381.0972(8%) 339.0870(16%) 289.0721(100%) 245.0825(26%) 225.0500(10%) 179.0341(10%) 161.0233(24%) 137.0232(12%) 125.0231(70%) | procyanidin B2 |  |
| B9-3 | 6.82 | 577.1359 | 1.301 | C_30_H_25_O_12_ | 451.1046(5%) 425.0879(5%) 407.0770(90%) 381.0972(8%) 339.0870(16%) 289.0721(100%) 245.0825(26%) 225.0500(10%) 179.0341(10%) 161.0233(24%) 137.0232(12%) 125.0231(70%) | procyanidin B2 |  |
| B9-4 | 7.47 | 577.1359 | 1.301 | C_30_H_25_O_12_ | 451.1046(5%) 425.0879(5%) 407.0770(90%) 381.0972(8%) 339.0870(16%) 289.0721(100%) 245.0825(26%) 225.0500(10%) 179.0341(10%) 161.0233(24%) 137.0232(12%) 125.0231(70%) | procyanidin B2 |  |
| B9-5 | 8.44 | 577.1359 | 1.301 | C_30_H_25_O_12_ | 451.1046(5%) 425.0879(5%) 407.0770(90%) 381.0972(8%) 339.0870(16%) 289.0721(100%) 245.0825(26%) 225.0500(10%) 179.0341(10%) 161.0233(24%) 137.0232(12%) 125.0231(70%) | procyanidin B2 |  |
| B9-6 | 8.77 | 577.1359 | 1.301 | C_30_H_25_O_12_ | 451.1046(5%) 425.0879(5%) 407.0770(90%) 381.0972(8%) 339.0870(16%) 289.0721(100%) 245.0825(26%) 225.0500(10%) 179.0341(10%) 161.0233(24%) 137.0232(12%) 125.0231(70%) | procyanidin B2 |  |
| B9-7 | 10.25 | 577.1359 | 1.301 | C_30_H_25_O_12_ | 451.1046(5%) 425.0879(5%) 407.0770(90%) 381.0972(8%) 339.0870(16%) 289.0721(100%) 245.0825(26%) 225.0500(10%) 179.0341(10%) 161.0233(24%) 137.0232(12%) 125.0231(70%) | procyanidin B2 |  |
| B9-8 | 10.68 | 577.1359 | 1.301 | C_30_H_25_O_12_ | 451.1046(5%) 425.0879(5%) 407.0770(90%) 381.0972(8%) 339.0870(16%) 289.0721(100%) 245.0825(26%) 225.0500(10%) 179.0341(10%) 161.0233(24%) 137.0232(12%) 125.0231(70%) | procyanidin B2 |  |
| B9-9 | 11.29 | 577.1359 | 1.301 | C_30_H_25_O_12_ | 451.1046(5%) 425.0879(5%) 407.0770(90%) 381.0972(8%) 339.0870(16%) 289.0721(100%) 245.0825(26%) 225.0500(10%) 179.0341(10%) 161.0233(24%) 137.0232(12%) 125.0231(70%) | procyanidin B2 |  |
| B9-10 | 14.97 | 577.1359 | 1.301 | C_30_H_25_O_12_ | 451.1046(5%) 425.0879(5%) 407.0770(90%) 381.0972(8%) 339.0870(16%) 289.0721(100%) 245.0825(26%) 225.0500(10%) 179.0341(10%) 161.0233(24%) 137.0232(12%) 125.0231(70%) | procyanidin B2 |  |
| B10-1 | 8.75 | 591.115 | 0.992 | C_30_H_23_O_13_ | 555.0932(35%) 465.0829(50%) 407.0768(50%) 327.0509(30%) 301.0354(100%) 289.0721(60%) 165.0182(35%) | procyanidin A-OH |  |
| B10-2 | 9.53 | 591.115 | 0.992 | C_30_H_23_O_13_ | 555.0932(35%) 465.0829(50%) 407.0768(50%) 327.0509(30%) 301.0354(100%) 289.0721(60%) 165.0182(35%) | procyanidin A-OH |  |
| B10-3 | 10.96 | 591.115 | 0.992 | C_30_H_23_O_13_ | 555.0932(35%) 465.0829(50%) 407.0768(50%) 327.0509(30%) 301.0354(100%) 289.0721(60%) 165.0182(35%) | procyanidin A-OH |  |
| B10-4 | 11.71 | 591.115 | 0.992 | C_30_H_23_O_13_ | 555.0932(35%) 465.0829(50%) 407.0768(50%) 327.0509(30%) 301.0354(100%) 289.0721(60%) 165.0182(35%) | procyanidin A-OH |  |
| B10-5 | 13.98 | 591.115 | 0.992 | C_30_H_23_O_13_ | 555.0932(35%) 465.0829(50%) 407.0768(50%) 327.0509(30%) 301.0354(100%) 289.0721(60%) 165.0182(35%) | procyanidin A-OH |  |
| B11-1 | 1.6 | 593.1309 | 1.41 | C_30_H_25_O_13_ | 407.0767(100%) 289.0720(95%)245.0810(26%) 177.0183(78%) 137.0224(15%) 125.0230(65%) | procyanidin |  |
| B11-2 | 2.04 | 593.1309 | 1.41 | C_30_H_25_O_13_ | 407.0767(100%) 289.0720(95%)245.0810(26%) 177.0183(78%) 137.0224(15%) 125.0230(65%) | procyanidin |  |
| B11-3 | 2.32 | 593.1309 | 1.41 | C_30_H_25_O_13_ | 407.0767(100%) 289.0720(95%)245.0810(26%) 177.0183(78%) 137.0224(15%) 125.0230(65%) | procyanidin |  |
| B11-4 | 3.97 | 593.1309 | 1.41 | C_30_H_25_O_13_ | 407.0767(100%) 289.0720(95%)245.0810(26%) 177.0183(78%) 137.0224(15%) 125.0230(65%) | procyanidin |  |
| B12-1 /A43-1 | 5.45 | 603.1364 | 1.421 | C_28_H_27_O_15_ | 603.1349(10%) 313.0563(45%) 289.0718(100%) 245.0816(26%) 169.0131(10%) 137.0231(15%) | catechin-O-glycoside-O-gallic acid |  |
| B12-2 /A43-2 | 7.43 | 603.1364 | 1.421 | C_28_H_27_O_15_ | 603.1349(10%) 313.0563(45%) 289.0718(100%) 245.0816(26%) 169.0131(10%) 137.0231(15%) | catechin-O-glycoside-O-gallic acid |  |
| B12-3 /A43-3 | 8.33 | 603.1364 | 1.421 | C_28_H_27_O_15_ | 603.1349(10%) 313.0563(45%) 289.0718(100%) 245.0816(26%) 169.0131(10%) 137.0231(15%) | catechin-O-glycoside-O-gallic acid |  |
| B12-4 /A43-4 | 9.31 | 603.1364 | 1.421 | C_28_H_27_O_15_ | 603.1349(10%) 313.0563(45%) 289.0718(100%) 245.0816(26%) 169.0131(10%) 137.0231(15%) | epicatechin-O-glycoside-O-gallic acid |  |
| B12-5 /A43-5 | 10.65 | 603.1364 | 1.421 | C_28_H_27_O_15_ | 603.1349(10%) 313.0563(45%) 289.0718(100%) 245.0816(26%) 169.0131(10%) 137.0231(15%) | epicatechin-O-glycoside-O-gallic acid |  |
| B12-6 /A43-6 | 12.68 | 603.1364 | 1.421 | C_28_H_27_O_15_ | 603.1349(10%) 313.0563(45%) 289.0718(100%) 245.0816(26%) 169.0131(10%) 137.0231(15%) | epicatechin-O-glycoside-O-gallic acid |  |
| B13-1^*^ | 7.9 | 695.199 | 1.232 | C_35_H_35_O_15_ | 601.1569(15%) 439.1029(10%) 393.0973(10%) 289.0719(100%) 245.0820(25%) 243.0659(30%) | unknown |  |
| B13-2^*^ | 8.59 | 695.199 | 1.232 | C_35_H_35_O_15_ | 601.1569(15%) 439.1029(10%) 393.0973(10%) 289.0719(100%) 245.0820(25%) 243.0659(30%) | unknown |  |
| B13-3^*^ | 10.8 | 695.199 | 1.232 | C_35_H_35_O_15_ | 601.1569(15%) 439.1029(10%) 393.0973(10%) 289.0719(100%) 245.0820(25%) 243.0659(30%) | unknown |  |
| B13-4^*^ | 11.29 | 695.199 | 1.232 | C_35_H_35_O_15_ | 601.1569(15%) 439.1029(10%) 393.0973(10%) 289.0719(100%) 245.0820(25%) 243.0659(30%) | unknown |  |
| B13-5^*^ | 13.24 | 695.199 | 1.232 | C_35_H_35_O_15_ | 601.1569(15%) 439.1029(10%) 393.0973(10%) 289.0719(100%) 245.0820(25%) 243.0659(30%) | unknown |  |
| B14 | 16.36 | 697.1573 | 1.465 | C_37_H_29_O_14_ | 407.0772(100%) 315.0879(40%) 289.0720(90%) 271.0610(15%) 161.0232(30%) 137.0222(10%) 125.0231(65%) | procyanidin B2-O-hydroxybenzoyl |  |
| B15-1^*^ | 4.00 | 711.1940 | 1.324 | C_35_H_35_O_16_ | 531.1290(20%) 455.0977(20%) 393.0973(30%) 289.0719(100%) 259.0616(35%) 153.0180(40%) | unknown |  |
| B15-2^*^ | 5.25 | 711.1940 | 1.324 | C_35_H_35_O_16_ | 531.1290(20%) 455.0977(20%) 393.0973(30%) 289.0719(100%) 259.0616(35%) 153.0180(40%) | unknown |  |
| B15-3^*^ | 7.62 | 711.1940 | 1.324 | C_35_H_35_O_16_ | 531.1290(20%) 455.0977(20%) 393.0973(30%) 289.0719(100%) 259.0616(35%) 153.0180(40%) | unknown |  |
| B16-1 | 12.39 | 713.1523 | 1.552 | C_37_H_29_O_15_ | 407.0764(40%) 289.0724(100%) 271.0618(75%) 245.0822(25%) 229.0491(15%) 137.0222(10%) 125.0230(58%) | procyanidin B2-O-dihydroxybenzoyl |  |
| B16-2 | 12.81 | 713.1523 | 1.552 | C_37_H_29_O_15_ | 407.0764(40%) 289.0724(100%) 271.0618(75%) 245.0822(25%) 229.0491(15%) 137.0222(10%) 125.0230(58%) | procyanidin B2-O-dihydroxybenzoyl |  |
| B17-1 /A56-1 | 17.25 | 727.1313 | 1.158 | C_37_H_27_O_16_ | 575.1183(15%) 539.0986(80%) 449.0883(25%) 407.0781(25%) 289.0721(38%) 285.0407(100%) 269.0457(28%) 241.0504(/19%) 169.0133(25%) 125.0231(22%) | procyanidin A-O-gallate |  |
| B17-2 /A56-2 | 19.22 | 727.1313 | 1.158 | C_37_H_27_O_16_ | 575.1183(15%) 539.0986(80%) 449.0883(25%) 407.0781(25%) 289.0721(38%) 285.0407(100%) 269.0457(28%) 241.0504(/19%) 169.0133(25%) 125.0231(22%) | procyanidin A-O-gallate |  |
| B17-3 /A56-3 | 19.99 | 727.1313 | 1.158 | C_37_H_27_O_16_ | 575.1183(15%) 539.0986(80%) 449.0883(25%) 407.0781(25%) 289.0721(38%) 285.0407(100%) 269.0457(28%) 241.0504(/19%) 169.0133(25%) 125.0231(22%) | procyanidin A-O-gallate |  |
| B17-4 /A56-4 | 20.81 | 727.1313 | 1.158 | C_37_H_27_O_16_ | 575.1183(15%) 539.0986(80%) 449.0883(25%) 407.0781(25%) 289.0721(38%) 285.0407(100%) 269.0457(28%) 241.0504(/19%) 169.0133(25%) 125.0231(22%) | procyanidin A-O-gallate |  |
| B17-5 /A56-5 | 21.29 | 727.1313 | 1.158 | C_37_H_27_O_16_ | 575.1183(15%) 539.0986(80%) 449.0883(25%) 407.0781(25%) 289.0721(38%) 285.0407(100%) 269.0457(28%) 241.0504(/19%) 169.0133(25%) 125.0231(22%) | procyanidin A-O-gallate |  |
| B18-1 /A57-1 | 9.48 | 729.147 | 1.224 | C_37_H_29_O_16_ | 407.0770(100%) 289.0721(100%) 245.0447(15%) 169.0132(36%) 161.0233(30%) 137.0232(12%) 125.0231(95%) | procyanidin B2-O-gallate |  |
| B18-2 /A57-2 | 9.9 | 729.147 | 1.224 | C_37_H_29_O_16_ | 407.0770(100%) 289.0721(100%) 245.0447(15%) 169.0132(36%) 161.0233(30%) 137.0232(12%) 125.0231(95%) | procyanidin B2-O-gallate |  |
| B18-3 /A57-3 | 10.53 | 729.147 | 1.224 | C_37_H_29_O_16_ | 407.0770(100%) 289.0721(100%) 245.0447(15%) 169.0132(36%) 161.0233(30%) 137.0232(12%) 125.0231(95%) | procyanidin B2-O-gallate |  |
| B18-4 /A57-4 | 11.19 | 729.147 | 1.224 | C_37_H_29_O_16_ | 407.0770(100%) 289.0721(100%) 245.0447(15%) 169.0132(36%) 161.0233(30%) 137.0232(12%) 125.0231(95%) | procyanidin B2-O-gallate |  |
| B18-5 /A57-5 | 12.62 | 729.147 | 1.224 | C_37_H_29_O_16_ | 407.0770(100%) 289.0721(100%) 245.0447(15%) 169.0132(36%) 161.0233(30%) 137.0232(12%) 125.0231(95%) | procyanidin B2-O-gallate |  |
| B18-6 /A57-6 | 13.55 | 729.147 | 1.224 | C_37_H_29_O_16_ | 407.0770(100%) 289.0721(100%) 245.0447(15%) 169.0132(36%) 161.0233(30%) 137.0232(12%) 125.0231(95%) | procyanidin B2-O-gallate |  |
| B18-7 /A57-7 | 14.87 | 729.147 | 1.224 | C_37_H_29_O_16_ | 407.0770(100%) 289.0721(100%) 245.0447(15%) 169.0132(36%) 161.0233(30%) 137.0232(12%) 125.0231(95%) | procyanidin B2-O-gallate |  |
| B18-8 /A57-8 | 15.27 | 729.147 | 1.224 | C_37_H_29_O_16_ | 407.0770(100%) 289.0721(100%) 245.0447(15%) 169.0132(36%) 161.0233(30%) 137.0232(12%) 125.0231(95%) | procyanidin B2-O-gallate |  |
| B18-9 /A57-9 | 15.97 | 729.147 | 1.224 | C_37_H_29_O_16_ | 407.0770(100%) 289.0721(100%) 245.0447(15%) 169.0132(36%) 161.0233(30%) 137.0232(12%) 125.0231(95%) | procyanidin B2-O-gallate |  |
| B18-10 /A57-10 | 17.13 | 729.147 | 1.224 | C_37_H_29_O_16_ | 407.0770(100%) 289.0721(100%) 245.0447(15%) 169.0132(36%) 161.0233(30%) 137.0232(12%) 125.0231(95%) | procyanidin B2-O-gallate |  |
| B18-11 /A57-11 | 17.63 | 729.147 | 1.224 | C_37_H_29_O_16_ | 407.0770(100%) 289.0721(100%) 245.0447(15%) 169.0132(36%) 161.0233(30%) 137.0232(12%) 125.0231(95%) | procyanidin B2-O-gallate |  |
| B18-12 /A57-12 | 18.69 | 729.147 | 1.224 | C_37_H_29_O_16_ | 407.0770(100%) 289.0721(100%) 245.0447(15%) 169.0132(36%) 161.0233(30%) 137.0232(12%) 125.0231(95%) | procyanidin B2-O-gallate |  |
| B19-1 /A59-1 | 4.41 | 745.1421 | 1.446 | C_37_H_29_O_17_ | 407.0767(75%) 289.0720(100%)245.0810(6%) 231.0306(%) 177.0183(70%) 169.0125(12%) 137.0224(15%) 125.0230(55%) | procyanidin-O-gallate |  |
| B19-2 /A59-2 | 7.53 | 745.1421 | 1.446 | C_37_H_29_O_17_ | 407.0767(75%) 289.0720(100%)245.0810(6%) 231.0306(%) 177.0183(70%) 169.0125(12%) 137.0224(15%) 125.0230(55%) | procyanidin-O-gallate |  |
| B19-3 /A59-3 | 7.92 | 745.1421 | 1.446 | C_37_H_29_O_17_ | 407.0767(75%) 289.0720(100%)245.0810(6%) 231.0306(%) 177.0183(70%) 169.0125(12%) 137.0224(15%) 125.0230(55%) | procyanidin-O-gallate |  |
| B19-4 /A59-4 | 8.79 | 745.1421 | 1.446 | C_37_H_29_O_17_ | 407.0767(75%) 289.0720(100%)245.0810(6%) 231.0306(%) 177.0183(70%) 169.0125(12%) 137.0224(15%) 125.0230(55%) | procyanidin-O-gallate |  |
| B19-5 /A59-5 | 11.71 | 745.1421 | 1.446 | C_37_H_29_O_17_ | 407.0767(75%) 289.0720(100%)245.0810(6%) 231.0306(%) 177.0183(70%) 169.0125(12%) 137.0224(15%) 125.0230(55%) | procyanidin-O-gallate |  |
| B20-1 | 6.09 | 865.1994 | 0.997 | C_45_H_37_O_18_ | 407.0770(70%) 289.0721(70%) 245.0825(20%) 179.0341(10%) 161.0233(30%) 137.0232(12%) 125.0231(100%) | procyanidin C |  |
| B20-2 | 6.43 | 865.1994 | 0.997 | C_45_H_37_O_18_ | 407.0770(70%) 289.0721(70%) 245.0825(20%) 179.0341(10%) 161.0233(30%) 137.0232(12%) 125.0231(100%) | procyanidin C |  |
| B20-3 | 10.1 | 865.1994 | 0.997 | C_45_H_37_O_18_ | 407.0770(70%) 289.0721(70%) 245.0825(20%) 179.0341(10%) 161.0233(30%) 137.0232(12%) 125.0231(100%) | procyanidin C |  |
| B20-4 | 10.28 | 865.1994 | 0.997 | C_45_H_37_O_18_ | 407.0770(70%) 289.0721(70%) 245.0825(20%) 179.0341(10%) 161.0233(30%) 137.0232(12%) 125.0231(100%) | procyanidin C |  |
| B20-5 | 10.58 | 865.1994 | 0.997 | C_45_H_37_O_18_ | 407.0770(70%) 289.0721(70%) 245.0825(20%) 179.0341(10%) 161.0233(30%) 137.0232(12%) 125.0231(100%) | procyanidin C |  |
| B20-6 | 11.96 | 865.1994 | 0.997 | C_45_H_37_O_18_ | 407.0770(70%) 289.0721(70%) 245.0825(20%) 179.0341(10%) 161.0233(30%) 137.0232(12%) 125.0231(100%) | procyanidin C |  |
| B20-7 | 12.29 | 865.1994 | 0.997 | C_45_H_37_O_18_ | 407.0770(70%) 289.0721(70%) 245.0825(20%) 179.0341(10%) 161.0233(30%) 137.0232(12%) 125.0231(100%) | procyanidin C |  |
| B21-1/A60-1 | 13.85 | 881.1581 | 1.173 | C_44_H_33_O_20_ | 577.1356(10%) 559.1268(20%) 541.1158(10%) 407.0770(100%) 289.0721(65%) 245.0451(12%) 169.0132(26%) 161.0233(30%) 137.0232(12%) 125.0231(57%) | Procyanidin B2-di-O-gallate |  |
| B21-2/A60-2 | 14.41 | 881.1581 | 1.173 | C_44_H_33_O_20_ | 577.1356(10%) 559.1268(20%) 541.1158(10%) 407.0770(100%) 289.0721(65%) 245.0451(12%) 169.0132(26%) 161.0233(30%) 137.0232(12%) 125.0231(57%) | Procyanidin B2-di-O-gallate |  |
| B21-3/A60-3 | 16.10 | 881.1581 | 1.173 | C_44_H_33_O_20_ | 577.1356(10%) 559.1268(20%) 541.1158(10%) 407.0770(100%) 289.0721(65%) 245.0451(12%) 169.0132(26%) 161.0233(30%) 137.0232(12%) 125.0231(57%) | Procyanidin B2-di-O-gallate |  |
| B21-4/A60-4 | 16.54 | 881.1581 | 1.173 | C_44_H_33_O_20_ | 577.1356(10%) 559.1268(20%) 541.1158(10%) 407.0770(100%) 289.0721(65%) 245.0451(12%) 169.0132(26%) 161.0233(30%) 137.0232(12%) 125.0231(57%) | Procyanidin B2-di-O-gallate |  |
| B21-5/A60-5 | 17.27 | 881.1581 | 1.173 | C_44_H_33_O_20_ | 577.1356(10%) 559.1268(20%) 541.1158(10%) 407.0770(100%) 289.0721(65%) 245.0451(12%) 169.0132(26%) 161.0233(30%) 137.0232(12%) 125.0231(57%) | Procyanidin B2-di-O-gallate |  |
| B21-6/A60-6 | 18.42 | 881.1581 | 1.173 | C_44_H_33_O_20_ | 577.1356(10%) 559.1268(20%) 541.1158(10%) 407.0770(100%) 289.0721(65%) 245.0451(12%) 169.0132(26%) 161.0233(30%) 137.0232(12%) 125.0231(57%) | Procyanidin B2-di-O-gallate |  |
| B21-7/A60-7 | 20.29 | 881.1581 | 1.173 | C_44_H_33_O_20_ | 577.1356(10%) 559.1268(20%) 541.1158(10%) 407.0770(100%) 289.0721(65%) 245.0451(12%) 169.0132(26%) 161.0233(30%) 137.0232(12%) 125.0231(57%) | Procyanidin B2-di-O-gallate |  |
| B22-1/ A61-1 | 11.12 | 897.1533 | 1.47 | C_44_H_33_O_21_ | 407.0767(100%) 289.0720(65%)245.0810(26%) 177.0183(40%) 169.0135(28%) 137.0224(15%) 125.0230(38%) | (-)-epicatechin 3-O-gallate (4β-8)-(-)-epigallocatechin 3-O-gallate |  |
| B22-2/ A61-2 | 11.6 | 897.1533 | 1.47 | C_44_H_33_O_21_ | 407.0767(100%) 289.0720(65%)245.0810(26%) 177.0183(40%) 169.0135(28%) 137.0224(15%) 125.0230(38%) | (-)-epicatechin 3-O-gallate (4β-8)-(-)-epigallocatechin 3-O-gallate |  |
| B22-3/ A61-3 | 11.73 | 897.1533 | 1.47 | C_44_H_33_O_21_ | 407.0767(100%) 289.0720(65%)245.0810(26%) 177.0183(40%) 169.0135(28%) 137.0224(15%) 125.0230(38%) | (-)-epicatechin 3-O-gallate (4β-8)-(-)-epigallocatechin 3-O-gallate |  |
| B23-1/ A62-1 | 8.00 | 1017.2111 | 1.577 | C_52_H_41_O_22_ | 677.1306(15%) 525.0829(20%) 451.1047(20%) 407.0766(80%) 289.0720(65%) 245.0810(26%) 177.0183(10%) 169.0135(15%) 161.0235(30%) 137.0224(15%) 125.0230(100%) | [Epicatechin-(4β-&gt;8)]2-epicatechin 3'''-gallate |  |
| B23-2/ A62-2 | 8.73 | 1017.2111 | 1.577 | C_52_H_41_O_22_ | 677.1306(15%) 525.0829(20%) 451.1047(20%) 407.0766(80%) 289.0720(65%) 245.0810(26%) 177.0183(10%) 169.0135(15%) 161.0235(30%) 137.0224(15%) 125.0230(100%) | [Epicatechin-(4β-&gt;8)]2-epicatechin 3'''-gallate |  |
| B23-3/ A62-3 | 9.62 | 1017.2111 | 1.577 | C_52_H_41_O_22_ | 677.1306(15%) 525.0829(20%) 451.1047(20%) 407.0766(80%) 289.0720(65%) 245.0810(26%) 177.0183(10%) 169.0135(15%) 161.0235(30%) 137.0224(15%) 125.0230(100%) | [Epicatechin-(4β-&gt;8)]2-epicatechin 3'''-gallate |  |
| B23-4/ A62-4 | 11.05 | 1017.2111 | 1.577 | C_52_H_41_O_22_ | 677.1306(15%) 525.0829(20%) 451.1047(20%) 407.0766(80%) 289.0720(65%) 245.0810(26%) 177.0183(10%) 169.0135(15%) 161.0235(30%) 137.0224(15%) 125.0230(100%) | [Epicatechin-(4β-&gt;8)]2-epicatechin 3'''-gallate |  |
| B23-5/ A62-5 | 11.34 | 1017.2111 | 1.577 | C_52_H_41_O_22_ | 677.1306(15%) 525.0829(20%) 451.1047(20%) 407.0766(80%) 289.0720(65%) 245.0810(26%) 177.0183(10%) 169.0135(15%) 161.0235(30%) 137.0224(15%) 125.0230(100%) | [Epicatechin-(4β-&gt;8)]2-epicatechin 3'''-gallate |  |
| B23-6/ A62-6 | 11.67 | 1017.2111 | 1.577 | C_52_H_41_O_22_ | 677.1306(15%) 525.0829(20%) 451.1047(20%) 407.0766(80%) 289.0720(65%) 245.0810(26%) 177.0183(10%) 169.0135(15%) 161.0235(30%) 137.0224(15%) 125.0230(100%) | [Epicatechin-(4β-&gt;8)]2-epicatechin 3'''-gallate |  |
| B23-7/ A62-7 | 12.42 | 1017.2111 | 1.577 | C_52_H_41_O_22_ | 677.1306(15%) 525.0829(20%) 451.1047(20%) 407.0766(80%) 289.0720(65%) 245.0810(26%) 177.0183(10%) 169.0135(15%) 161.0235(30%) 137.0224(15%) 125.0230(100%) | [Epicatechin-(4β-&gt;8)]2-epicatechin 3'''-gallate |  |
| B23-8/ A62-8 | 14.19 | 1017.2111 | 1.577 | C_52_H_41_O_22_ | 677.1306(15%) 525.0829(20%) 451.1047(20%) 407.0766(80%) 289.0720(65%) 245.0810(26%) 177.0183(10%) 169.0135(15%) 161.0235(30%) 137.0224(15%) 125.0230(100%) | [Epicatechin-(4β-&gt;8)]2-epicatechin 3'''-gallate |  |
| B23-9/ A62-9 | 15.12 | 1017.2111 | 1.577 | C_52_H_41_O_22_ | 677.1306(15%) 525.0829(20%) 451.1047(20%) 407.0766(80%) 289.0720(65%) 245.0810(26%) 177.0183(10%) 169.0135(15%) 161.0235(30%) 137.0224(15%) 125.0230(100%) | [Epicatechin-(4β-&gt;8)]2-epicatechin 3'''-gallate |  |
| B23-10/ A62-10 | 17.69 | 1017.2111 | 1.577 | C_52_H_41_O_22_ | 677.1306(15%) 525.0829(20%) 451.1047(20%) 407.0766(80%) 289.0720(65%) 245.0810(26%) 177.0183(10%) 169.0135(15%) 161.0235(30%) 137.0224(15%) 125.0230(100%) | [Epicatechin-(4β-&gt;8)]2-epicatechin 3'''-gallate |  |
| B24-1 | 6.51 | 1153.2628 | 0.484 | C_60_H_49_O_24_ | 407.0770(45%) 289.0720(42%) 287.0568(50%) 243.0298(35%) 161.0233(30%) 125.0230(100%) | cinnamtannin A2 |  |
| B24-2 | 10.29 | 1153.2628 | 0.484 | C_60_H_49_O_24_ | 407.0770(45%) 289.0720(42%) 287.0568(50%) 243.0298(35%) 161.0233(30%) 125.0230(100%) | cinnamtannin A2 |  |
| B24-3 | 11.59 | 1153.2628 | 0.484 | C_60_H_49_O_24_ | 407.0770(45%) 289.0720(42%) 287.0568(50%) 243.0298(35%) 161.0233(30%) 125.0230(100%) | cinnamtannin A2 |  |
| B25-1/ A63-1 | 11.67 | 1169.2216 | 0.98 | C_59_H_45_O_26_ | 677.1306(40%) 559.1238(35%) 525.0829(20%) 451.1047(20%) 433.0940(40%) 407.0766(55%) 395.0771(70%) 289.0720(65%) 287.0559(75%) 269.0459(75%) 245.0810(26%) 169.0135(20%) 161.0235(30%) 137.0224(22%) 125.0230(100%) | procyanidin C-1 3',3''-di-O-gallate |  |
| B25-2/ A63-2 | 13.85 | 1169.2216 | 0.98 | C_59_H_45_O_26_ | 677.1306(40%) 559.1238(35%) 525.0829(20%) 451.1047(20%) 433.0940(40%) 407.0766(55%) 395.0771(70%) 289.0720(65%) 287.0559(75%) 269.0459(75%) 245.0810(26%) 169.0135(20%) 161.0235(30%) 137.0224(22%) 125.0230(100%) | procyanidin C-1 3',3''-di-O-gallate |  |
| B25-3/ A63-3 | 15.14 | 1169.2216 | 0.98 | C_59_H_45_O_26_ | 677.1306(40%) 559.1238(35%) 525.0829(20%) 451.1047(20%) 433.0940(40%) 407.0766(55%) 395.0771(70%) 289.0720(65%) 287.0559(75%) 269.0459(75%) 245.0810(26%) 169.0135(20%) 161.0235(30%) 137.0224(22%) 125.0230(100%) | procyanidin C-1 3',3''-di-O-gallate |  |
| B25-4/ A63-4 | 16.09 | 1169.2216 | 0.98 | C_59_H_45_O_26_ | 677.1306(40%) 559.1238(35%) 525.0829(20%) 451.1047(20%) 433.0940(40%) 407.0766(55%) 395.0771(70%) 289.0720(65%) 287.0559(75%) 269.0459(75%) 245.0810(26%) 169.0135(20%) 161.0235(30%) 137.0224(22%) 125.0230(100%) | procyanidin C-1 3',3''-di-O-gallate |  |
| B25-5/ A63-5 | 17.33 | 1169.2216 | 0.98 | C_59_H_45_O_26_ | 677.1306(40%) 559.1238(35%) 525.0829(20%) 451.1047(20%) 433.0940(40%) 407.0766(55%) 395.0771(70%) 289.0720(65%) 287.0559(75%) 269.0459(75%) 245.0810(26%) 169.0135(20%) 161.0235(30%) 137.0224(22%) 125.0230(100%) | procyanidin C-1 3',3''-di-O-gallate |  |
