# Supplementary material for: Processing of Reynoutria multiflora: transformation of catechin and gallic acid derivatives and their identification
Source: Front Pharmacol. 2024 Feb 26;15:1356876. doi: 10.3389/fphar.2024.1356876 (PMC10926517; doi:10.3389/fphar.2024.1356876)
Supplement: Supplementary file 2 [file Table2.DOCX]

Supplementary Table 2

Table 2.1 producer, content determination and processing usage of *Polygonum multiflorum* pieces

| Producer | Decoction piece | | processing mate | 2, 3, 5, 4 '- tetrahydroxystilbene-8-O-glucoside（Chinese pharmacopoeia≥1.0%） | combined anthraquinones  （Chinese pharmacopoeia≥0.10%） |
| --- | --- | --- | --- | --- | --- |
|  | Batch number | weight（kg） | black soybean（kg） |  |  |
| Deqing Guangdong | 2022022170-S | 30 | 3 | 2.2 | 0.25 |
|  | 2022022470-S | 30 | 3 | 2.4 | 0.38 |
|  | 2022022870-S | 30 | 3 | 2.6 | 0.26 |
| Miyi, Sichuang | 2022062170-S | 30 | 3 | 3.2 | 0.19 |
|  | 2022062470-S | 30 | 3 | 2.6 | 0.18 |
|  | 2022062770-S | 30 | 3 | 2.9 | 0.21 |
| Kaili, Guizhou | 2022030370-S | 30 | 3 | 2.3 | 0.25 |
|  | 2022030770-S | 30 | 3 | 2.1 | 0.23 |
|  | 2022031070-S | 30 | 3 | 1.9 | 0.14 |

Table 2.2 *Polygonum multiflorum* processed time, batch numbers and picture information table

| producer | Batch number of medicinal herbs | Steaming time | batch number | Fig. |
| --- | --- | --- | --- | --- |
| Deqing Guangdong | DH22012401 | 0h | 2022022170-S | 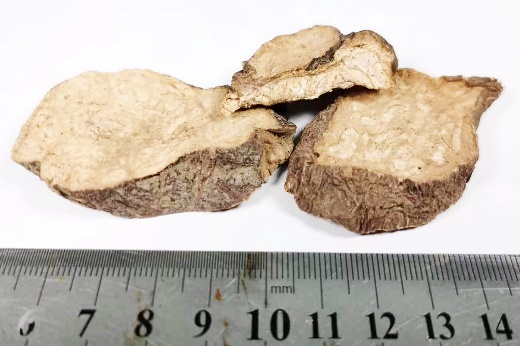 |
|  |  | 4h | 2022022170-4H | 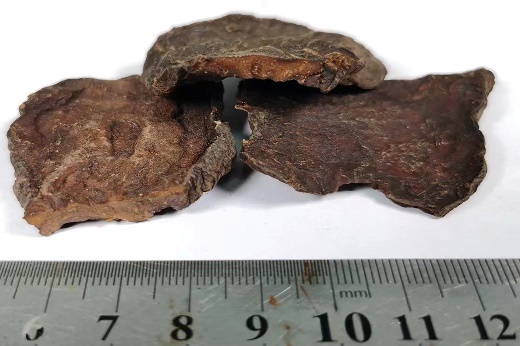 |
|  |  | 8h | 2022022170-8H | 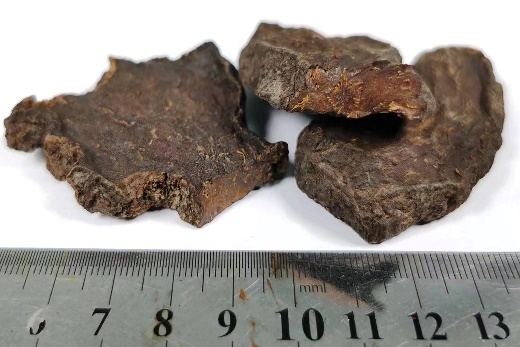 |
|  |  | 12h | 2022022170-12H | 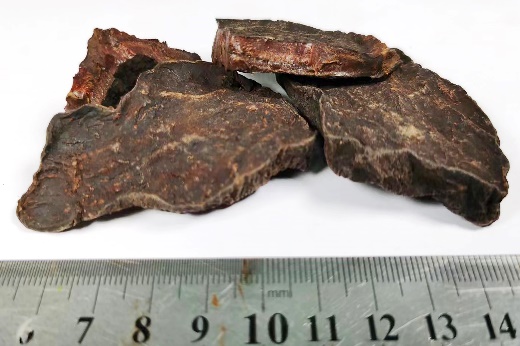 |
|  |  | 18h | 2022022170-18H | 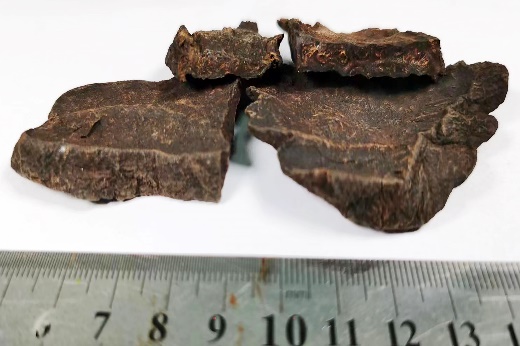 |
|  |  | 24h | 2022022170-24H | 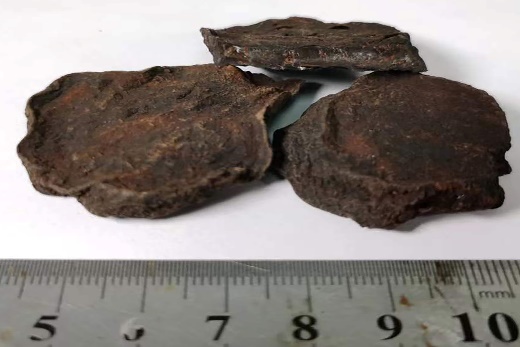 |
|  |  | 32h | 2022022170-32H | 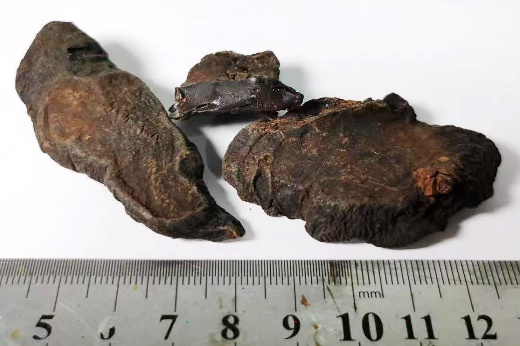 |
|  | DH22012402 | 0h | 2022022470-S | 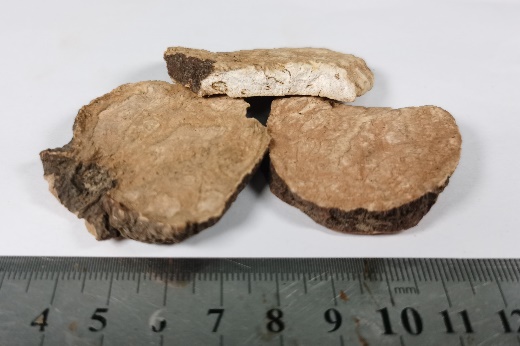 |
|  |  | 4h | 2022022470-4H | 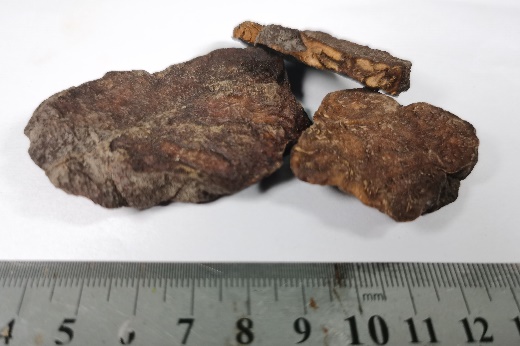 |
|  |  | 8h | 2022022470-8H | 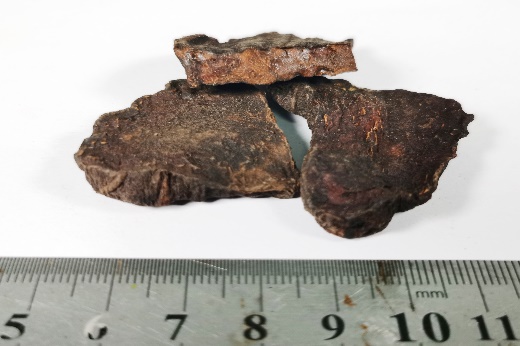 |
|  |  | 12h | 2022022470-12H | 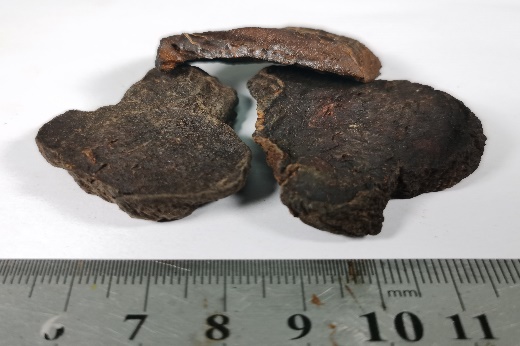 |
|  |  | 18h | 2022022470-18H | 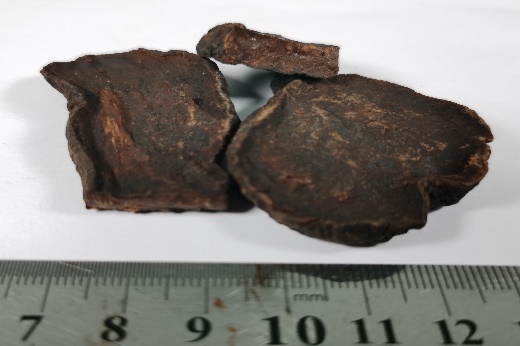 |
|  |  | 24h | 2022022470-24H | 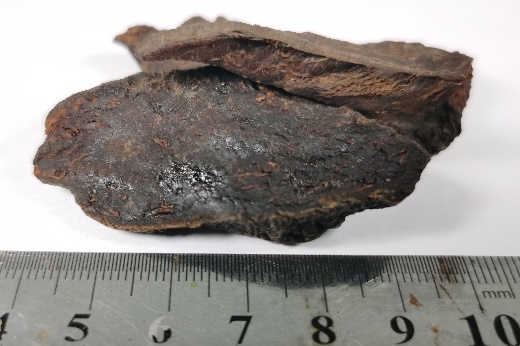 |
|  |  | 32h | 2022022470-32H | 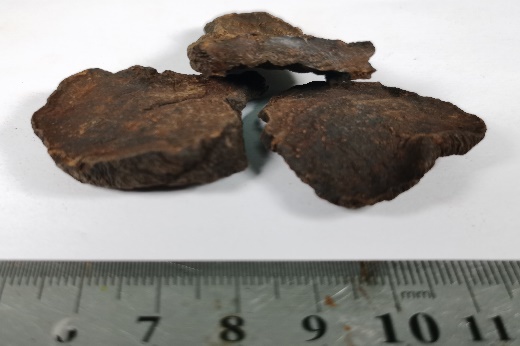 |
|  | DH22012403 | 0h | 2022022870-S | 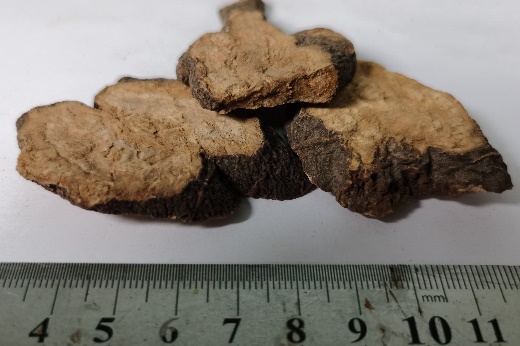 |
|  |  | 4h | 2022022870-4H | 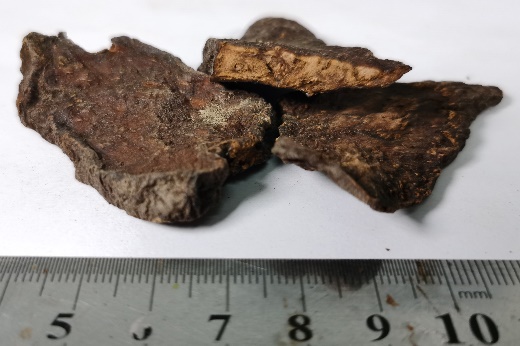 |
|  |  | 8h | 2022022870-8H | 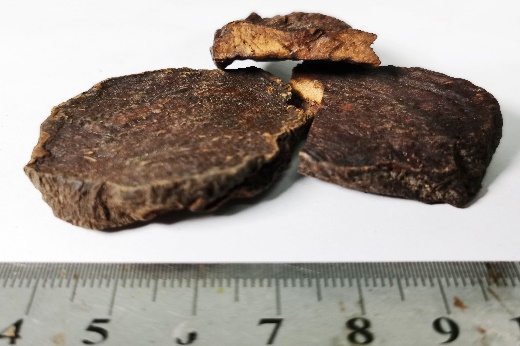 |
|  |  | 12h | 2022022870-12H | 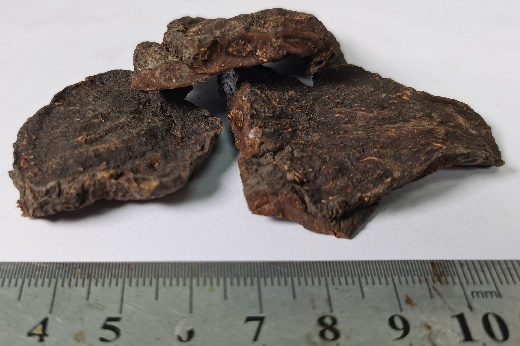 |
|  |  | 18h | 2022022870-18H | 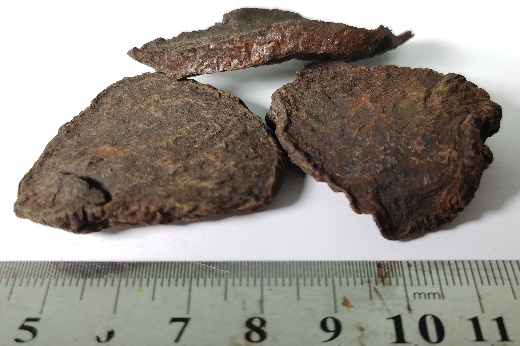 |
|  |  | 24h | 2022022870-24H | 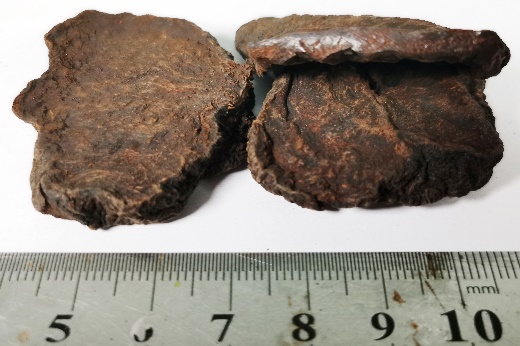 |
|  |  | 32h | 2022022870-32H | 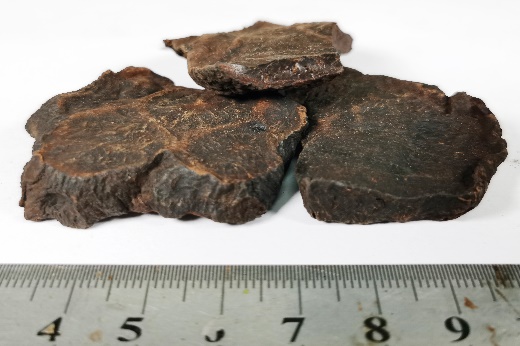 |
| Kaili, Guizhou | DH22012601 | 0h | 2022030370-S | 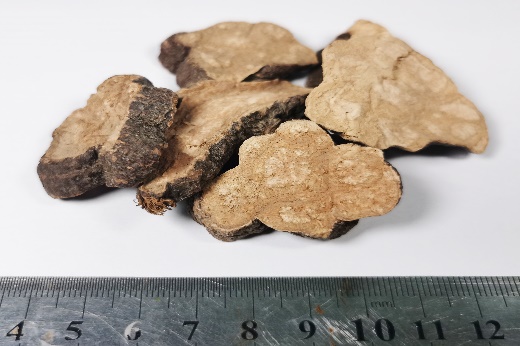 |
|  |  | 4h | 2022030370-4H | 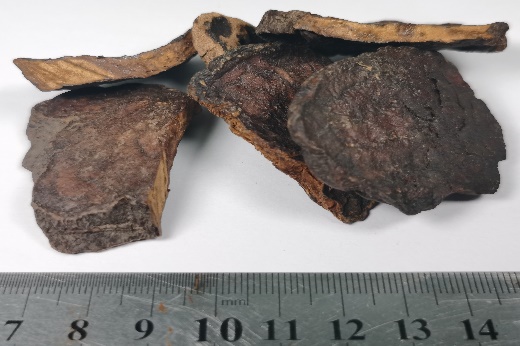 |
|  |  | 8h | 2022030370-8H | 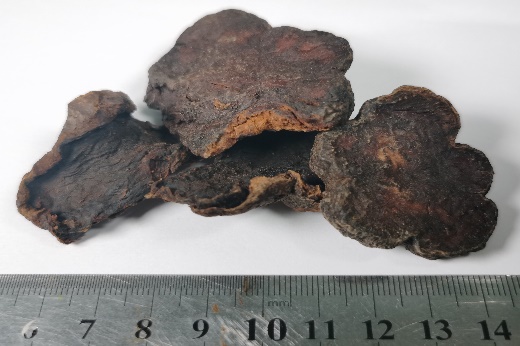 |
|  |  | 12h | 2022030370-12H | 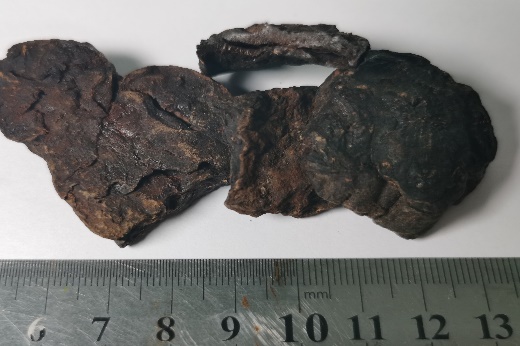 |
|  |  | 18h | 2022030370-18H | 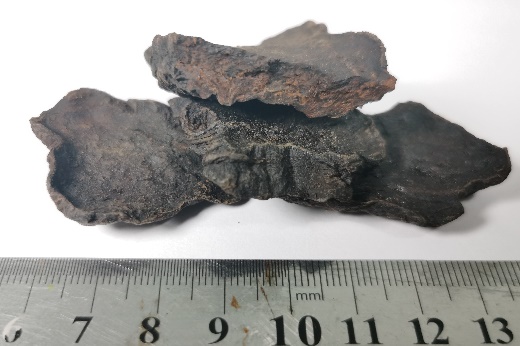 |
|  |  | 24h | 2022030370-24H | 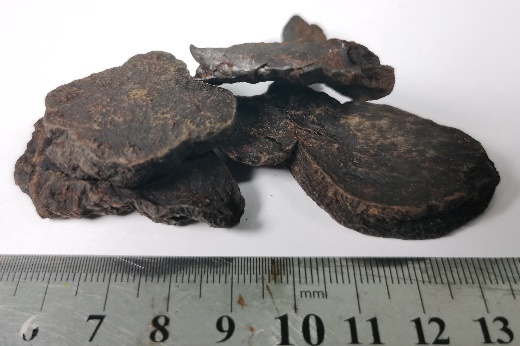 |
|  |  | 32h | 2022030370-32H | 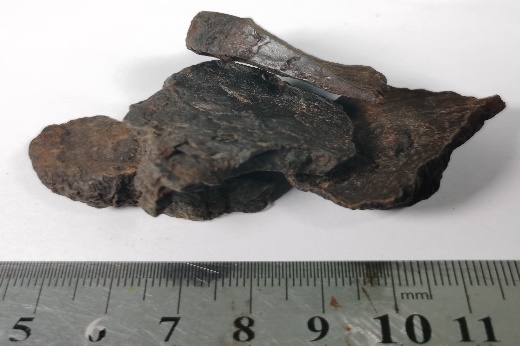 |
|  | DH22012602 | 0h | 2022030770-S | 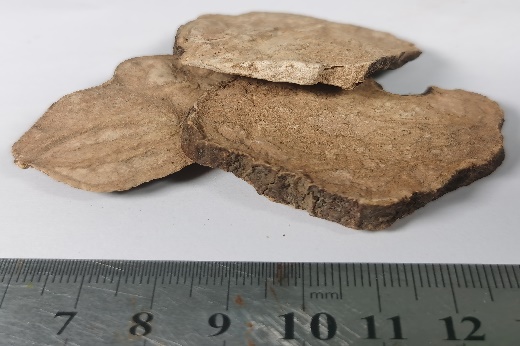 |
|  |  | 4h | 2022030770-4H | 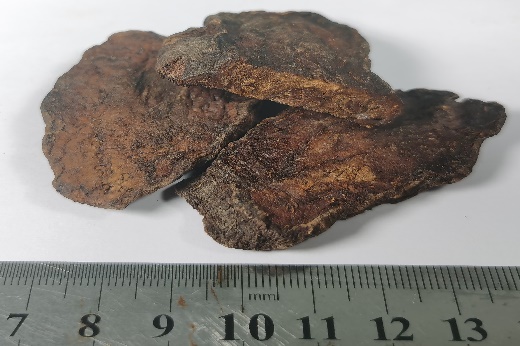 |
|  |  | 8h | 2022030770-8H | 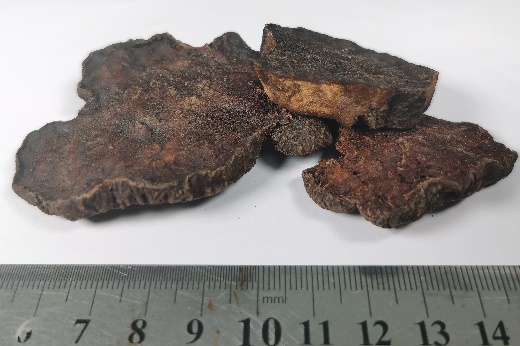 |
|  |  | 12h | 2022030770-12H | 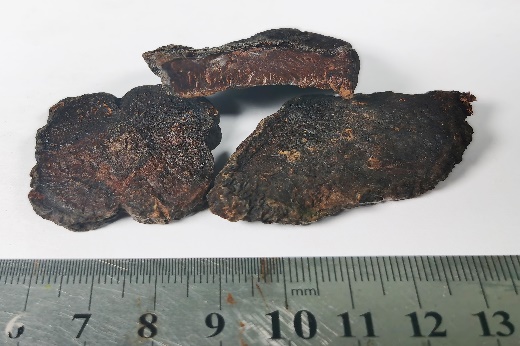 |
|  |  | 18h | 2022030770-18H | 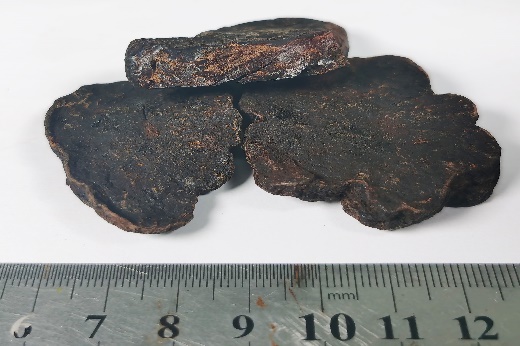 |
|  |  | 24h | 2022030770-24H | 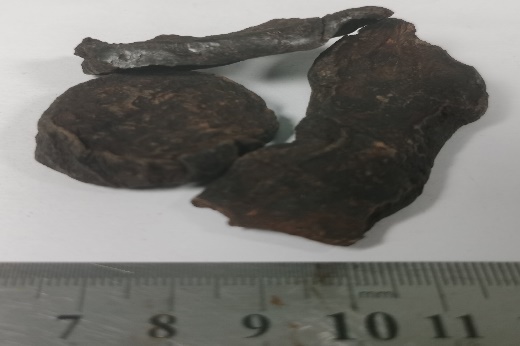 |
|  |  | 32h | 2022030770-32H | 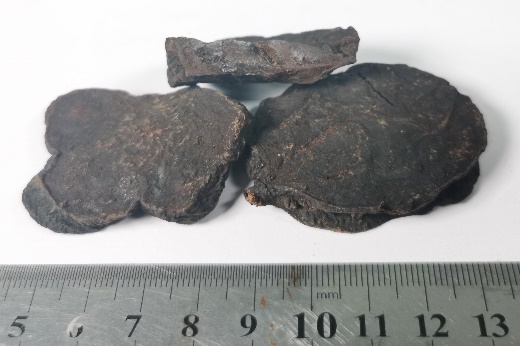 |
|  | DH22012603 | 0h | 2022031070-S | 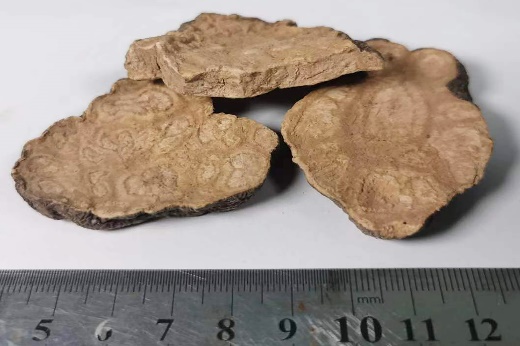 |
|  |  | 4h | 2022031070-4H | 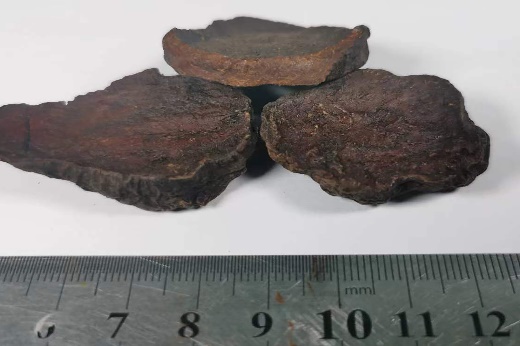 |
|  |  | 8h | 2022031070-8H | 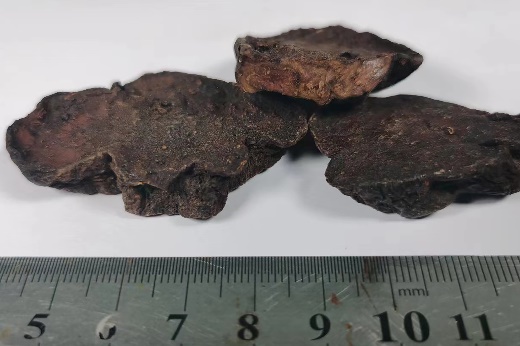 |
|  |  | 12h | 2022031070-12H | 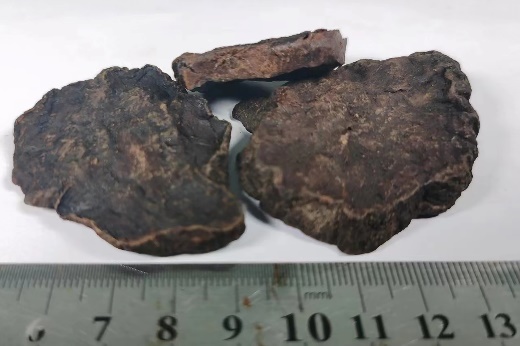 |
|  |  | 18h | 2022031070-18H | 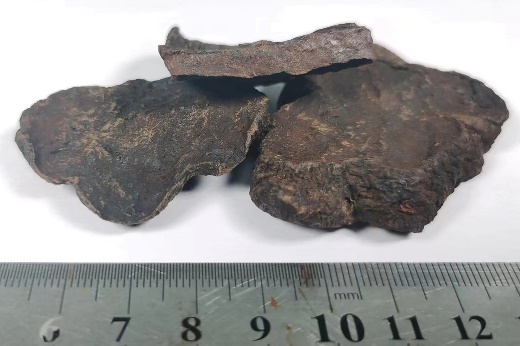 |
|  |  | 24h | 2022031070-24H | 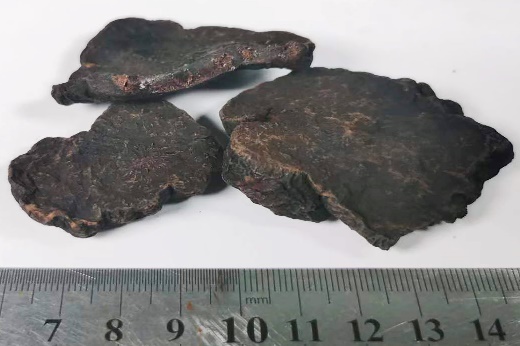 |
|  |  | 32h | 2022031070-32H | 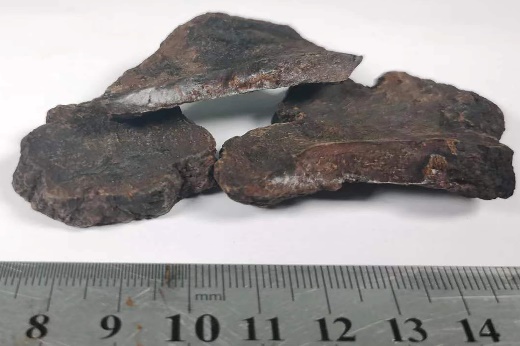 |
| Miyi, Sichuang | DH22061801 | 0h | 2022062170-S | 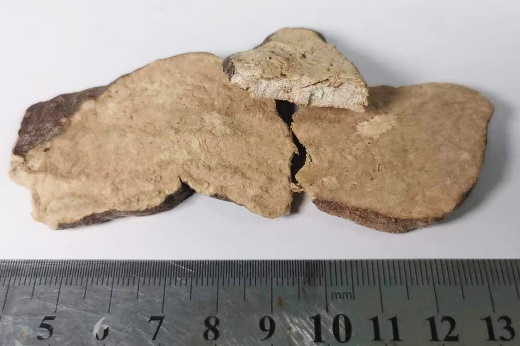 |
|  |  | 4h | 2022062170-4H | 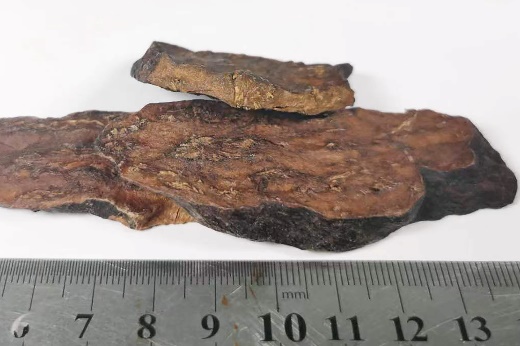 |
|  |  | 8h | 2022062170-8H | 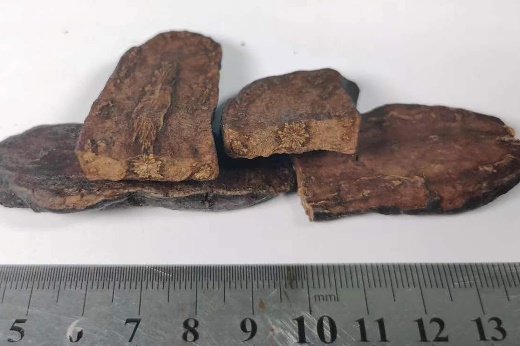 |
|  |  | 12h | 2022062170-12H | 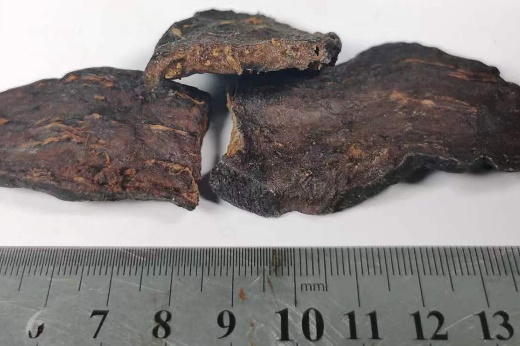 |
|  |  | 18h | 2022062170-18H | 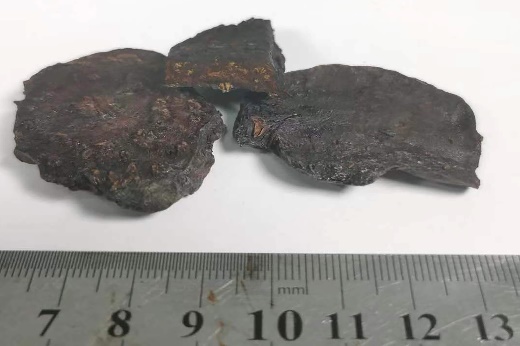 |
|  |  | 24h | 2022062170-24H | 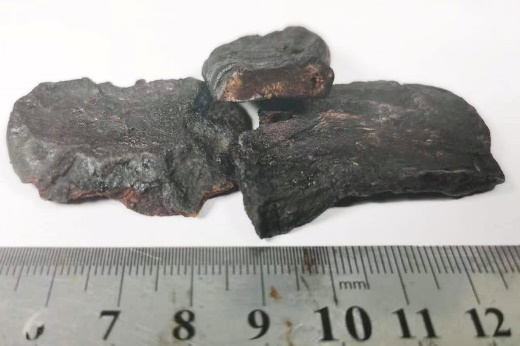 |
|  |  | 32h | 2022062170-32H | 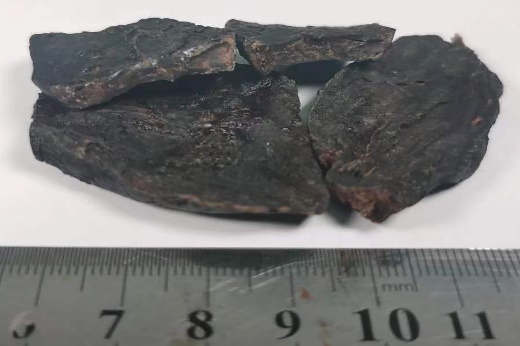 |
|  | DH22061802 | 0h | 2022062470-S | 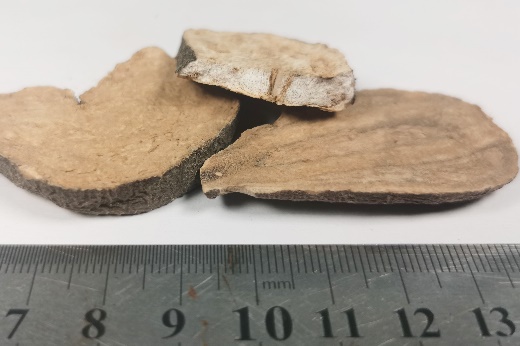 |
|  |  | 4h | 2022062470-4H | 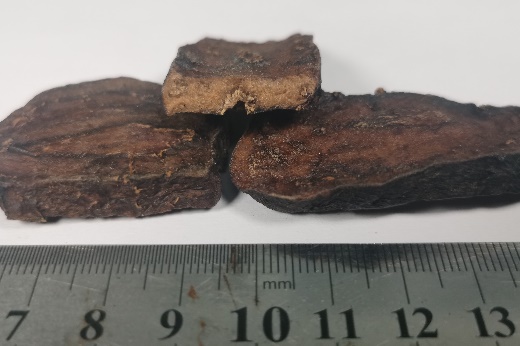 |
|  |  | 8h | 2022062470-8H | 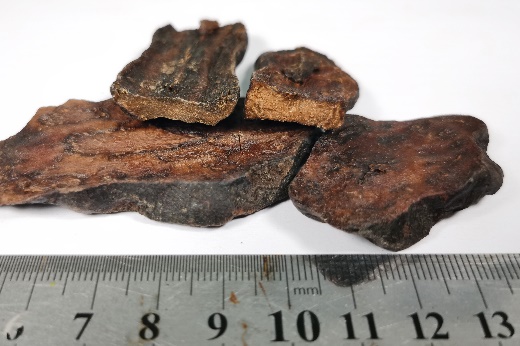 |
|  |  | 12h | 2022062470-12H | 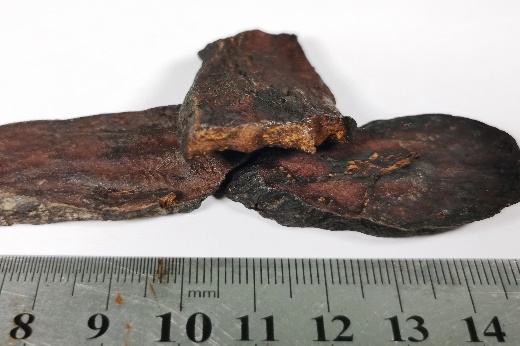 |
|  |  | 18h | 2022062470-18H | 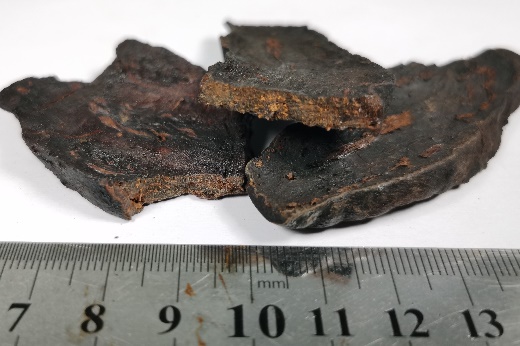 |
|  |  | 24h | 2022062470-24H | 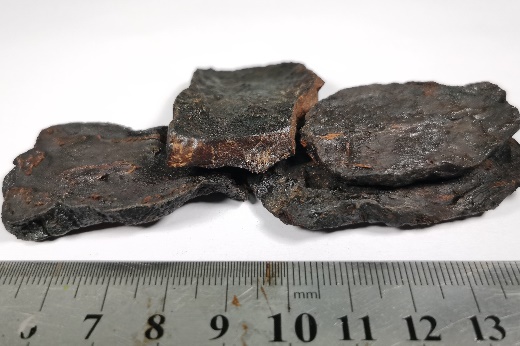 |
|  |  | 32h | 2022062470-32H | 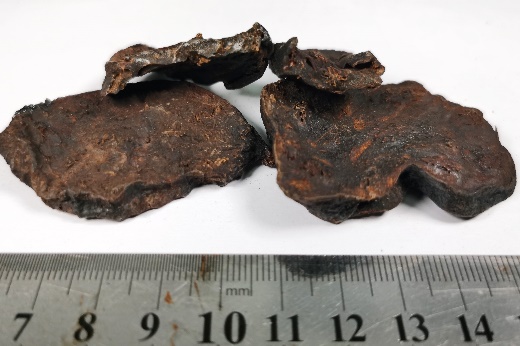 |
|  | DH22061803 | 0h | 2022062770-S | 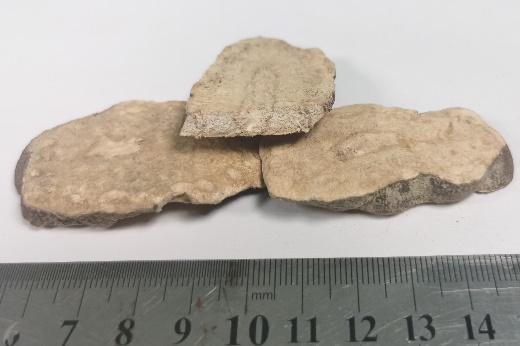 |
|  |  | 4h | 2022062770-4H | 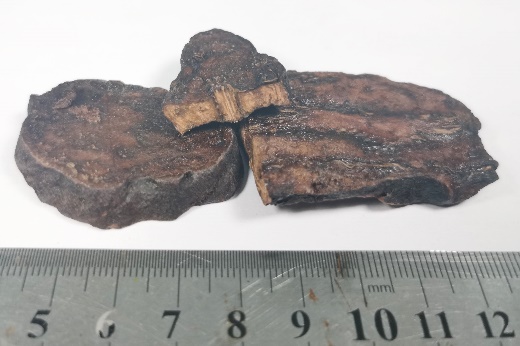 |
|  |  | 8h | 2022062770-8H | 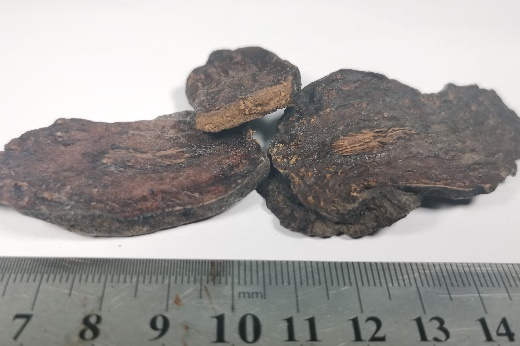 |
|  |  | 12h | 2022062770-12H | 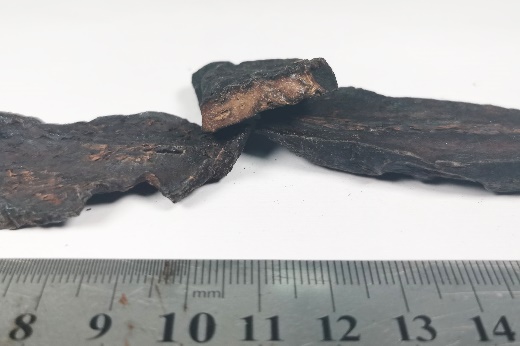 |
|  |  | 18h | 2022062770-18H | 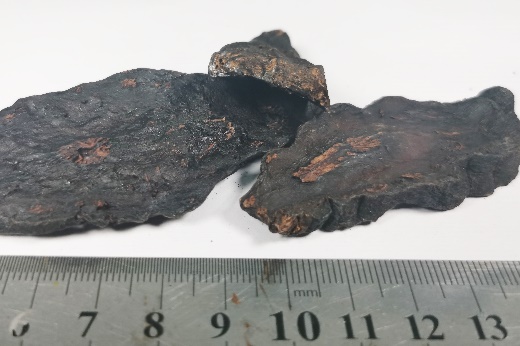 |
|  |  | 24h | 2022062770-24H | 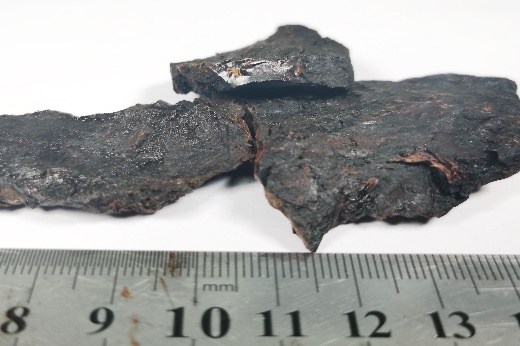 |
|  |  | 32h | 2022062770-32H | 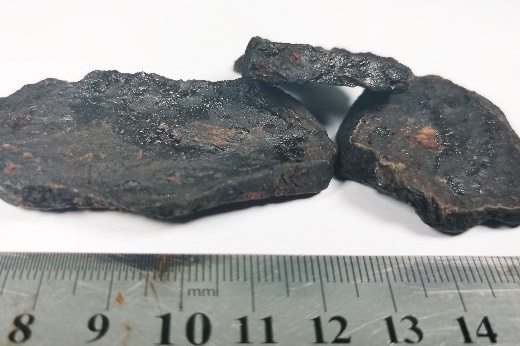 |
